# Semirenewable Polyamides Containing Disulfide Bonds: Synthesis, Degradation, Self-Healing, and Triboelectric Properties

**DOI:** 10.1021/acs.macromol.5c02730

**Published:** 2025-11-14

**Authors:** Pavel S. Kulyabin, Alejandra Sophia Lozano-Pérez, Tianhuai Xu, Yogeshwar D. More, Harini Sampathkumar, Ketan Pancholi, Oliver Page, Chloe Rennie, Lea Hämmerling, Kelly Lima, Eli Zysman-Colman, Jin-Chong Tan, Amit Kumar

**Affiliations:** † EaStCHEM, School of Chemistry, 7486 University of St Andrews, North Haugh, St Andrews KY16 9ST, U.K.; ‡ Multifunctional Materials and Composites (MMC) Laboratory, Department of Engineering Science, 6396 University of Oxford, Parks Road, Oxford OX1 3PJ, U.K.; § The Sir Ian Wood Building, 1018 Robert Gordon University, Garthdee Rd, Garthdee, Aberdeen AB10 7GE, U.K.; ∥ Organic Semiconductor Centre, EaStCHEM School of Chemistry, University of St Andrews, St Andrews KY16 9ST, U.K.

## Abstract

We report here the synthesis, degradation, and properties of polyamides containing disulfide bonds. The polyamides have been prepared using a two-step melt polycondensation process from 4,4′-dithiodibutyric acid and bioderived Priamine. The degradation of these polymers has been investigated using a combination of tools, such as visible light photocatalysis, and UV-mediated degradation. The chemical, physical, and mechanical properties of these polymers were also studied. The disulfide-containing polymers exhibit elastomeric and self-healing properties while showing high thermal stability. Furthermore, the novel application of these unique tribopositive polymers as self-repairable triboelectric nanogenerators for energy harvesting has also been demonstrated.

## Graphical abstract

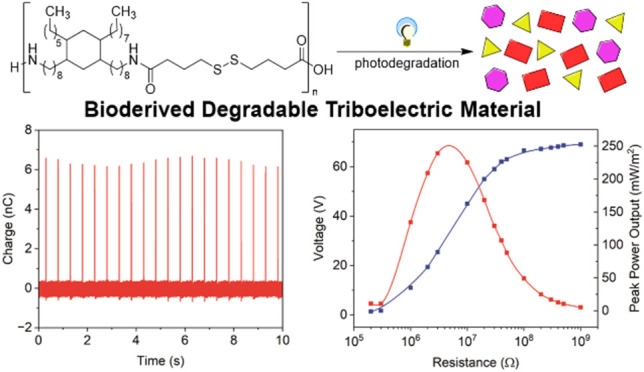

## Introduction

Polyamides have played a pivotal role in the development of modern materials since their invention in the 1930s.^1^ First synthesized by Wallace Carothers at DuPont,^2^ these versatile polymers quickly revolutionized industries ranging from textiles to automotive manufacturing. The unique combination of strength, durability, and flexibility offered by polyamides made them indispensable in applications where traditional materials fell short. Throughout the 20th century, polyamides continued to evolve, with variations like aramids^3^ (e.g., Kevlar) finding use in applications such as bulletproof vests and aerospace components.^4^ Today, polyamides remain at the forefront of materials science, with ongoing research into new formulations and applications continually expanding their utility in our daily lives.^5^

Recently, the focus of research has shifted toward developing biobased and sustainable polyamides to address environmental concerns while maintaining the exceptional properties that make these materials so crucial to modern society.^6,7^ Biobased polyamides represent a sustainable alternative to traditional petroleum-based derivatives, as they are synthesized from renewable resources such as castor oil, corn, and other plant-based feedstocks.^8^ Several of these eco-friendly polymers, such as Nymax BIO from Avient, TERRYL by Cathay Biotech, EcoPaXX from Envalior, and Enka Nylon BIO from Indorama, have been commercialized in recent years and found use in various industries, including automotive, textiles, and packaging, offering comparable performance to their petrochemical-derived counterparts while boasting a lower carbon footprint.^9^

At the same time, polyamides have significantly contributed to the global plastics pollution crisis.^10^ Since these materials are exceptionally durable, their resistance to degradation has led to their persistence in the environment, particularly in marine ecosystems.^11^ The accumulation of polyamide-based products, such as fishing nets and textiles, in oceans and landfills poses a severe threat to wildlife and ecosystems.^12^ While awareness of plastic pollution grows, and traditional mechanical recycling methods lead to downcycled material,^13,14^ there is an urgent need to simultaneously develop sustainable alternatives and improved recycling technologies for polyamides to mitigate their environmental impact.^15^

Degradable and renewable polyamides present a promising solution to the environmental challenges posed by traditional materials.^13,16,17^ These polymers can be designed to break down under specific conditions while maintaining their desirable properties during use.^17^ For example, incorporating susceptible groups or triggers into the polymer backbone that can be activated using a catalyst or energy source can lead to the design of a degradable polymer.^18,19,17,20–22^

Along this line, self-healing polymers have also been a subject of intense research for over two decades, but examples of self-healing polyamide-based elastomers remain limited.^23^ Early attempts, such as the hybrid network-based elastomer by Weitz et al., showed only partial recovery of tensile strength due to permanent covalent cross-links.^24^ Chen, and Yao et al. made significant progress with hydrogen bond-based polyamide elastomers, achieving over 80% in self-healing efficiency at room temperature.^25–27^ However, the incorporation of dynamic covalent bonds, which could provide both self-healing ability and multiple stimulus response characteristics, is still rare in polyamide-based elastomers.^28,29^ While Wang and co-workers reported promising results with elastomers based on olefin cross-metathesis and hydrogen bonds, the complex catalyst synthesis process hinders large-scale application.^30^ Consequently, there is a pressing need for practical methods to incorporate dynamic or reversible covalent bonds into polyamide-based elastomer systems, which could enhance their self-healing capabilities and broaden their applications. Considering significant progress made in exploiting the reversible formation of disulfide bonds in the design of various self-healing polymers,^31–33^ we envisioned that a disulfide-containing polyamide could be self-healing and degradable under mild conditions. Here, we present our studies on the synthesis and properties of disulfide-containing nylons and their potential application in triboelectric nanogenerators (TENG), which act as a unique tribopositive material (**PS-2**). It is crucial to acknowledge that the lack of self-healing materials in TENGs substantially restricts their long-term efficiency, power output, and operational lifespan, presenting significant challenges for sustainable energy harvesting applications.^34,35^ The integration of self-healing capabilities could help in ensuring durability and reusability and reducing maintenance requirements. In this context, disulfide-containing polyamide-based elastomers could be of high significance for TENG-derived applications.

## Results and Discussion

We started our investigation by developing the synthesis of polyamides using a two-step melt polycondensation reaction reported previously.^28,29^ We chose 4,4′-dithiodibutyric acid (DTDBA) as one of the monomers to introduce disulfide bonds in the polymer chain. Priamines 1074 and 1075, which are biobased diamines, were chosen as the other monomers. They are made from fatty acids and contain a varying mixture of cyclic and acyclic isomers of diamines containing long-chain alkyl substituents.^36^ As shown in Table 1, the polycondensation of 4,4′-dithiodibutyric acid (DTDBA) and Priamines 1074 or 1075 led to the formation of polyamides **PS-1** and **PS-2**, respectively. To probe the effect of disulfide bond incorporation on the properties of the polymer, two reference polyamides **PA-1** and **PA-2** were made using identical reaction conditions from the coupling of sebacic acid and Priamines 1074 and 1075. Additionally, another reference polyamide, **PA-3**, was obtained from the polycondensation of DTDBA and linear 1,12-diaminododecane to understand the effect of Priamine. These five polyamides were characterized by spectroscopy (NMR, IR), GPC (gel permeation chromatography), TGA (thermogravimetric analysis), and DSC (differential scanning calorimetry). The infrared (IR) spectra of **PA-2** and **PS-2** (Figure 1A) were found to be almost identical, likely due to the disulfide bonds being IR inactive. The IR bands ν_N–H_ = 3302 cm^–1^ and ν_C=O_ = 1638 cm^–1^ were found to be very close to those of nylon-6,6 ^37^ which exhibits IR signals at ν_N–H_ = 3298 cm^–1^ and ν_C=O_ = 1636 cm^–1^.

**Table 1:**
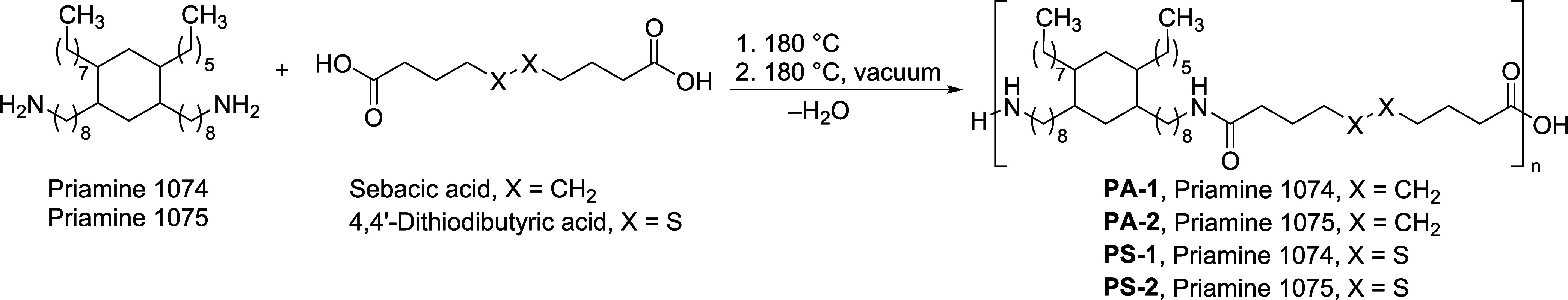
Synthesis of Polyamides from Biobased Diamines Priamine 1074 and 1075^a^

sample	acid	amine	*M*_n_,^b^ kg/mol	*M*_w_,^b^ kg/mol	PDI^b^	*T*_g_,^c^ °C	*T*_m_,^c^ °C	*T*_c_,^c^ °	*T*_d_,^d^ °C
**PS-1**	4,4′-dithiodibutyric acid	Priamine 1074	5.5	13.0	2.4	–14	-	-	307
**PS-2**	4,4′-dithiodibutyric acid	Priamine 1075	11.9	35.4	2.9	–12	-	-	302
**PA-1**	sebacic acid	Priamine 1074	10.9	33.6	3.0	–1	92	54	427
**PA-2**	sebacic acid	Priamine 1075	11.8	28.8	2.4	–9	94	54	432
**PA-3**	4,4′-dithiodibutyric acid	1,12-diaminododecane	n.d.^e^	n.d.^e^	n.d.^e^	-	163	n.d.	272

a Reaction conditions: Dicarboxylic acid (7.4 mmol) and diamine (7.4 mmol) were weighed and added to a 50 mL flask equipped with a magnetic stirrer. The flask was refilled with argon, and the reaction temperature was gradually increased to 180 °C, followed by stirring at this temperature for 2 h. Next, the polymerization reaction was continued for 1 h under reduced pressure (1 mbar). The reaction was cooled to room temperature and then frozen in liquid nitrogen, and the polymer was mechanically broken down to remove it from the flask.

b Measured by GPC in THF at 35 °C using polystyrene standards.

c Measured by DSC scanning at 10 °C/min.

d Measured by TGA scanning at 10 °C/min and quoted as the temperature of 5% mass loss.

e n.d. = not determined.

**Figure 1:**
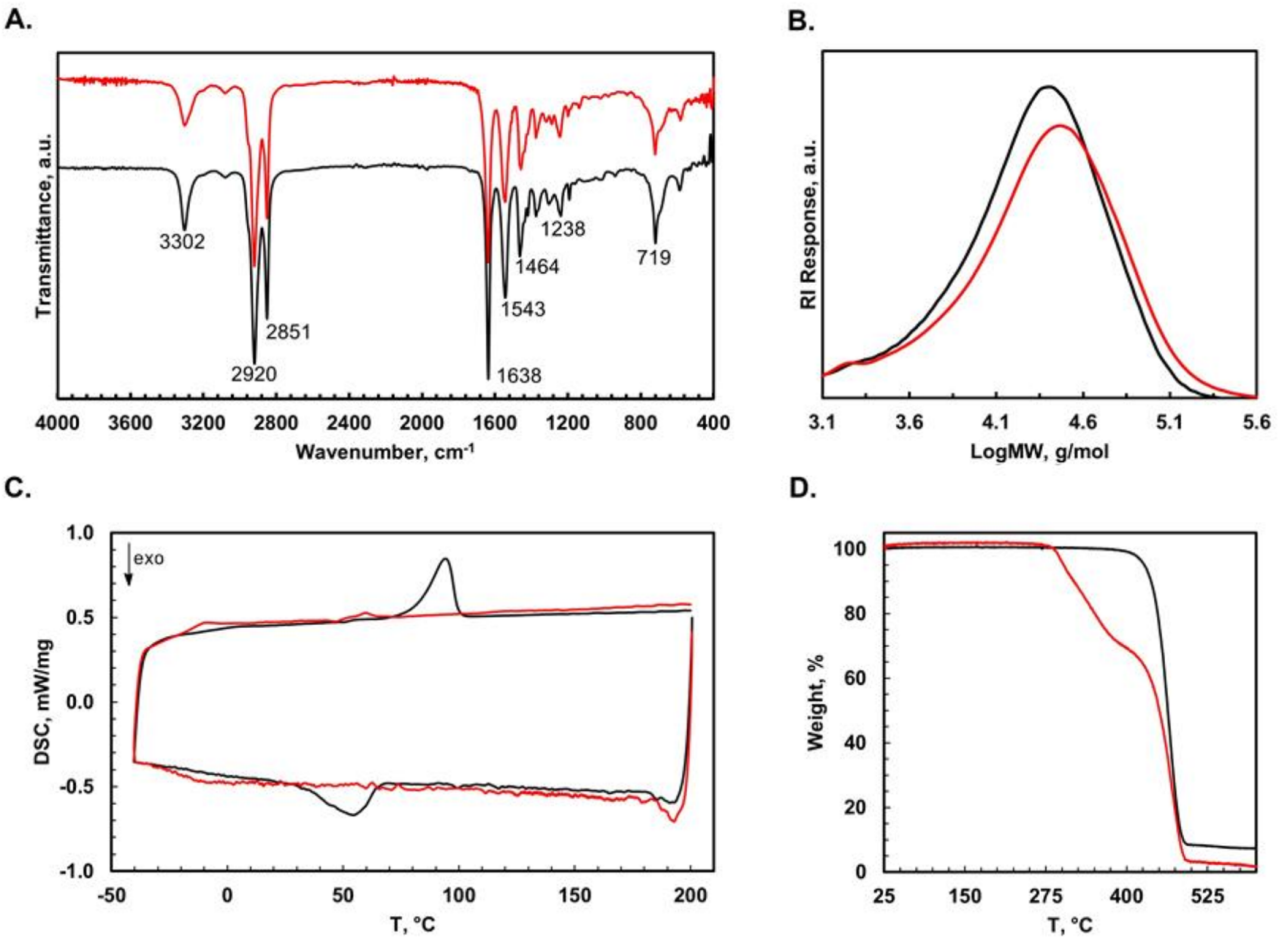
(A) FTIR (ATR) spectra of polyamides **PA-2** (black) and **PS-2** (red). (B) GPC slices of polyamides **PA-2** (black) and **PS-2** (red) in THF. (C) DSC traces of polyamides **PA-2** (black) and **PS-2** (red). (D) TGA of polyamides **PA-2** (black) and **PS-2** (red).

Polymer **PA-3** made from 1,12-diaminododecane was not soluble in organic solvents such as THF, DMF, DMSO, or chloroform, whereas polyamides made from Priamine were found to be soluble in THF, which allowed us to analyze these polymers using GPC. The number average molecular weights (*M*_n_) of these polymers were found to be in the range of 5.5–11.8 kg/mol, whereas the dispersities were found to be in the range of 2.4–3 (Table 1 and Figure 1B). Polymers **PA-1**, **PA-2**, **PS-1**, and **PS-2** were found to be elastomers, which could be due to the branching (long alkyl chains) present in the Priamine fragment, while **PA-3** was found to be highly crystalline, with a melting point of 163 °C. Polymers **PA-1** and **PA-2**, made of sebacic acid, had more crystalline components with melting points of 92 and 94 °C, respectively (Table 1), while **PS-1** and **PS-2** were found to be amorphous, with glass transition temperatures of −14 and −12 °C, respectively (Figure 1B). Thermogravimetric analysis (TGA) demonstrated that polyamides with disulfide moieties are less stable than sebacic acid-based analogues. Thus, **PS-2** significantly degrades at 302 °C, while **PA-2** is stable to 432 °C (Figure 1D, 5% mass loss). This result is unsurprising as the energy to break a disulfide bond is notably lower than that of a carbon–carbon bond.^38^ Indeed, TGA plots for **PS-1** and **PS-2** show two decomposition events, which we speculate are due to separate degradation events for S–S and C–C bonds.

Having synthesized these polymers, we explored various methods for their degradation. Disulfide bonds are known to participate in metathesis reactions where two different molecules can exchange fragments using ultrasound,^39^ UV irradiation,^40^ or nucleophile catalysis.^41^ We first used 1,4-diazabicyclo(2.2.2)­octane (DABCO), tricyclohexylphosphine,^41^ and triphenylphosphine as nucleophilic initiators for disulfide metathesis of **PS-2** (Table 2). Additionally, we chose CHCl_3_ as the solvent, as it can dissolve **PS-2** and has been used previously in phosphine-catalyzed metathesis reactions.^41^ We hypothesized that a 10-fold excess of DTDBA (4,4′-dithiodibutyric acid) could potentially split polymers into 11 fragments, producing smaller oligomers that could be used for the synthesis of a virgin polymer. Thus, the **PS-2** polymer was dissolved in CHCl_3_ to which 10 equiv of DTDBA was added along with 10, 50, or 100 mol % catalyst (DABCO, PCy_3_, PPh_3_), and the reaction mixture was heated at 100 °C for 19 h. As seen from the data in Table 2, using DABCO and Cy_3_P catalysts did not noticeably change the molecular weight of the polymer, while in the case of the Ph_3_P catalyst (10 mol %), the molecular weight (*M*_n_) of **PS-2** changed from 11.9 to 5.8 kg/mol. Increasing the loading of Ph_3_P promoted the degradation, with the molecular weight of the degradation product reaching 2.4 kDa (PDI = 2.4) when 100 mol % of Ph_3_P was used (Table 2, entry 9).

**Table 2:**
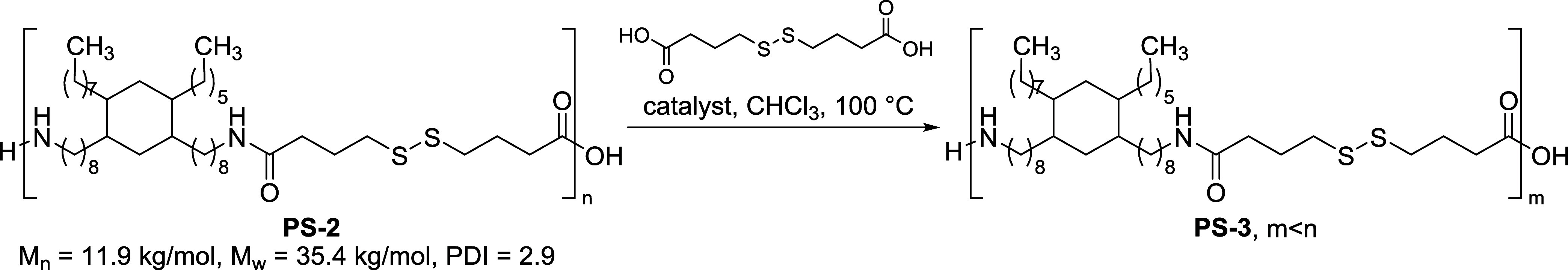
Degradation of Polyamide PS-2 via a Nucleophile-Catalyzed Sulfur–Sulfur Bond Metathesis Reaction^a^

entry	catalyst	mol. %	*M*_n_,^b^ kg/mol	*M*_w_,^b^ kg/mol	PDI^b^
1	DABCO	10	8.9	22.4	2.5
2		50	7.9	18.6	2.4
3		100	9.8	28.4	2.9
4	Cy_3_P	10	12.1	25.0	2.1
5		50	7.3	17.3	2.3
6		100	8.0	20.1	2.5
7	Ph_3_P	10	5.8	14.3	2.5
8		50	3.4	7.3	2.2
9		100	2.4	5.9	2.4

a Reaction conditions: Polymer **PS-2** (50 mg, 0.068 mmol), 4,4′-dithiodibutyric acid (162 mg, 0.68 mmol), and catalyst (10, 50, or 100 mol %) were placed in an 8 mL vial, then chloroform (5 mL) was added, and the reaction mixture was stirred at 100 °C for 19 h. After the reaction was cooled to room temperature, a precipitate was filtered off and the mother liquor was evaporated to dryness. The residue was washed with methanol and dried under vacuum, giving a yellowish elastic material.

b The polymer was analyzed with GPC in THF at 35 °C using polystyrene standards.

Although some degradation of **PS-2** in the presence of the PPh_3_ catalyst is a promising result, the molecular weight of the degradation product was still very high. Hence, other methods to degrade **PS-2** were investigated. These included transition metal-catalyzed,^42^ photocatalytic,^43^ and UV-initiated^40^ disulfide bond metathesis between **PS-2** and diethyl ester of DTDBA (**E-1**) and the photocatalyzed disulfide–ene reaction^43^ (Table 3).

**Table 3:**
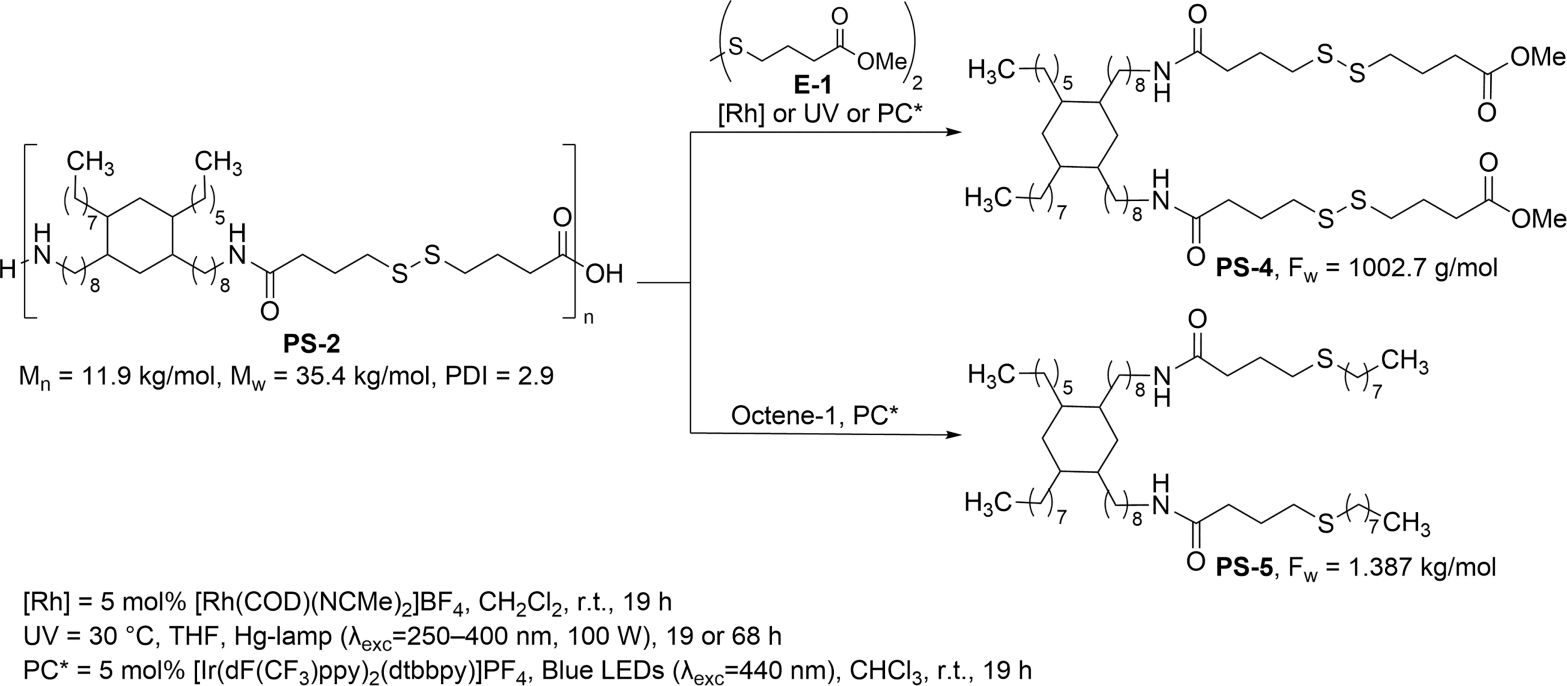
Degradation of Polyamide PS-2 via Sulfur–Sulfur Bond Metathesis Reactions

entry	catalyst	conditions	*M*_n_,^e^ kg/mol	*M*_w_,^e^ kg/mol	PDI^e^
1^a^	5 mol % [Rh(COD)(NCMe)_2_]BF_4_	10 equiv **E-1**, CH_2_Cl_2_, RT, 19 h	5.8	10.9	1.9
2^b^	5 mol % [Ir(dF(CF_3_)ppy)_2_(dtbbpy)]PF_6_	10 equiv E-1, blue LEDs (λ_exc_ = 440 nm), CHCl_3_, RT, 19 h	1.1	1.3	1.1
3^c^	100 W Hg lamp (λ_exc_ = 250–400 nm)	10 equiv **E-1**, THF, 30 °C, 19 h	1.2	1.8	1.4
4^d^	100 W Hg lamp (λ_exc_ = 250–400 nm)	10 equiv **E-1**, THF, 30 °C, 68 h	1.3	1.4	1.1
5^e^	5 mol % [Ir(dF(CF_3_)ppy)_2_(dtbbpy)]PF_6_	10 equiv octene-1, blue LEDs (λ_exc_ = 440 nm), CHCl_3_, RT, 19 h	1.4	3.4	2.5

a Reaction conditions: Polymer **PS-2** (250 mg, 0.34 mmol), dimethyl 4,4′-dithiodibutyrate **E-1** (900 mg, 3.4 mmol) and [Rh­(COD)­(NCMe)_2_]­BF_4_ (6.45 mg, 5 mol %) were placed in an 25 mL ampule, then dichloromethane (10 mL) was added and the reaction mixture was stirred at room temperature for 19 h under argon.

b Reaction conditions: Polymer **PS-2** (250 mg, 0.34 mmol), **E-1** (900 mg, 3.4 mmol), and [Ir­(dF­(CF_3_)­ppy)_2_(dtbbpy)]­PF_6_ (19 mg, 5 mol %) were placed in an 25 mL ampule, then chloroform (10 mL) was added, and the reaction mixture was stirred at room temperature for 19 h under argon under irradiation (λ_exc_ = 440 nm). dF­(CF_3_)­ppy = 2-(2,4-difluorophenyl)-3-trifluoromethylpyridine, dtbbpy = 4,4′-di-tert-butyl-2,2′-bipyridine).

c Reaction conditions: Polymer **PS-2** (50 mg, 0.068 mmol) and **E-1** (181 mg, 0.68 mmol) were placed in an 8 mL vial, then THF (5 mL) was added, and the reaction mixture was stirred at 30 °C for 19 h under argon under irradiation from a 100 W Hg lamp (λ_exc_ = 250–400 nm).

d Reaction conditions: Polymer **PS-2** (1 g, 1.36 mmol) and **E-1** (3.6 g, 13.6 mmol) were placed in a 25 mL ampule, then THF (20 mL) was added, and the reaction mixture was stirred at 25 °C for 68 h under argon under irradiation from a 100 W Hg lamp (λ_exc_ = 250–400 nm).

e Reaction conditions: Polymer **PS-2** (50 mg, 0.068 mmol), octene-1 (76.2 mg, 0.68 mmol), and [Ir­(dF­(CF_3_)­ppy)_2_(dtbbpy)]­PF_6_ (3.8 mg, 5 mol %) were placed in an 8 mL vial, then chloroform (5 mL) was added, and the reaction mixture was stirred at room temperature for 19 h under argon under irradiation (λ_exc_ = 440 nm).

First, a disulfide bond metathesis was tested using 5 mol % [Rh­(COD)­(NCMe)_2_]­BF_4_ at room temperature based on a previous report where this catalyst was used.^42^ In the case of **PS-2** (Table 3, entry 1), the reaction resulted in only a single scission event along the backbone, effectively cleaving the polymer chain into two fragments rather than achieving full degradation. We then moved our attention toward using light as an energy source in the presence of a photocatalyst. This strategy was inspired by previous work where UV and visible light have been used to promote the cleavage of disulfide bonds.^40,43–46^ Interestingly, irradiation of the solution of polyamide **PS-2** and **E-1** in chloroform at 440 nm in the presence of 5 mol % of an iridium photocatalyst [Ir­(dF­(CF_3_)­ppy)_2_(dtbbpy)]­PF_6_ ^47^ led to a significant drop in the molecular weight, giving a product with *M*_n_ = 1.1 kDa (PDI = 1.3) (Table 3, entry 2). A comparable result was obtained using ultraviolet light from a mercury vapor lamp without any catalyst, which gave a product with *M*_n_ = 1.3 kDa (PDI = 1.3) regardless of the reaction time tested (19 or 68 h, Table 3, entries 3 and 4, respectively). Based on previous reports,^48^ we suggest that the degradation of **PS-2** proceeds, as outlined in Scheme 1A. The reaction gets initiated by the homolytic cleavage of the disulfide bond present in diester (**E-1**), forming two thiyl radicals. The reaction of these thiyl radicals with the disulfide bonds present in the polymer chain forms smaller oligomers and **PS-4**.

**Scheme 1:**
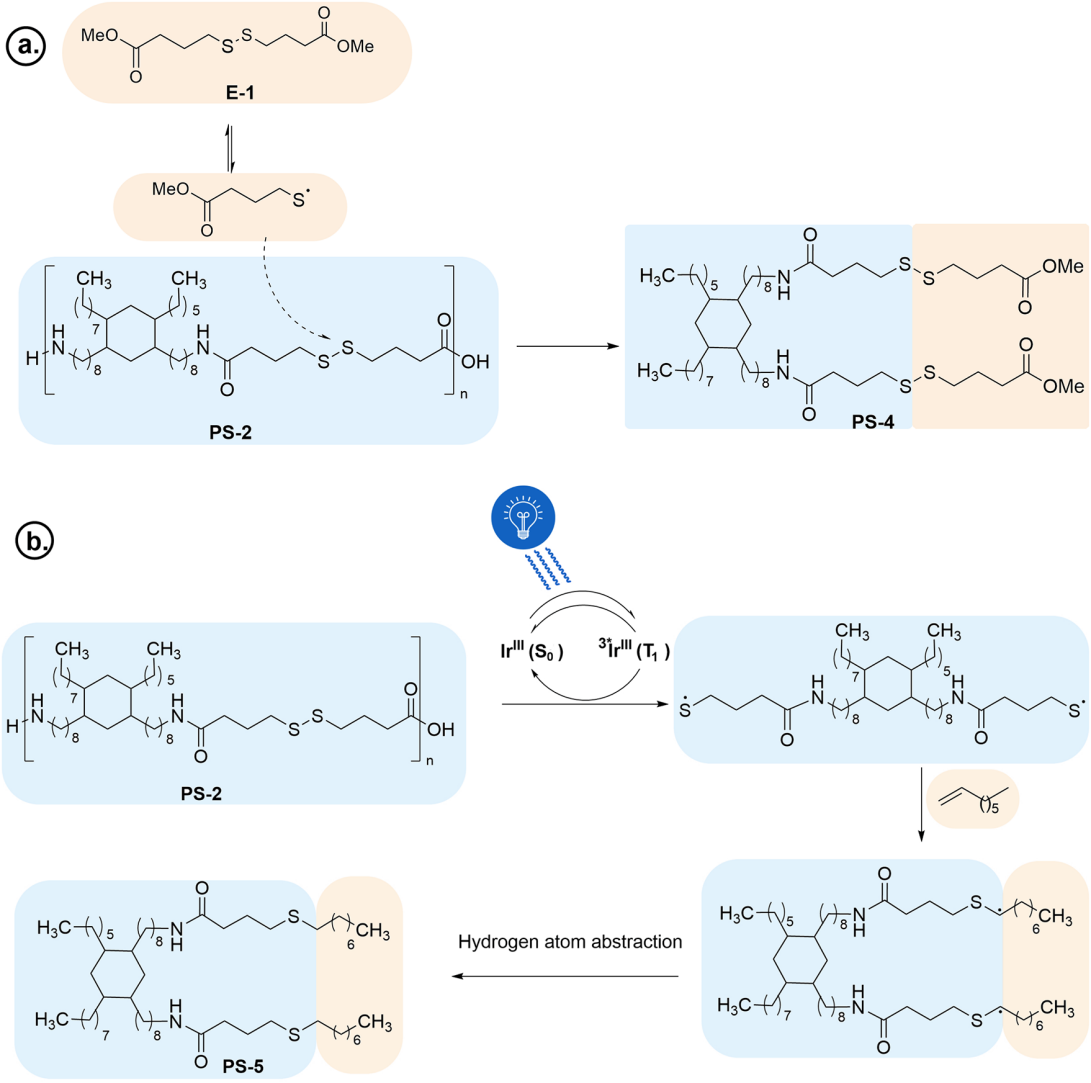
Proposed Pathway for the Degradation of **PS-2** Using (a) Disulfide Bond Metathesis (UV-Initiated), and (b) Thiol–Ene Reaction Using an Iridium Photocatalyst

In another strategy, we utilized a photocatalytic disulfide–ene reaction (λ_exc_ = 440 nm), inspired by a recent report by Glorius and co-workers.^43^ Interestingly, irradiating a mixture of **PS-2** and 1-octene (10 equiv) in the presence of 5 mol % [Ir­(dF­(CF_3_)­ppy)_2_(dtbbpy)]­PF_6_ also produced a low-molecular-weight product *M*_n_ = 1.2 kDa (PDI = 1.2, Table 3, entry 5). We suggest that the degradation process occurs through a mechanism as outlined in Scheme 1B, analogous to that proposed by Glorius.^43^ The reaction starts with the excitation of [Ir­(dF­(CF_3_)­ppy)_2_(dtbbpy)]­PF_6_ photocatalyst, followed by triplet–triplet energy transfer from a visible light photocatalyst [Ir­(dF­(CF_3_)­ppy)_2_(dtbbpy)]­PF_6_ to **PS-2**, leading to the homolytic S–S bond cleavage of the polymer chain, generating oligomers containing thiyl radicals. This is followed by a thiol–ene reaction, forming a carbon-centered radical, which can abstract a hydrogen atom from CHCl_3_, forming **PS-5**.

Hereby, four different reactions were found to be effective in the degradation of polyamide **PS-2** containing a disulfide bond in dicarboxylic acid fragments. The proposed products of these reactions are shown in Table 3. In principle, **PS-4** can potentially be used for the synthesis of polyamides containing disulfide bonds, making the whole process of polymer synthesis and degradation circular.

Considering previous reports on the self-healing properties of polymers containing disulfide bonds,^49–51^ we were interested in assessing whether the **PS-2** polyamide can exhibit self-healing. An ASTM D638 Type IV standard-compliant dog-bone shape tensile specimen of **PS-2** was prepared by dissolving the polymer in NMP (*N*-methyl-2-pyrrolidone) at 80 °C. The solvent from the silicon mold was removed at 50 °C under vacuum, leading to a dog-bone specimen, as shown in Figure 2A. Tensile testing on this sample was performed at room temperature using an Instron 1195 tensile testing machine, equipped with a 50 kN load cell (Figure 2B). The nominal stress–strain curve of virgin **PS-2** (reproduced three times, see ESI, Figure S87 and Table S8) showed an elastic modulus value of 2.12 MPa. 172% elongation-to-failure (ductility) was observed, and a maximum stress value of 1.34 MPa was observed.

**Figure 2:**
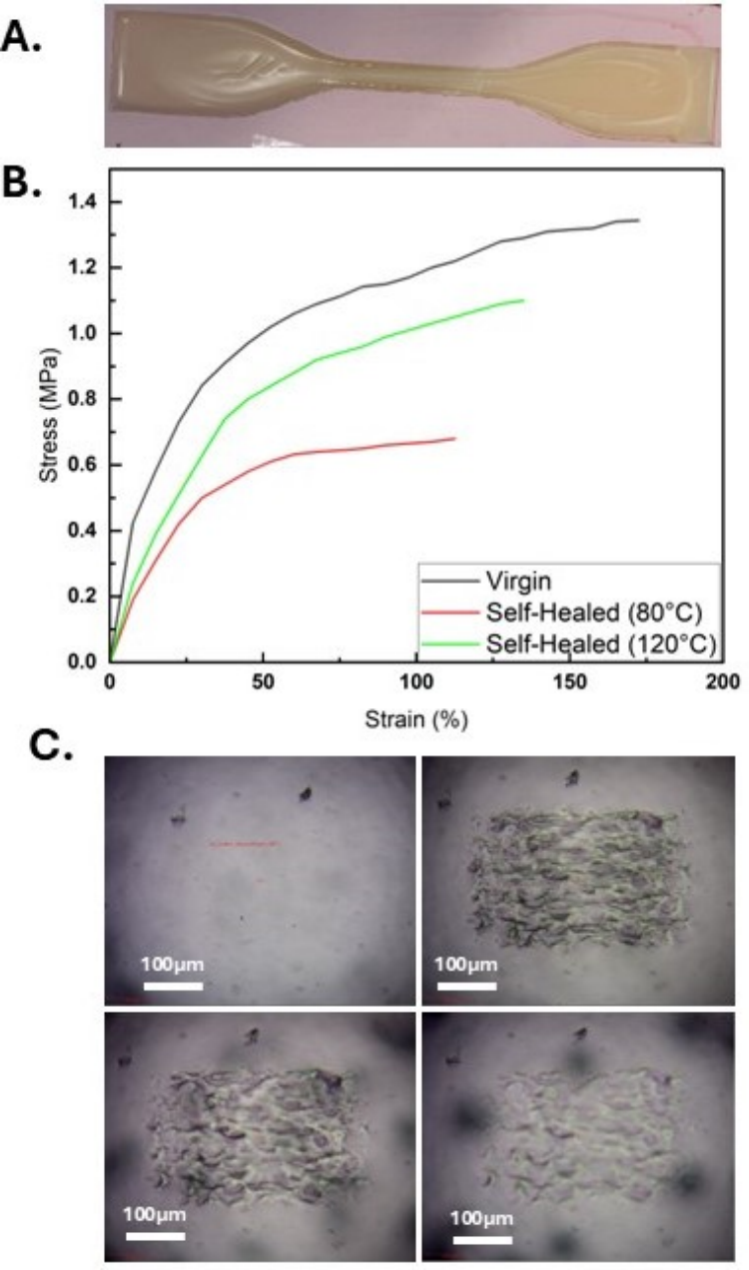
(A) Dog-bone-shaped tensile specimen of **PS-2**. (B) Stress–strain curve of virgin and self-healed **PS-2**. (C) Self-healing study employing microscratch test, followed by predetermined heating cycles observed ex situ under optical microscopy; top left shows the image before scratch, top right shows the image after scratch, the bottom left image is taken after heating at 80 °C for 2 h, and the bottom right image is taken after further heating at 90 °C for 1 h and at 100 °C for 3 h.

For the self-healing study, the dog-bone specimen was cut in half, and afterward, the two edges of the cut sample were brought together by applying gentle pressure and heated at 80 or 120 °C for 30 min. The sample was then cooled to room temperature. This led to the formation of a uniform sample (without any cut) on which tensile testing was performed under the same conditions as those for virgin **PS-2**.

From the stress–strain plot (Figure 2B), the elastic modulus at 80 °C was estimated to be 1.6 MPa, which would suggest 75% recovery upon self-healing (reproduced three times; see ESI, Figure S87 and Table S8). The elongation at break (ductility) and the maximum stress were estimated to be 112% and 0.68 MPa, which would suggest 65 and 51% recovery, respectively. Interestingly, better self-healing was observed at 120 °C, as evidenced by higher elastic modulus (1.9 MPa), elongation at break (135%), and maximum stress (1.1 MPa). These values suggest 78% (from elongation at break perspective) and 82% (from maximum tensile stress) recovery at 120 °C (30 min). Measuring NMR and IR spectra before and after self-healing showed identical signals, confirming that the polymer does not undergo any change in its chemical structure (Figures S90–S94, ESI). Self-healing efficiency here is similar to the reported self-healing nylons in the peer-reviewed literature. For example, Chen and co-workers reported a self-healing efficiency of 80% in 2 h for a nylon elastomer,^26^ whereas Nurhamiyah et al. reported 58–99% self-healing at room temperature–160 °C during 1–48 h.^25^

To gain further understanding of the self-healing properties, a polymer film was prepared through wet-chemical dissolution and doctor-bladed fabrication into a uniformly thick film and subjected to a fine-scale nanoscratch test (Figure S88, ESI). The surface of the polymer film was scratched on a selected area using a KLA iMicro nanoindenter equipped with a Berkovich diamond tip, and the healing was monitored ex situ after heating at different temperatures and specified time intervals under an optical microscope (Figures S88–S91, ESI). The microscopy images (Figure 2C) revealed that although some self-healing occurs at 80 °C, consistent with the stress–strain study (Figure 2B), healing is improved at higher temperatures, such as 90 and 110 °C.

Based on previous reports, it is likely that self-healing properties could be imparted due to the presence of disulfide bonds through dynamic covalent exchange reactions, where disulfide–disulfide metathesis exchange reconnects broken chains after mechanical damage.^52,53^ Their reversibility and relatively low activation energy enable healing, which can be accelerated by heating. We speculate that the long alkyl chains present on the Priamine could also be important in self-healing behavior by introducing flexibility to the material that can enhance the network’s dynamic exchange and fluidity of the sample.^54,55^

Considering previous reports in the literature on the use of sulfur-rich polymers for TENGs (converting mechanical energy to electricity through contact electrification),^56,57^ we envisioned that the self-healing **PS-2** material could be used as a sustainable and resilient triboelectric material when coupled with other commonly used materials, such as Kapton, which is a polyimide, as the counter electrode. The triboelectric output of **PS-2** against Kapton was measured in contact–separation mode (lateral motion), as illustrated in Figures 3A and S97. Upon each contact and separation cycle, charge transfers back and forth between the **PS-2** membrane surface and the Kapton surface and synchronized electron movement through the external circuit, generating sequential positive and negative peaks in the voltage output. Under a consistent impact force of ∼80 N and an oscillation frequency of 2 Hz, the triboelectric pair reached a peak-to-peak open-circuit voltage of 80.7 ± 1.2 V, a short-circuit current of 1.6 ± 0.1 μA, and a charge transfer of 6.4 ± 0.2 nC (Figure 3B–D). The excellent electron-donating ability of **PS-2** in this case is attributed to the rich amide linkages (–CO–NH–) and disulfide bonds (S–S) within the polymer chain, as evidenced by a high surface potential measured under Kelvin probe force microscopy (KPFM, Figure 3E). Indeed, several disulfide-containing polymers have shown enhanced performance for TENGs.^58–61^ This is likely, as polarizable sulfur atoms can increase surface dipole moments, which can strengthen electron transfer during contact electrification. Thus, we demonstrated that **PS-2** is a unique tribopositive material (i.e., an electron donor). Previous studies have also shown the electron-donating nature of other nylon-type polymers for TENGs despite using different combinations of counter materials.

**Figure 3:**
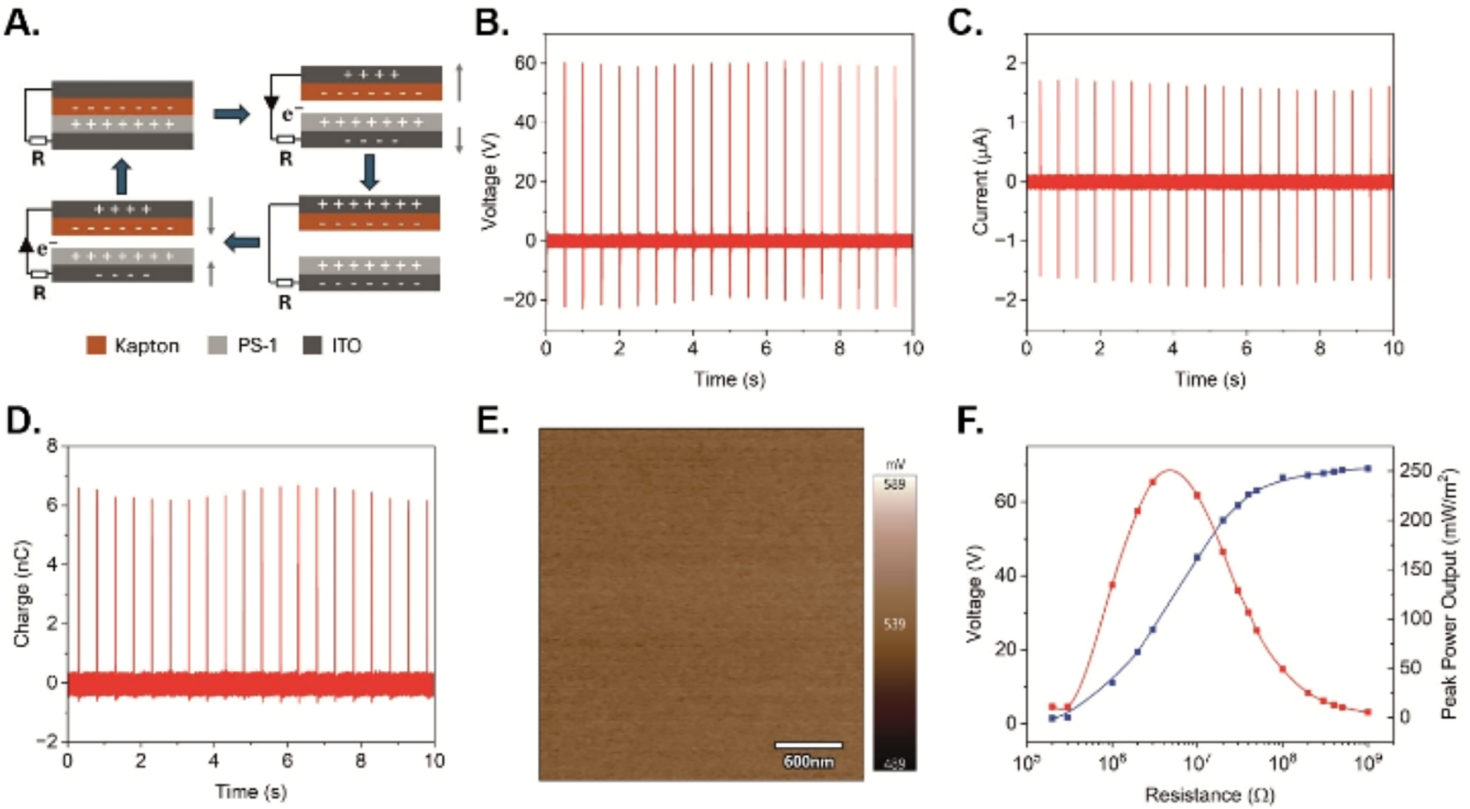
(A) Schematic of the working mechanism of the contact–separation mode TENG. (B–D) Open-circuit voltage, short-circuit current, and charge produced by the **PS-2**-based TENG. (E) KPFM surface potential image of the PS-2 membrane. (F) Voltage and peak power output of the **PS-2** TENG under different load resistances.

To evaluate the performance of the **PS-2**-based TENG device as a power source, the voltage outputs under a series of different load resistances were measured. As depicted in Figure 3F, the voltage increases drastically when the resistance is above 1 MΩ an plateaus when the load resistance is above 100 MΩ. Under a load resistance of 3 MΩ, the TENG device reaches a maximum power output of 239 mW/m^2^. Compared with previously reported tribopositive self-healing TENGs (such as those based on polyesters and polyurethanes), the performance of **PS-2** was found to be above-average (Table S9, ESI).

## Conclusions

Five polyamides containing disulfide bonds were synthesized by using a two-step melt polycondensation reaction. The polymers based on branched Priamine fragments and 4,4′-dithiodibutyric acid, **PS-1** and **PS-2**, are amorphous, with glass transition temperatures of around −13 °C, while their analogues without the disulfide bonds, **PA-1** and **PA-2**, showed more crystalline character, with melting points each near 93 °C. The presence of the disulfide bonds resulted in lower thermal stability compared to sebacic acid-based analogues, with significant degradation occurring at 302 °C for **PS-2** versus 432 °C for **PA-2**.

Various methods for controlled degradation of the disulfide-containing polyamides were investigated. While traditional nucleophilic catalysts like DABCO and Cy_3_P showed limited effectiveness, triphenylphosphine successfully reduced the molecular weight of the polymer. More importantly, photocatalytic methods using both iridium photocatalysts or direct UV irradiation, as well as disulfide–ene and disulfide–thiol photoreactions, proved to be highly effective in degrading the polymer to low-molecular-weight products (*M*_n_ = 1.1–1.5 kDa). The resulting degradation products, particularly **PS-4** can be potentially useful for repolymerization, suggesting a promising circular approach to polymer synthesis and recycling.

This study demonstrates the potential of incorporating disulfide bonds into polyamide structures as a strategy for developing recyclable materials with controlled degradation pathways. The findings offer new perspectives for the design of circular materials.

Further applications of the Priamine-derived polyamides with embedded disulfide bonds **PS-2** in unique self-healing materials and triboelectric nanogenerators have also been demonstrated with promising self-repair features, thermal stability, performance longevity, and utility prospects aimed for practical world applications. The polymer **PS-2** showed up to 82% recovery upon self-healing and a maximum power output of 239 mW/m^2^ under a load resistance of 3 MΩ in the TENG device.

## Supplementary Material



## Data Availability

The research data supporting this publication can be accessed at https://doi.org/10.17630/b07996e5-71c7-4c7d-a9c2-f39fc07172ca.

## Notes

Additional experimental details; materials; and methods, including photographs of the experimental setup, NMR spectra for all compounds, GPC, and FTIR (PDF)
