# Supplementary material for: Semirenewable Polyamides Containing Disulfide Bonds: Synthesis, Degradation, Self-Healing, and Triboelectric Properties
Source: Macromolecules. 2025 Nov 14;58(22):12317–26. doi: 10.1021/acs.macromol.5c02730 (PMC12659427; doi:10.1021/acs.macromol.5c02730)
Supplement: Supplementary file 1 [file ma5c02730_si_001.pdf]

Semi-renewable Polyamides Containing Disulfide Bonds: Synthesis, Degradation, Self-Healing, and Triboelectric Properties

Pavel S. Kulyabin,[†a] Alejandra Sophia Lozano-Pérez,[†a] Tianhuai Xu,[b] Yogeshwar D. More,[b] Harini Sampathkumar,[c] Ketan Pancholi,[c] Oliver Page,[a,d] Chloe Rennie,[a,d] Lea Hämmerling,[a,d] Kelly Lima,[a] Eli Zysman Colman,*[a,d] Jin-Chong Tan,*[b] Amit Kumar*[a]

[a]EaStCHEM, School of Chemistry, University of St Andrews, North Haugh, St Andrews, KY16 9ST, UK.

[b] Multifunctional Materials and Composites (MMC) Laboratory, Department of Engineering Science, University of Oxford, Parks Road, Oxford OX1 3PJ, UK.

[c]The Sir Ian Wood Building, Robert Gordon University, Garthdee Rd, Garthdee, Aberdeen AB10 7GE, U.K.

[d]Organic Semiconductor Centre, EaStCHEM School of Chemistry, University of St Andrews, KY16 9ST, UK.

Table of Contents

Experimental Details 2

1. General Considerations 2
2. Synthesis and Characteristics of Polyamides. 6
   - 2.1 Synthesis of Polyamide from Priamine 1074 and 4,4'-Dithiodibutyric acid (PS-1). 6
   - 2.2 Synthesis of Polyamide from Priamine 1075 and 4,4'-Dithiodibutyric acid (PS-2). 9
   - 2.3 Synthesis of Polyamide from Priamine 1074 and Sebacic acid (PA-1). 12
   - 2.4 Synthesis of Polyamide from Priamine 1075 and Sebacic acid (PA-2). 15
   - 2.5 Synthesis of Polyamide from 1,12-dodecaneamine and 4,4'-Dithiodibutyric acid (PA-3). 18
3. Polyamide PS-2 degradation studies. 21
   - 3.1 1,4-Diazabicyclo[2.2.2]octane DABCO-catalyzed disulfide exchange reaction. 21
   - 3.2 Tricyclohexylphosphine Cy_3P-catalyzed disulfide exchange reaction 24
   - 3.3 Triphenylphosphine Ph_3P-catalyzed disulfide exchange reaction. 28
   - 3.4 Rhodium complex-catalysed disulfide exchange reaction. 32
   - 3.5 Disulfide metathesis under UV-irradiation 35
   - 3.6 Disulfide metathesis under photoirradiation 40
4. Self-healing studies by tensile testing. 50
   - 4.1 Preparation of dogbone specimen. 50
   - 4.2 Self-healing study. 50
   - 4.3 Tensile testing. 50
   - 4.4 Self-healing studies by scratch test (qualitative test) 51
5. Triboelectric Nanogenerator (TENG) Performance of PS-2 Sample. 57
   - 5.1 Fabrication of PS-2/Kapton TENG 57
   - 5.2 TENG Measurements 57

References 61

Experimental Details

1. General Considerations

All manipulations, unless otherwise stated, were performed under an argon atmosphere using standard Schlenk line and glove-box techniques. Glassware were oven-dried and flamed under vacuum prior to use. THF and toluene were dried using a Grubbs-type solvent purification system (Innovative Technologies SPS) equipped with a degasser and were kept over 4 Å molecular sieves under argon. Priamine 1074 and Priamine 1075 were purchased from CRODA Netherlands BV. $[Ir(dF(CF_3)ppy)_2(dtbpy)]PF_6$ was purchased from TCI Chemicals and used as received. Dimethyl 4,4-dithiodibutyrate was synthesised using procedures adapted from literature [1]. All other reagents, including solvents, were purchased from commercial sources and used as received without any further purification.

Solution-state NMR spectra were recorded on a Bruker AVIII 500 MHz or AV 400 MHz NMR spectrometer at 298 K unless otherwise specified. Residual protons of solvent were used as a reference for ^{1}H NMR spectra in deuterated solvent samples. All chemical shifts (δ) are quoted in ppm and coupling constants (*J*) in Hz.

Infrared spectra (ATR-FTIR) were collected using a Shimadzu IRAffinity-1.

Thermogravimetric Analysis (TGA) was performed using Stanton Redcroft STA-780 Series Thermal Analyser between 30–900 °C at a heating rate of 10 °C/min under a flow of nitrogen gas (25 mL/min). Decomposition temperature (T_d, °C) was estimated as the temperature of 5% weight loss.

Differential Scanning Calorimetry (DSC) analyses were performed using a Netzsch DSC204 between -40–300 or 600 °C at a heating rate of 10 °C/min under a flow of nitrogen gas (20 mL/min) after an initial heat/cool cycle (25–300 °C at 10 °C/min) to remove the thermal history of the sample.

For sample preparation for GPC analysis, ~2 mg of a polymer sample was dissolved in ~2 mL of HPLC-grade THF and stirred for ~20 minutes using a magnetic stirrer at room temperature. This led to a homogeneous mixture, which was filtered using a 0.2 µm PTFE syringe filter and immediately injected to the GPC.

Gel permeation chromatography (GPC) was performed on an Agilent 1260 InfinityLab II GPC fitted with a refractive index (RI) detector (35 °C). Two plus guard column Agilent PLgel-M 10 µm MIXED-B columns setup was contained within an oven (35 °C). THF was used as the eluent at a flow rate of 1.0 mL min^{-1}. The calibration was conducted using a series of polystyrene (M_n = 1,000 – 423,000 g mol^{-1}) standards purchased from Agilent Technologies. GC-MS data were collected as solutions in HPLC grade DCM using an Agilent 8860 GC system coupled to an Agilent 5977B EI instrument.

ESI-MS spectra were collected as solutions in acetonitrile using a Micromass LCT spectrometer.

1.1 Characterization of Priamines

1.1.1 Priamine 1074

^{1}H NMR (500 MHz, $CDCl_3$), δ: 2.67 (t, J = 7.0 Hz, 4H), 2.54 (br.s, 1H), 1.55–2.52 (br.s, 4H), 1.01–1.67 (m, 57H), 0.88 (m, 8H).

$^{13}C\{^1H\}$ NMR (125 MHz, $CDCl_3$), δ: 42.3, 34.0, 31.9, 29.7, 26.9, 22.7, 14.1.

HRMS-EI (m/z) calcd for $C_{36}H_{75}N_2^+$ $[M+H]^+$, 535.5925; found, 535.5925

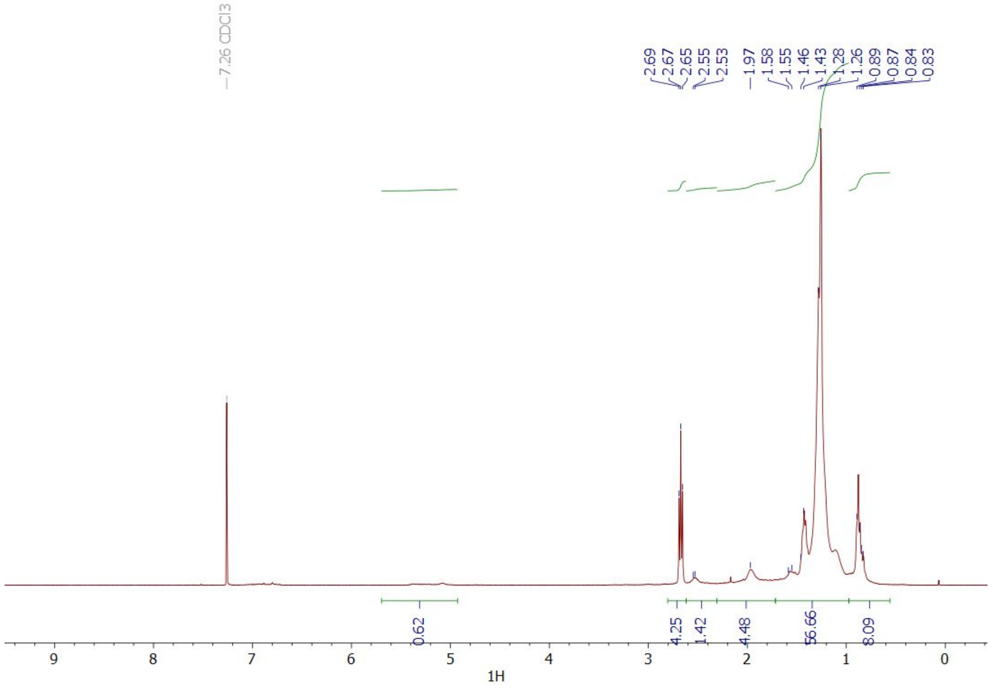

Figure S1. ^{1}H NMR (500 MHz, $CDCl_3$, 298 K) spectrum of polyamide Priamine 1074.

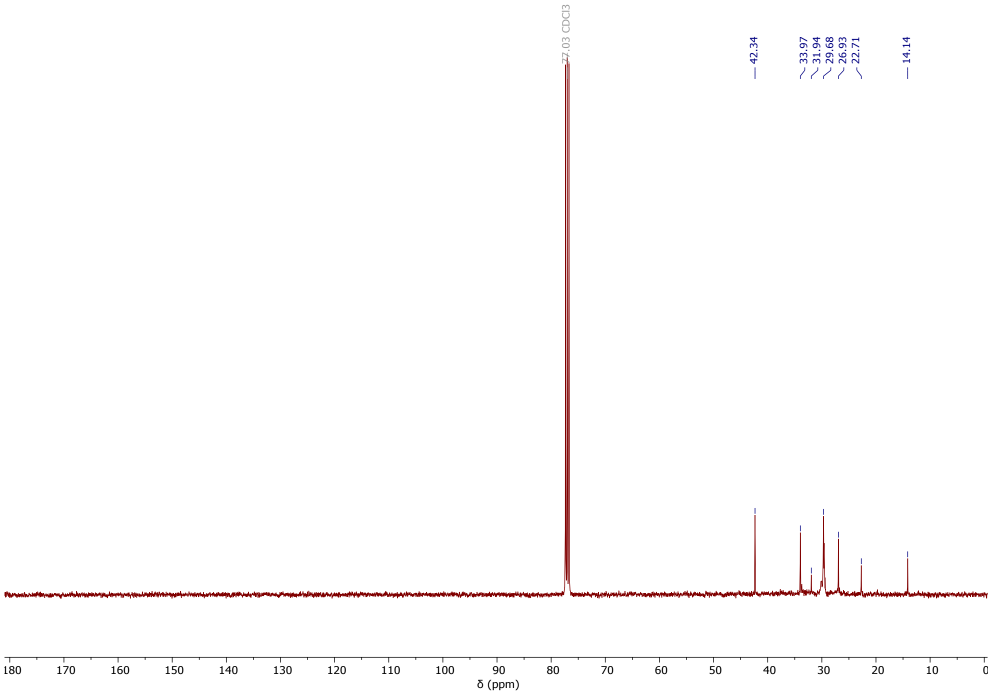

Figure S2. $^{13}C\{^1H\}$ NMR (125 MHz, $CDCl_3$, 298K) spectrum of polyamide Priamine 1074.

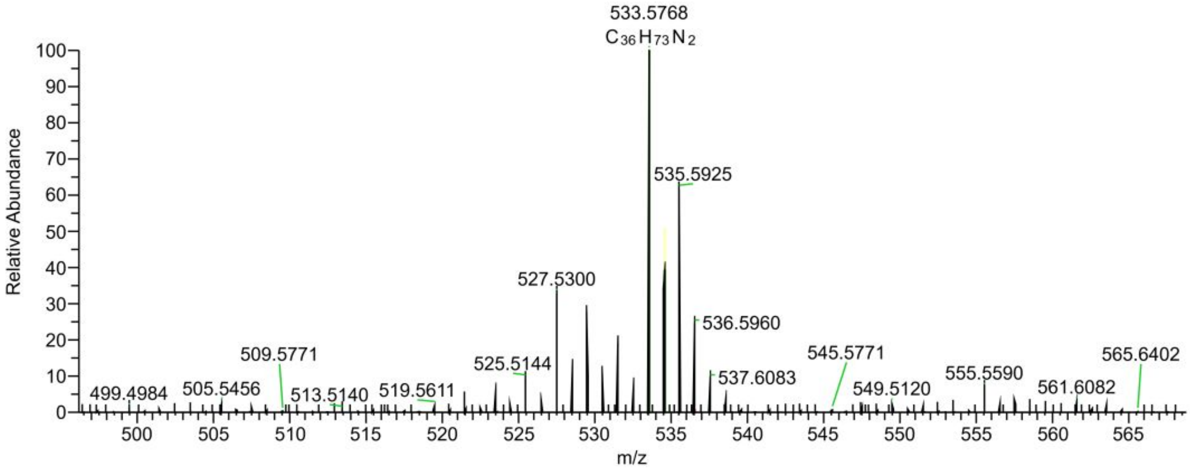

Figure S3. HRMS (ESI) traces of Priamine 1074 solution in methanol.

1.1.2 Priamine 1075

^{1}H NMR (500 MHz, $CDCl_3$), δ: 2.66 (t, J = 7.0 Hz, 4H), 2.54 (br.s, 1H), 1.70–2.50 (m, 2H), 1.07–1.54 (m, 59H), 0.87 (m, 8H).

^{13}C{^{1}H} NMR (125 MHz, $CDCl_3$), δ: 42.5, 34.1, 32.1, 29.8, 29.5, 27.0, 22.8, 14.2.

HRMS-EI (m/z) calcd for $C_{36}H_{75}N_2^+$ [M+H]$^+$, 535.5925; found, 535.5920

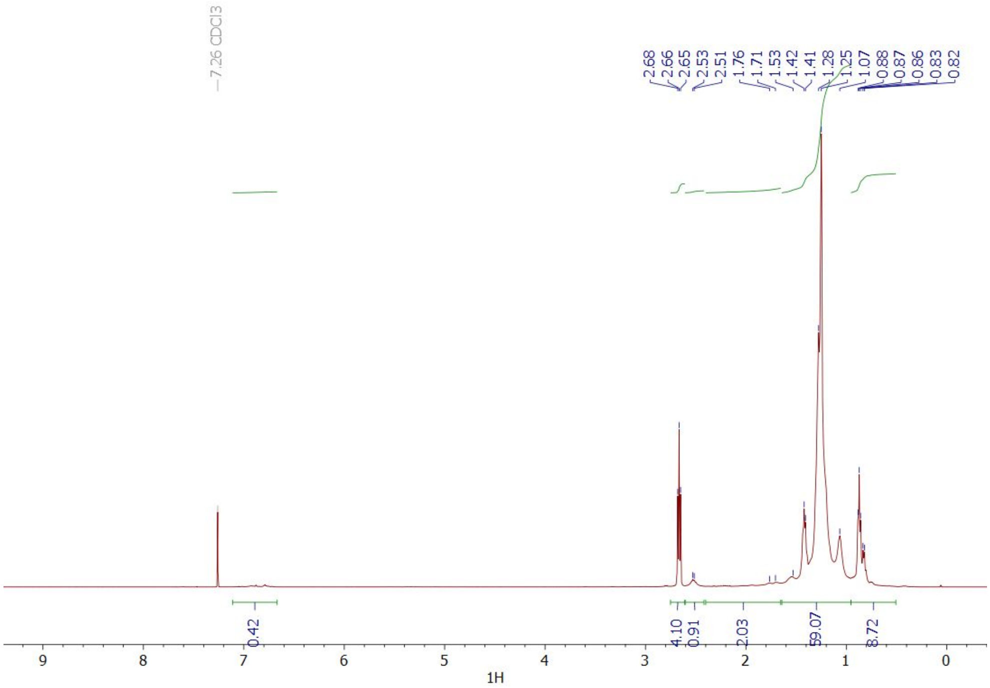

Figure S4. ^{1}H NMR (500 MHz, $CDCl_3$, 298 K) spectrum of polyamide Priamine 1075.

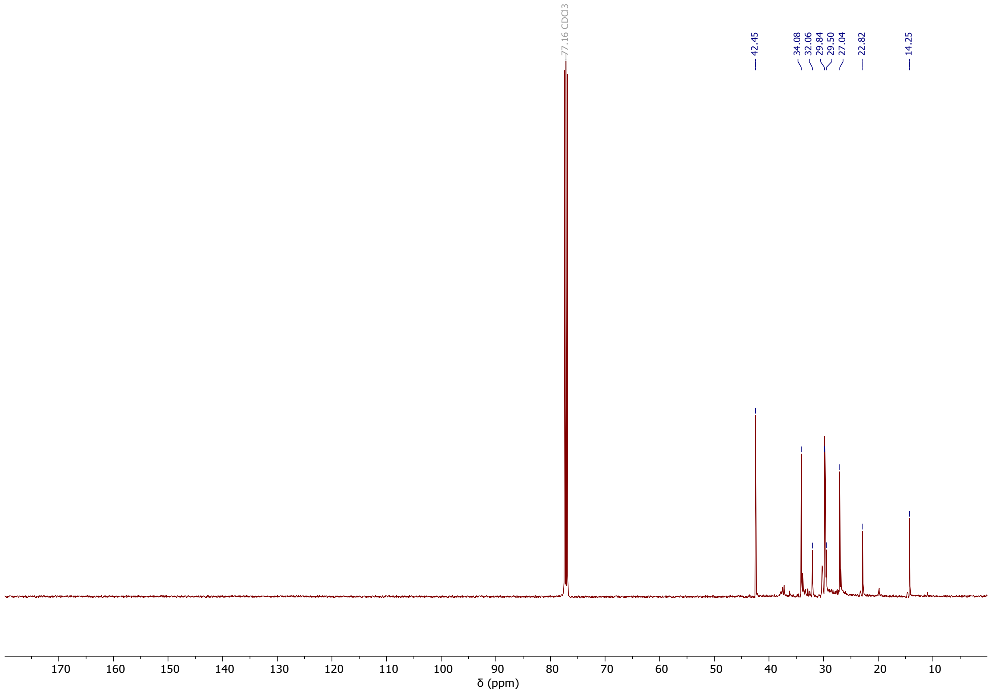

Figure S5. $^{13}C\{^{1}H\}$ NMR (125 MHz, $CDCl_3$, 298K) spectrum of polyamide Priamine 1075.

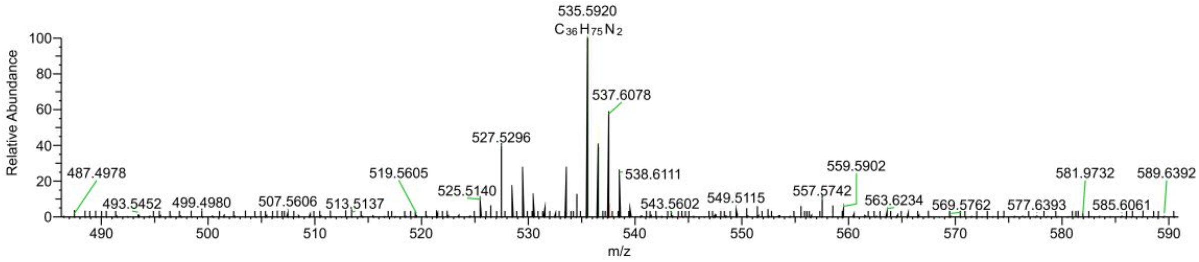

Figure S6. HRMS (ESI) traces of Priamine 1075 solution in methanol.

2. Synthesis and Characteristics of Polyamides.

2.1 Synthesis of Polyamide from Priamine 1074 and 4,4'-Dithiodibutyric acid (PS-1).

4,4′-Dithiodibutyric acid (1.5 g, 6.3 mmol) and Priamine 1074 (3.35 g, 6.3 mmol) were weighed and added into a 50 mL flask equipped with a magnetic stirrer. The flask was refilled with argon, and the reaction temperature was gradually increased to 180 °C and stirred at this temperature for 2 h. Next, the polymerization reaction was continued for 1 h under reduced pressure (1 mbar). The reaction was cooled down to room temperature, then frozen in liquid nitrogen, and the polymer was broken down to remove from the flask. The polymer was collected and dried in vacuum, giving 4.48 g of yellow elastic material (97% yield).

^{1}H NMR (500 MHz, $CDCl_3$), *δ*: 5.56 (br.s, 2H), 3.21 (m, 4H), 2.72 (m, 4H), 2.55 (br.s, 2H), 2.30 (m, 4H), 2.03 (m, 6H), 2.00 (br.s, 2H), 1.01–1.67 (m, 50H), 0.88 (m, 8H).

^{13}C{^{1}H} NMR (125 MHz, $CDCl_3$), *δ*: 172.1, 39.6, 38.2, 34.7, 31.9, 30.2, 29.7, 29.4, 27.0, 26.7, 24.9, 22.7, 14.1.

IR: ν_{max} (ATR), cm^{-1}: 3306w ($\nu_{N\text{-}H}$), 2920s (ν_{CH2}), 2851m (ν_{CH2}), 1638s ($\nu_{C=O}$), 1545m ($\nu_{N\text{-}H}$), 1458w (ν_{CH2}), 1246w, 721w.

GPC (THF): M_n = 5 467, M_w = 13 017 g/mol, PDI = 2.4.

DSC: T_g = -14 °C, T_m = 58 °C.

TGA: T_d (5%) = 307 °C.

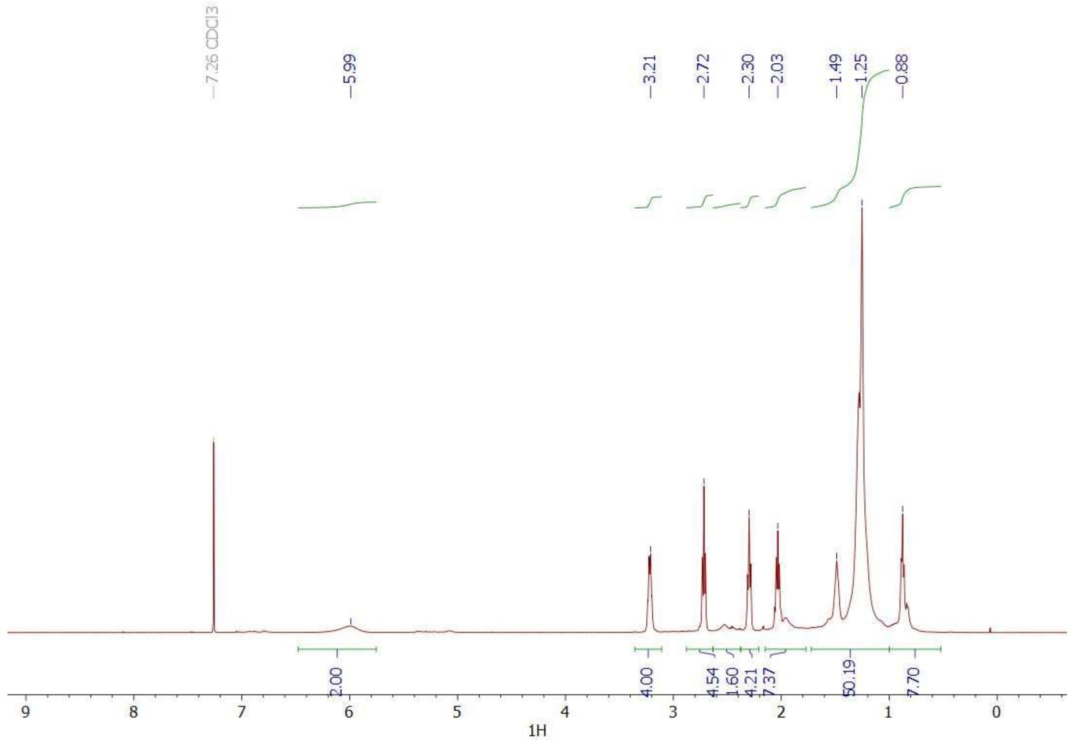

Figure S7. ^{1}H NMR (500 MHz, $CDCl_3$, 298 K) spectrum of polyamide **PS-1**.

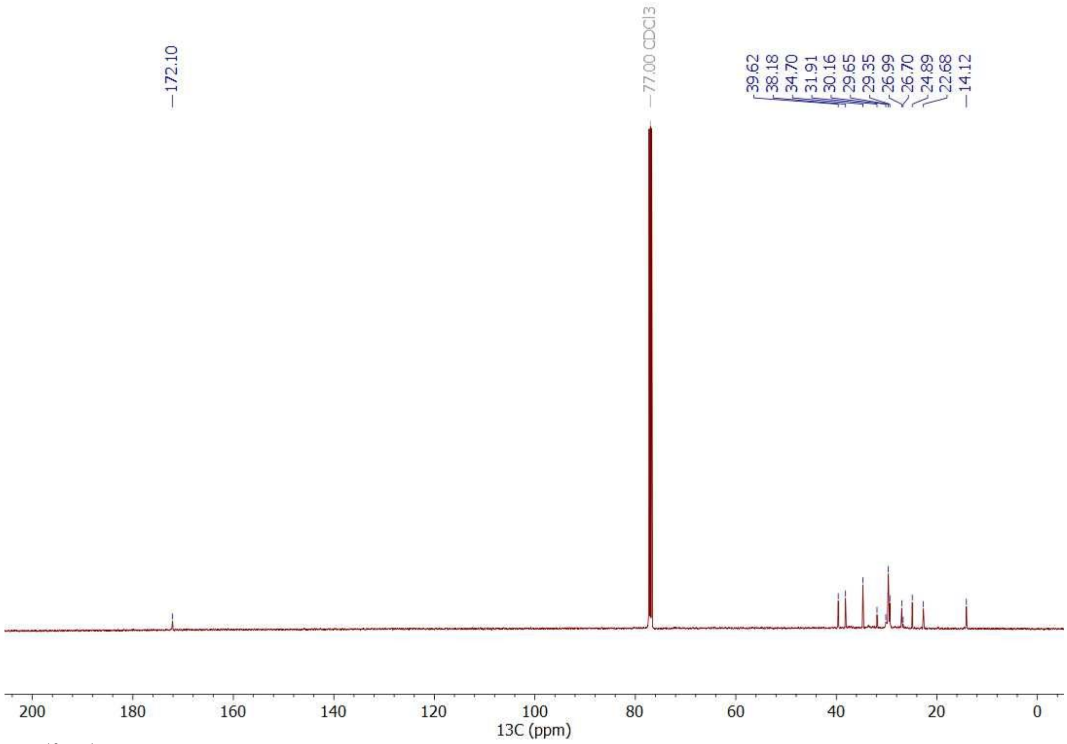

Figure S8. $^{13}C\{^1H\}$ NMR (125 MHz, $CDCl_3$, 298K) spectrum of polyamide **PS-1**.

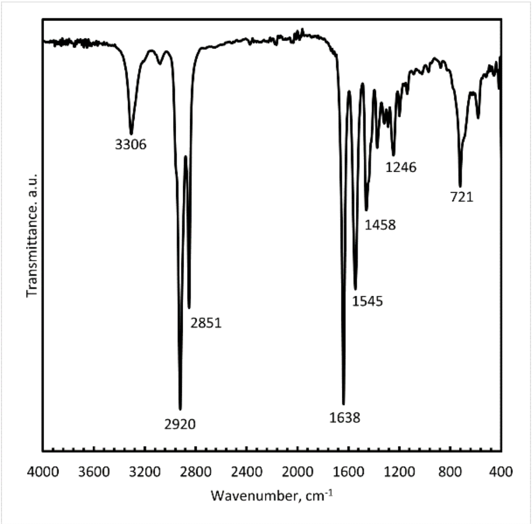

Figure S9. FT-IR (ATR) spectrum of polyamide **PS-1**.

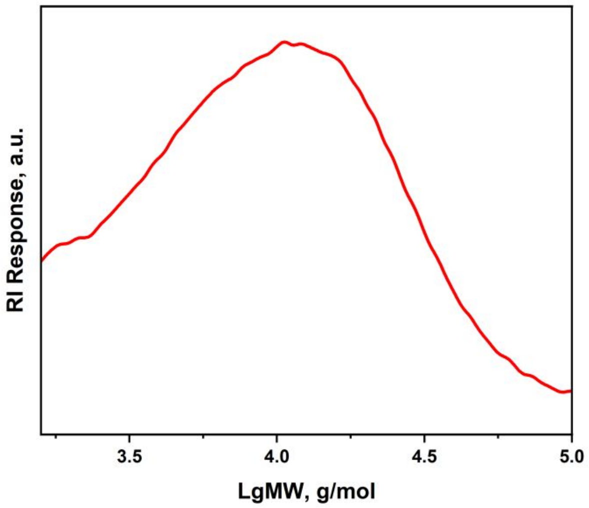

Figure S10. SEC chromatograph corresponding to **PS-1**.

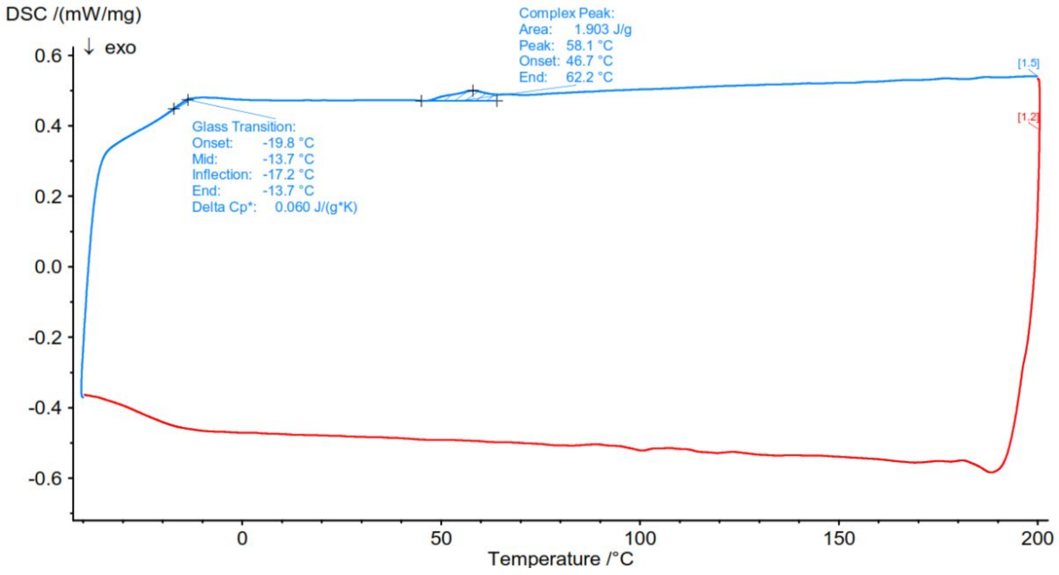

Figure S11. DSC traces corresponding to **PS-1**.

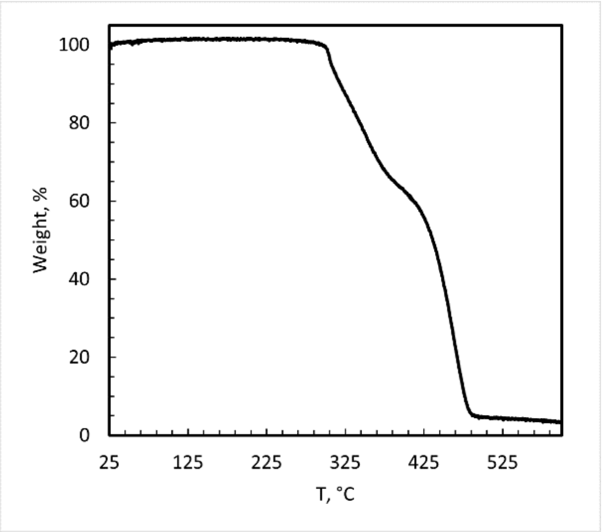

Figure S12. Mass loss as a function of temperature for polyamide **PS-1**.

Table S1. Elemental analysis for polyamide **PS-1**.

**Element**	**Expected (%)**	**Found (1)**	**Found (2)**
Carbon	70.44	68.82	68.28
Hydrogen	12.07	10.30	10.33
Nitrogen	3.50	4.22	4.03
Sulfur	8.00	8.38	8.45
Oxygen	5.99	8.28	8.91

2.2 Synthesis of Polyamide from Priamine 1075 and 4,4'-Dithiodibutyric acid (PS-2).

4,4′-Dithiodibutyric (1.5 g, 6.3 mmol) and Priamine 1075 (3.36 g, 6.3 mmol) were weighed and added into a 50 mL flask equipped with a magnetic stirrer. The flask was refilled with argon and reaction temperature was gradually increased to 180 °C and stirred at this temperature for 2 h. Next, the polymerization reaction was continued for 1 h under reduced pressure (1 mbar). The reaction was cooled down to room temperature, then frozen in liquid nitrogen and the polymer was broken down to remove from the flask. The polymer was collected and dried in vacuum giving 4.64 g of yellow elastic material (98% yield).

^{1}H NMR (500 MHz, $CDCl_3$), δ: 6.00 (br.s, 2H), 3.21 (m, 4H), 2.72 (m, 4H), 2.55 (br.s, 2H), 2.30 (m, 4H), 2.03 (m, 6H), 0.97–1.82 (m, 56H), 0.88 (m, 8H).

^{13}C{^{1}H} NMR (125 MHz, $CDCl_3$), δ: 172.1, 39.6, 38.2, 34.7, 31.9, 30.2, 30.0, 29.7, 29.4, 27.0, 26.7, 24.9, 22.7, 14.1.

IR: ν_{max} (ATR), cm^{-1}: 3302w ($\nu_{N\text{-}H}$), 2920s (ν_{CH2}), 2851m (ν_{CH2}), 1638s ($\nu_{C=O}$), 1545m ($\nu_{N\text{-}H}$), 1458w (ν_{CH2}), 1244w, 721w.

GPC (THF): M_n = 11 953 g/mol, M_w = 35 383 g/mol, PDI = 2.9

DSC: T_g = -12 °C, T_m = 60 °C.

TGA: T_d (5%) = 302 °C.

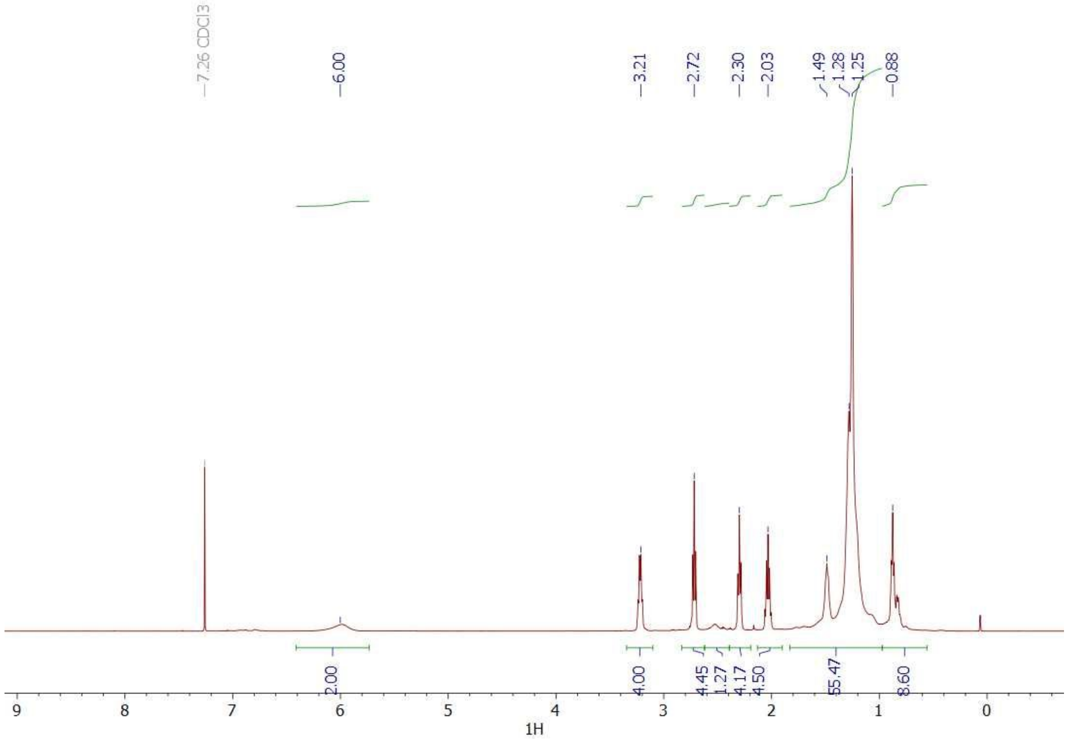

Figure S13. ^{1}H NMR (500 MHz, $CDCl_3$, 298 K) spectrum of polyamide **PS-2**.

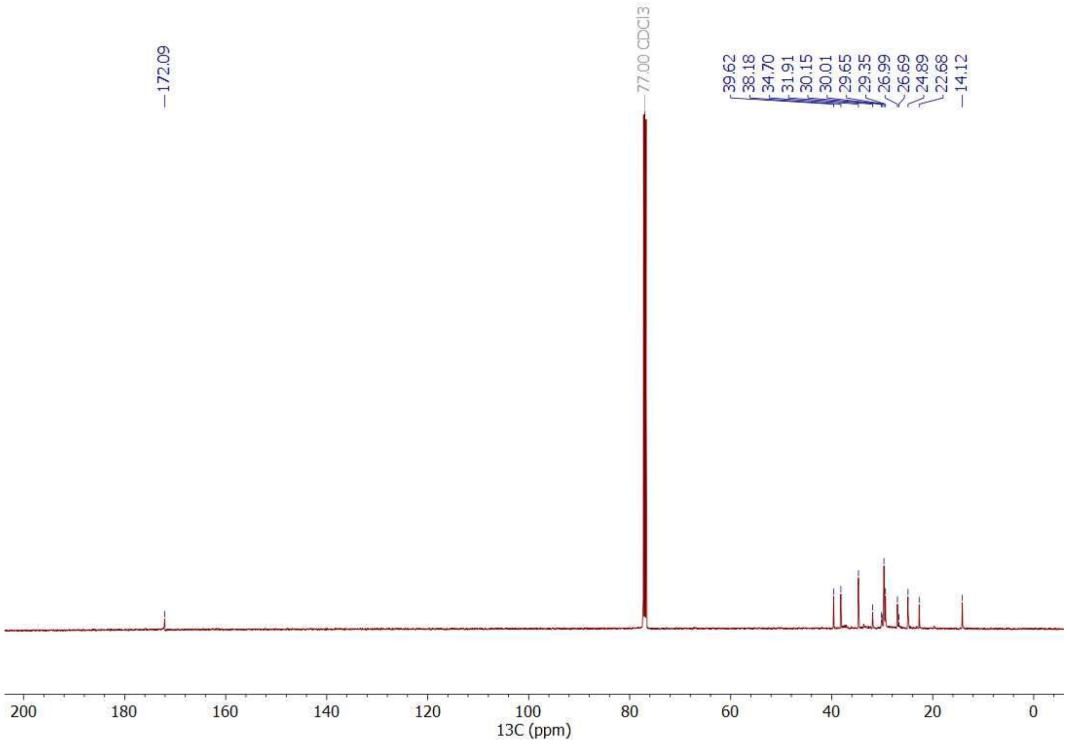

Figure S14. $^{13}C\{^{1}H\}$ NMR (125 MHz, $CDCl_3$, 298 K) spectrum of polyamide **PS-2**.

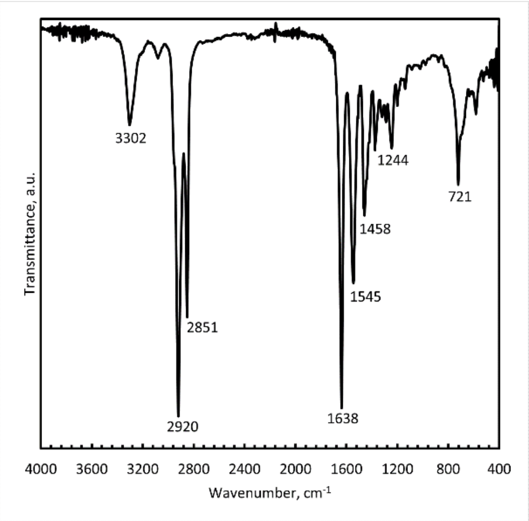

Figure S15. FT-IR (ATR) spectrum of polyamide **PS-2**.

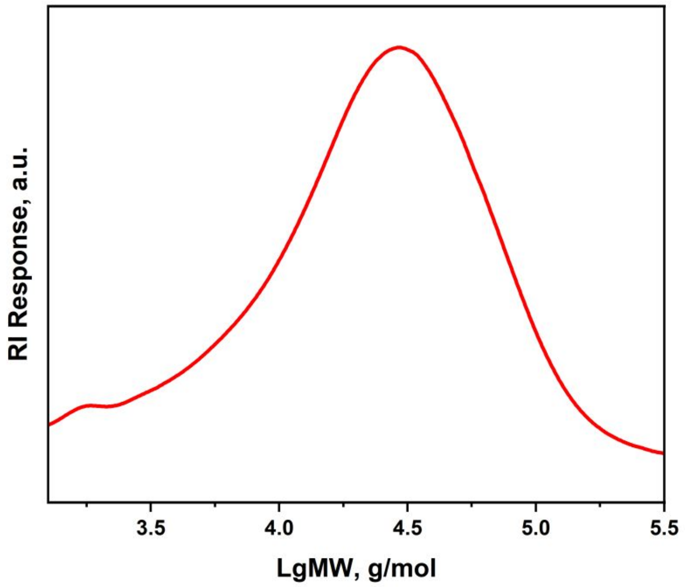

Figure S16. SEC chromatograph corresponding to **PS-2**.

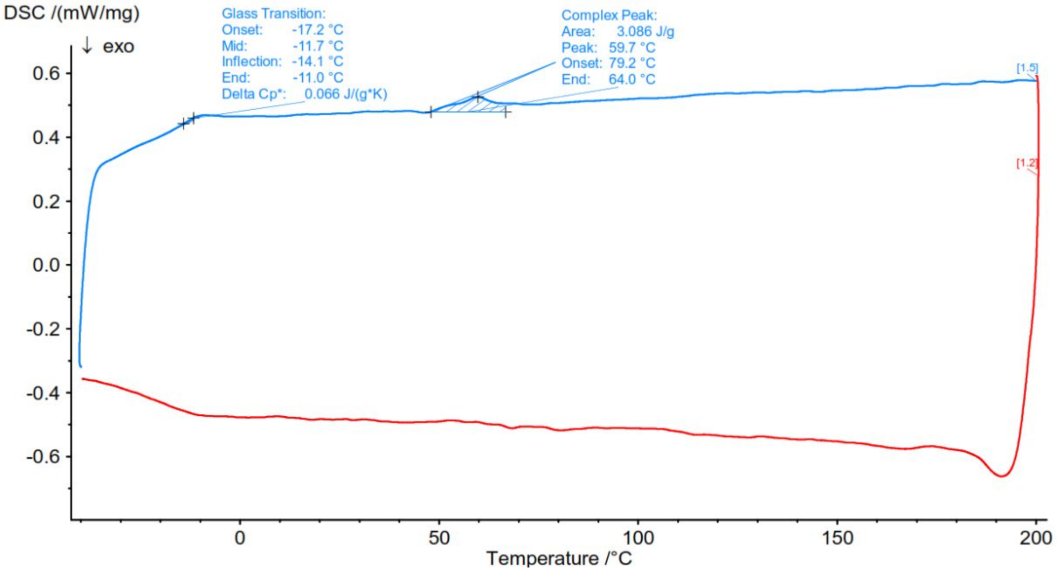

Figure S17. DSC traces corresponding to **PS-2**.

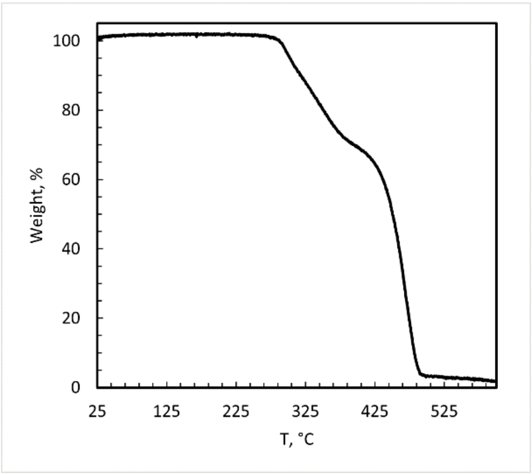

Figure S18. Mass loss as a function of temperature for polyamide **PS-2**.

Table S2. Elemental analysis for polyamide **PS-2**.			
**Element**	**Expected %**	**Found (1) %**	**Found (2) %**
Carbon	70.44	70.73	70.58
Hydrogen	12.07	10.99	10.89
Nitrogen	3.50	4.00	3.85
Sulfur	8.00	6.21	6.24
Oxygen	5.99	8.07	8.44

2.3 Synthesis of Polyamide from Priamine 1074 and Sebacic acid (PA-1).

Sebacic acid (1.5 g, 7.4 mmol) and Priamine 1074 (3.95 g, 7.4 mmol) were weighed and added into a 50 mL flask equipped with a magnetic stirrer. The flask was refilled with argon, and the reaction temperature was gradually increased to 180 °C and stirred at this temperature for 2 h. Next, the polymerization reaction was continued for 1 h under reduced pressure (1 mbar). The reaction was cooled down to room temperature, then frozen in liquid nitrogen, and the polymer was broken down to remove from the flask. The polymer was collected and dried in vacuum, giving 5.06 g of yellowish glassy material (98% yield).

^{1}H NMR (400 MHz, $CDCl_3$), δ: 5.74 (br.s, 2H), 3.21 (m, 4H), 2.52 (m, 1H), 2.14 (m, 4H), 1.96 (br.s, 2H), 1.60 (m, 6H), 1.48 (m, 5H), 1.02–1.41 (m, 49H), 0.87 (m, 7H).

^{13}C{^{1}H} NMR (125 MHz, $CDCl_3$), δ: 173.1, 39.5, 36.8, 31.9, 30.0, 29.7, 29.3, 29.8, 29.1, 27.0, 25.7, 22.7, 14.1.

IR: ν_{max} (ATR), cm^{-1}: 3304w ($\nu_{N\text{-}H}$), 2920s (ν_{CH2}), 2851m (ν_{CH2}), 1638s ($\nu_{C=O}$), 1543m ($\nu_{N\text{-}H}$), 1464w (ν_{CH2}), 1238w, 719w.

GPC (THF): M_n = 10 966 g/mol, M_w = 33 650 g/mol, PDI = 3.02

DSC: T_g = -1 °C, T_m = 92 °C, T_c = 54 °C.

TGA: T_d (5%) = 427 °C.

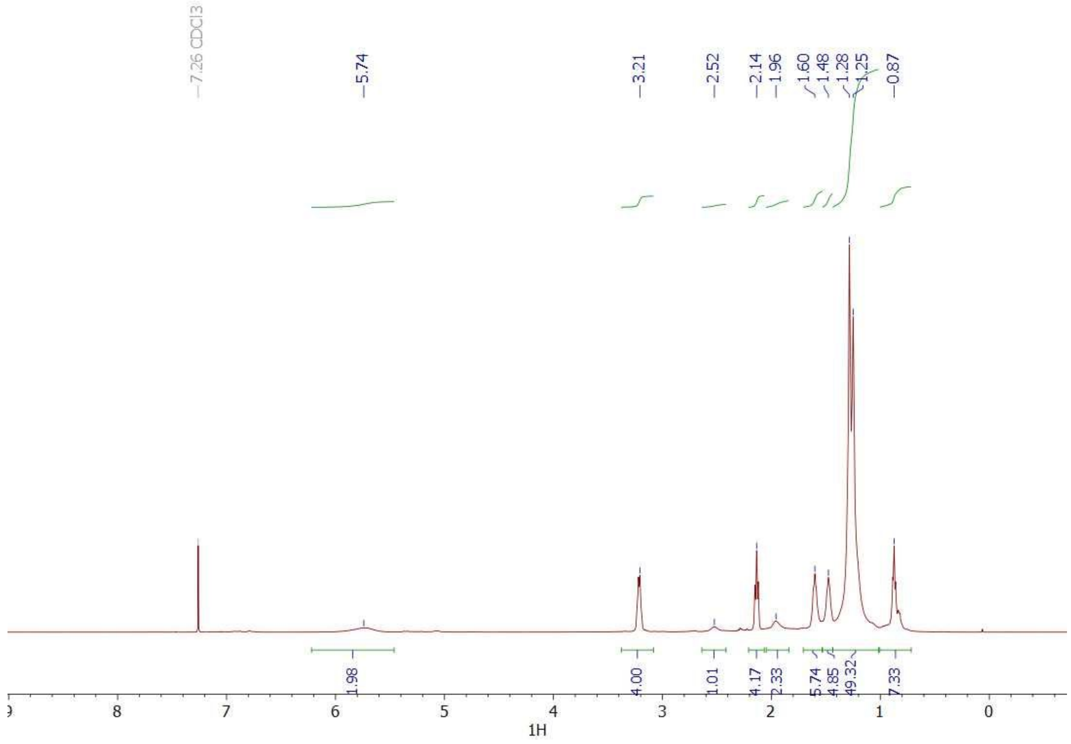

Figure S19. ^{1}H NMR (500 MHz, $CDCl_3$, 298 K) spectrum of polyamide **PA-1**.

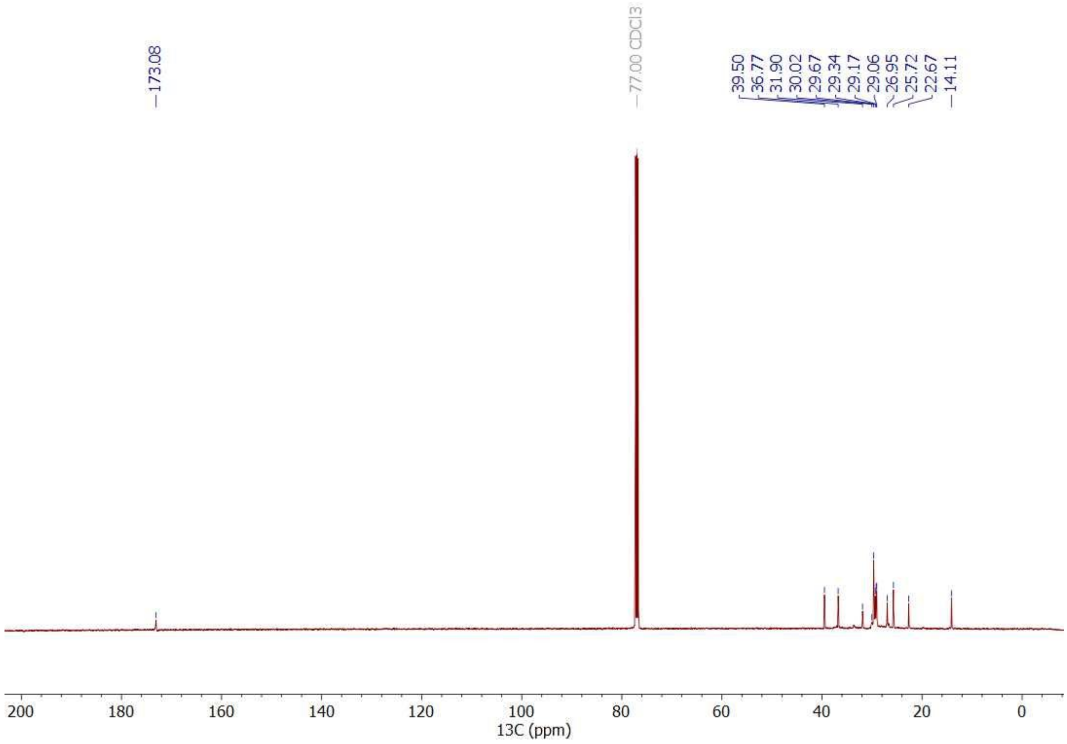

Figure S20. ^{13}C NMR (125 MHz, $CDCl_3$, 298 K) spectrum of polyamide **PA-1**.

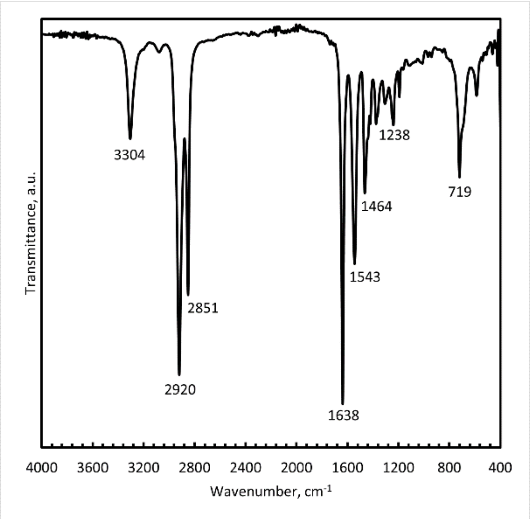

Figure S21. FT-IR (ATR) spectrum of polyamide **PA-1**.

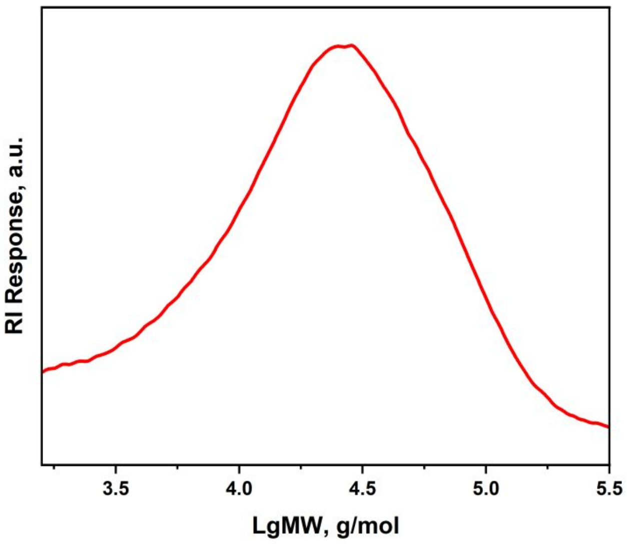

Figure S22. SEC chromatograph corresponding to **PA-1**.

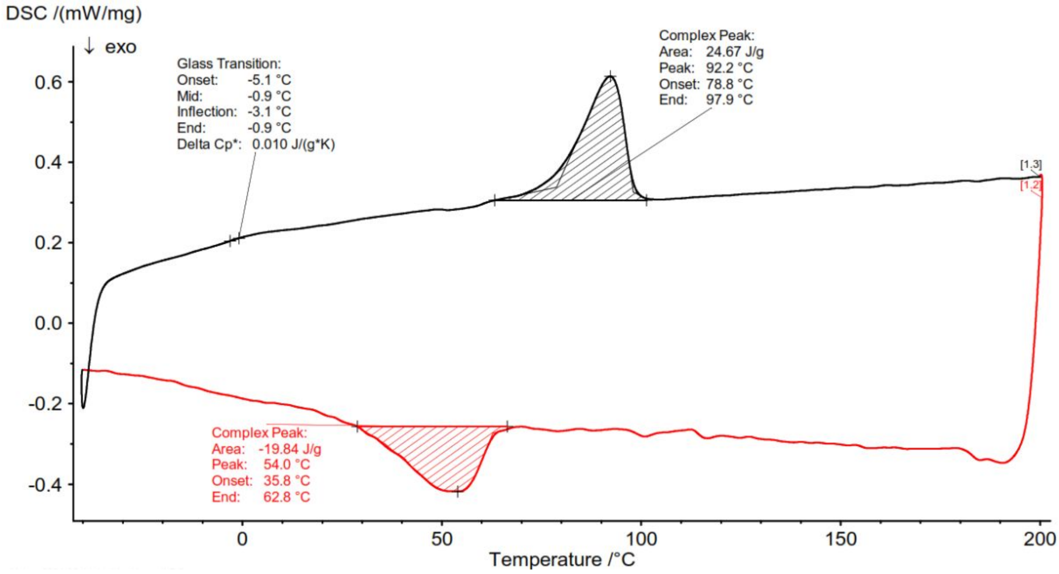

Figure S23. DSC traces corresponding to **PA-1**.

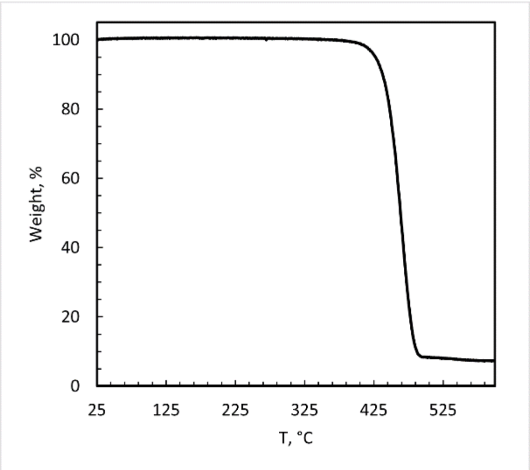

Figure S24. Mass loss as a function of temperature for polyamide **PA-1**.

2.4 Synthesis of Polyamide from Priamine 1075 and Sebacic acid (PA-2).

Sebacic acid (1.5 g, 7.4 mmol) and Priamine 1075 (3.96 g, 7.4 mmol) were weighed and added into a 50 mL flask equipped with a magnetic stirrer. The flask was refilled with argon and reaction temperature was gradually increased to 180 °C and stirred at this temperature for 2 h. Next, the polymerization reaction was continued for 1 h under reduced pressure (1 mbar). The reaction was cooled down to room temperature, then frozen in liquid nitrogen and the polymer was broken down to remove from the flask. The polymer was collected and dried in vacuum giving 5.11 g of yellowish glass (99% yield).

^{1}H NMR (400 MHz, $CDCl_3$), δ: 5.73 (br.s, 2H), 3.21 (m, 4H), 2.52 (m, 1H), 2.14 (m, 4H), 1.42–1.86 (m, 11H), 1.00–1.41 (m, 53H), 0.87 (m, 8H).

^{13}C NMR (125 MHz, $CDCl_3$), δ: 173.1, 39.5, 36.8, 33.7, 31.9, 30.0, 29.7, 29.3, 29.2, 29.1, 27.0, 26.7, 25.7, 22.7, 14.1.

IR: ν_{max} (ATR), cm^{-1}: 3302w ($\nu_{N\text{-}H}$), 2920s (ν_{CH2}), 2851m (ν_{CH2}), 1638s ($\nu_{C=O}$), 1543m ($\nu_{N\text{-}H}$), 1464w (ν_{CH2}), 1238w, 719w.

GPC (THF): M_n = 11 848 g/mol, M_w = 28 893 g/mol, PDI = 2.44.

DSC: T_g = -9 °C, T_m = 94 °C, T_c = 54 °C.

TGA: T_d (5%) = 432 °C

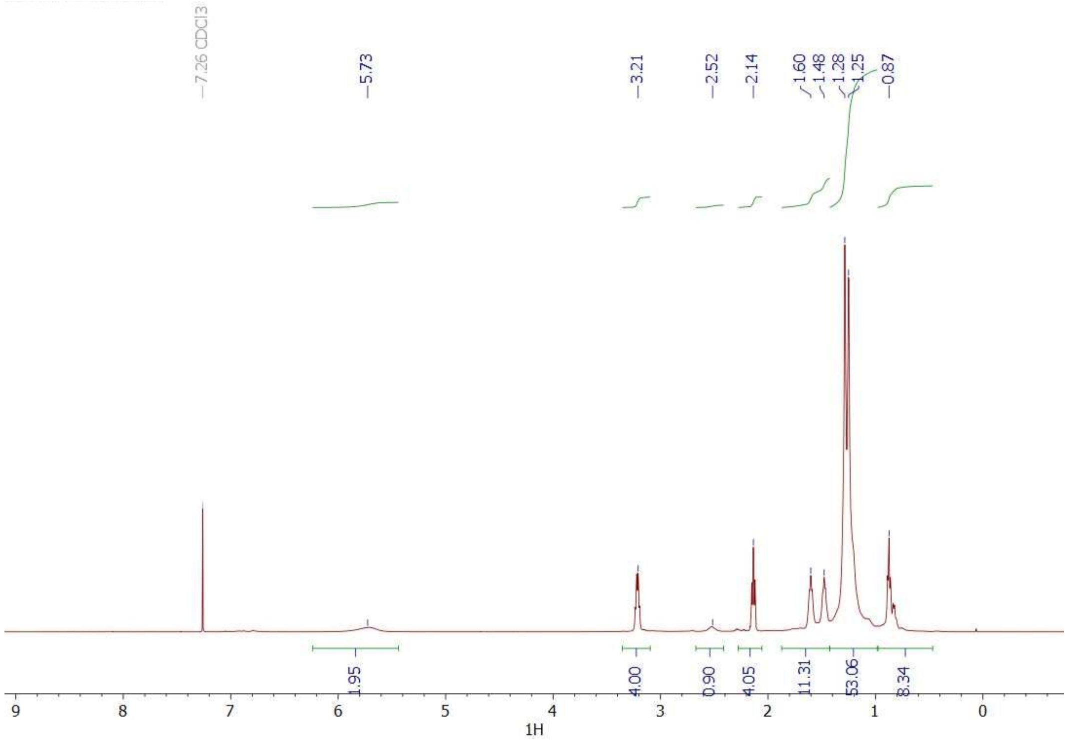

Figure S25. ^{1}H NMR (500 MHz, $CDCl_3$, 298 K) spectrum of polyamide **PA-2**.

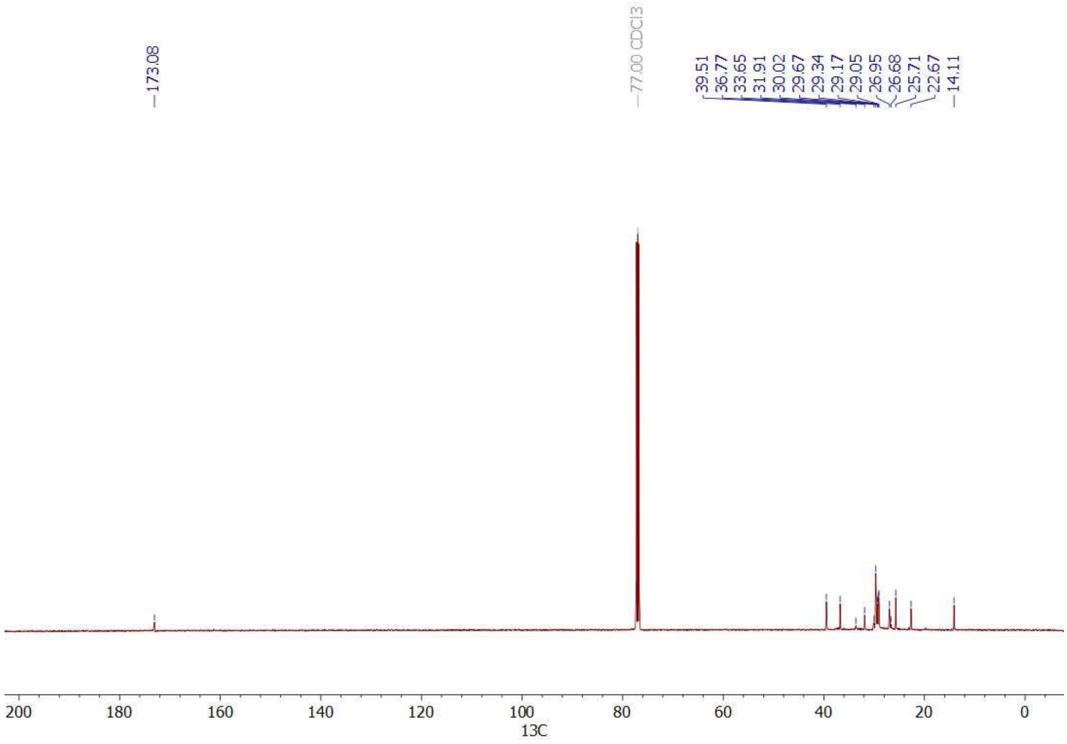

Figure S26. $^{13}C\{^{1}H\}$ NMR (125 MHz, $CDCl_3$, 298 K) spectrum of polyamide **PA-2**.

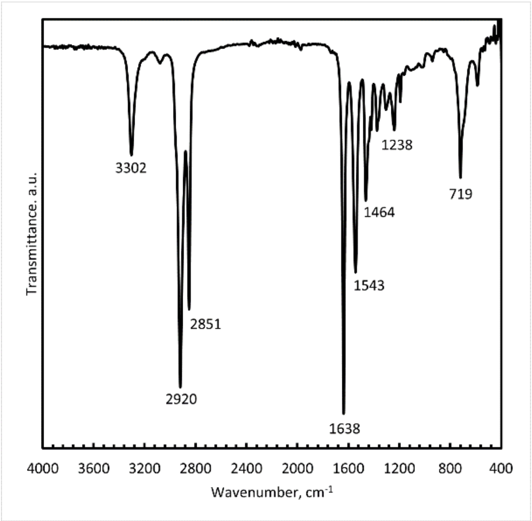

Figure S27. FT-IR (ATR) spectrum of polyamide **PA-2**.

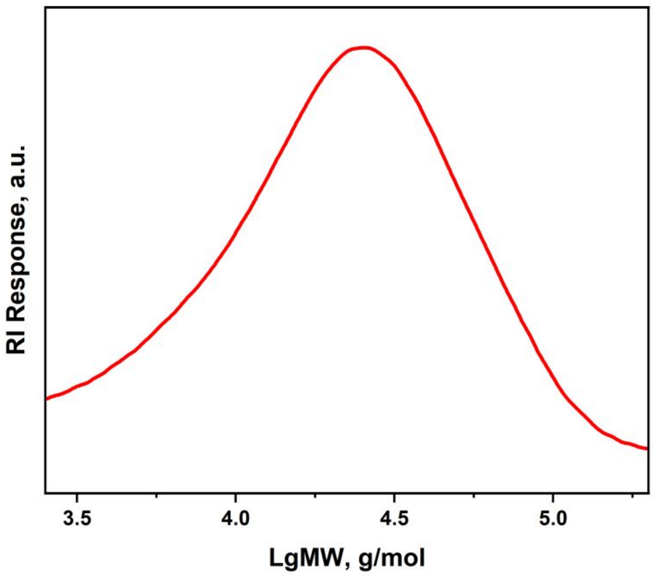

Figure S28. GPC chromatogram of polyamide **PA-2**.

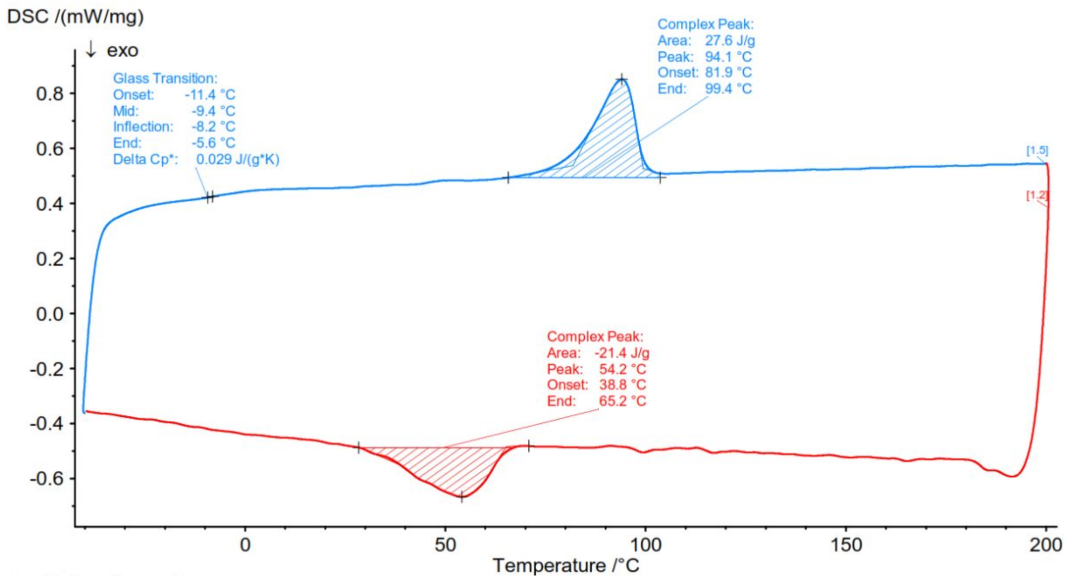

Figure S29. DSC trace corresponding to **PA-2**.

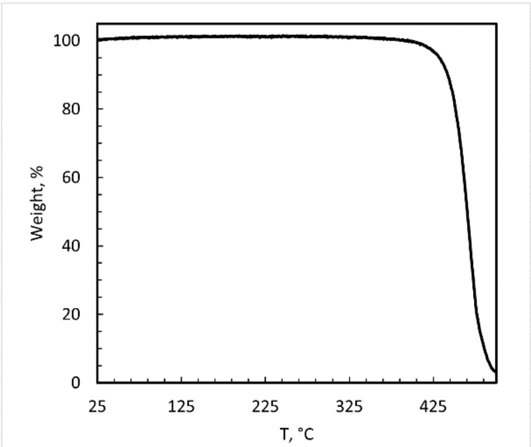

Figure S30. Mass loss as a function of temperature for polyamide **PA-2**.

2.5 Synthesis of Polyamide from 1,12-dodecaneamine and 4,4'-Dithiodibutyric acid (PA-3).

4,4'-Dithiodibutyric acid (1.19 g, 5.0 mmol) and 1,12-dodecaneamine (1.0 g, 5.0 mmol) were weighed and added into a 50 mL flask equipped with a magnetic stirrer. The flask was refilled with argon and reaction temperature was gradually increased to 180 °C and stirred at this temperature for 2 h. Next, the polymerization reaction was continued for 1 h under reduced pressure (1 mbar). The reaction was cooled down to room temperature. The polymer was collected and dried in vacuum giving 1.8 g of white powder (89% yield). NMR sample was prepared in trifluoroacetic acid (TFA) with D_2O capillary.

^{1}H NMR (500 MHz, TFA+D_2O capillary), δ: 3.99 (m, 4H), 3.30 (m, 4H), 3.17 (m, 4H), 2.65 (m, 4H), 2.13 (m, 4H), 1.76 (m, 18H).

^{13}C NMR (125 MHz, TFA+D_2O capillary), δ: 177.5, 42.1, 34.6, 34.5, 31.1, 28.0, 28.0, 27.6, 26.5, 25.2, 23.4, 23.3.

IR: ν_{max} (ATR), cm^{-1}: 3306w (ν_{N-H}), 2920m (ν_{CH2}), 2851m (ν_{CH2}), 1634s ($\nu_{C=O}$), 1533m (ν_{N-H}), 1470w (ν_{CH2}), 1238w, 679w.

GPC (THF): insoluble

DSC: T_m = 163 °C.

TGA: T_d (5%) = 272 °C.

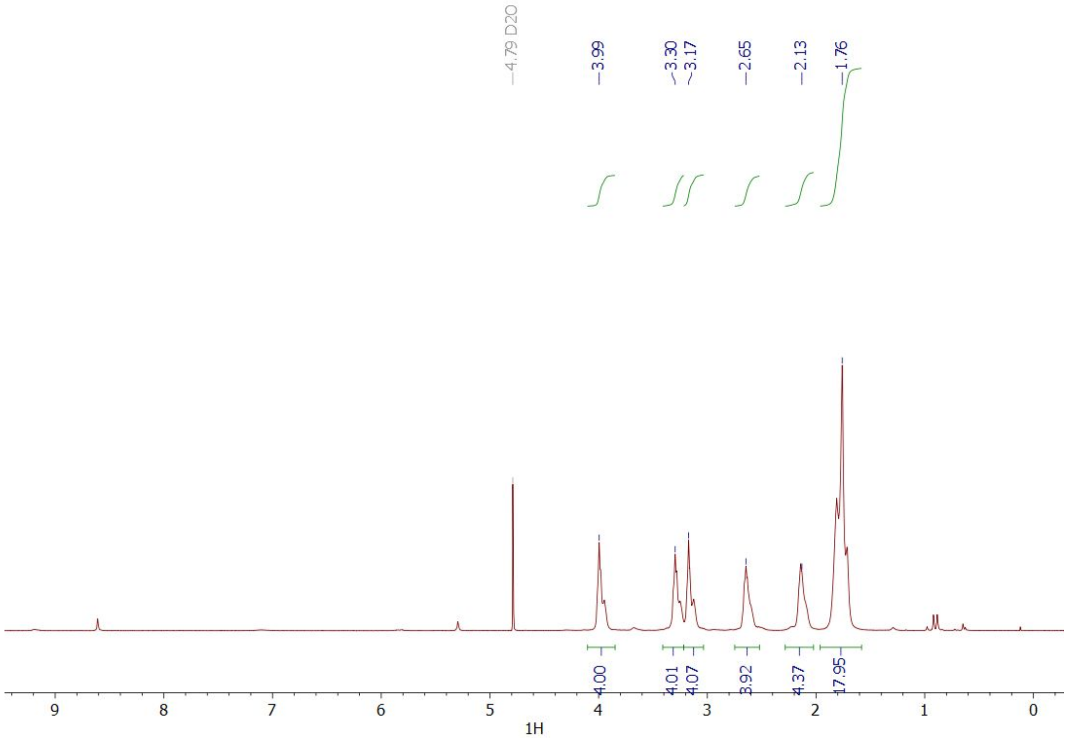

Figure S31. ^{1}H NMR (500 MHz, TFA+D_2O capillary, 298 K) spectrum of polyamide **PA-3**.

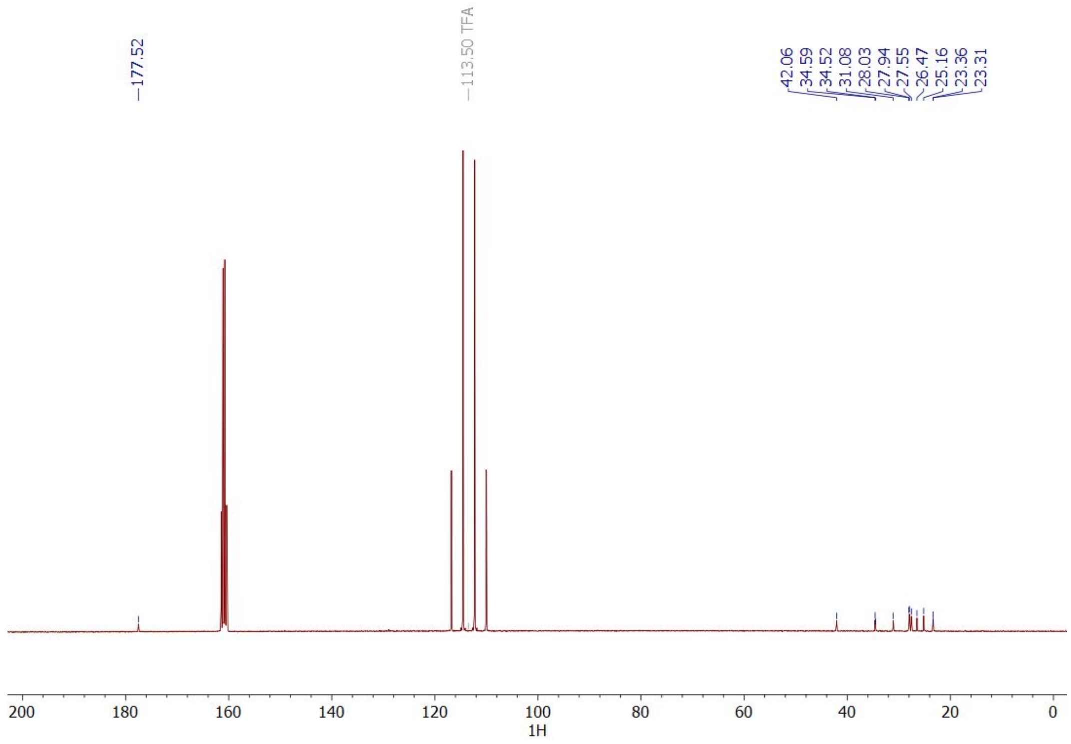

Figure S32. ^{13}C NMR (125 MHz, TFA+D_2O capillary, 298 K) spectrum of polyamide **PA-3**.

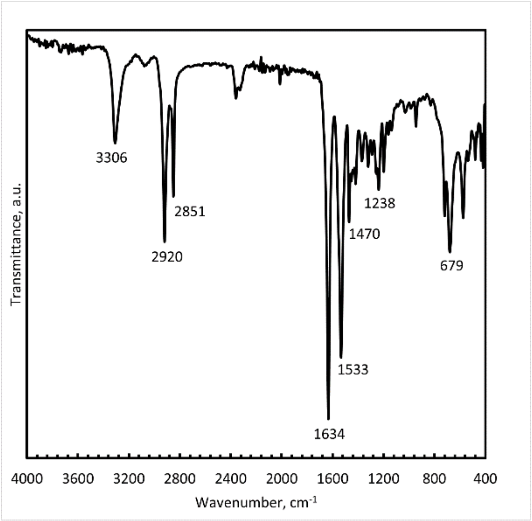

Figure S33. FT-IR (ATR) spectrum of polyamide **PA-3**.

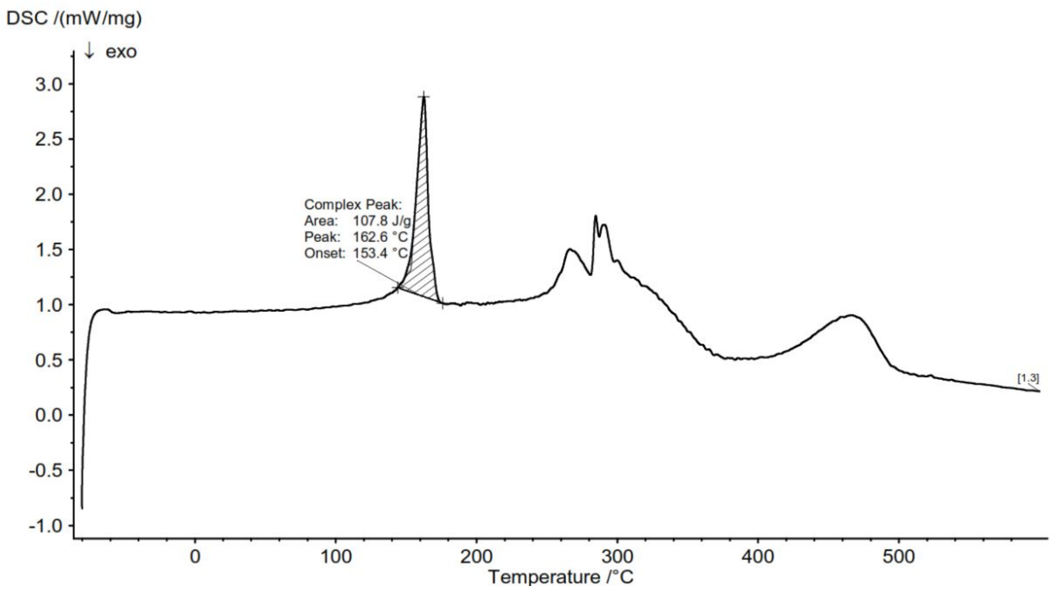

Figure S34. DSC traces corresponding to polyamide **PA-3**.

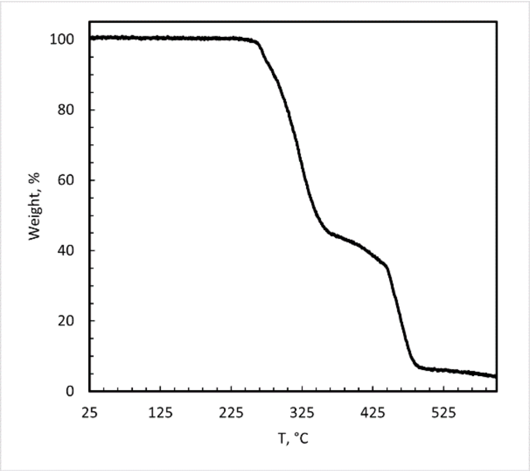

Figure S35. Mass loss as a function of temperature for polyamide **PA-3**.

3. Polyamide PS-2 degradation studies.

3.1 1,4-Diazabicyclo[2.2.2]octane DABCO-catalyzed disulfide exchange reaction.

Polymer PS-2 (50 mg, 0.068 mmol) and 4,4′-Dithiodibutyric acid (162 mg, 0.68 mmol) and DABCO (**Entry 1, Table S1:** 1.53 mg, 10% mol, **Entry 2, Table S1:** 7.62 mg, 50% mol, **Entry 3, Table S1:** 15.26 mg, 100% mol) were placed in a 8 mL vial, then chloroform (5 mL) was added and the reaction mixture was stirred at 100 °C for 19 h. After the reaction was cooled down to room temperature, the formation of a precipitate was observed. The precipitate was filtered off and washed with chloroform. The mother liquor was evaporated and washed with methanol and diethyl ether and dried in vacuum, leaving a yellowish residue. The residue was dissolved in dichloromethane (30 mL) and washed with 1 M solution of hydrochloric acid and then – 1 M solution of sodium hydroxide (30 mL). Organic layer was collected, dried over sodium sulphate and the solvent was evaporated. The residue was dried on vacuum.

Table S3. Degradation of Polyamide PS-2 via DABCO-catalyzed sulfur-sulfur bond metathesis reaction.[a]

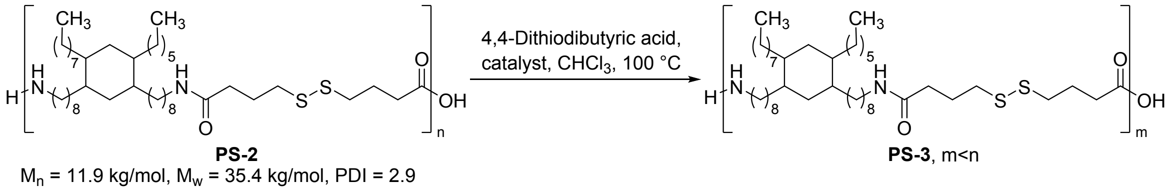

Entry	Catalyst	Mol. %	Isolated material, mg	M_n,[b] kg/mol	M_w,[b] kg/mol	PDI[b]
1		10	50	8.9	22.4	2.5
2	DABCO	50	46	7.9	18.6	2.4
3		100	48	9.8	28.4	2.9

[a] The polymer was analysed with GPC in THF at 35 °C relative to polystyrene standards.

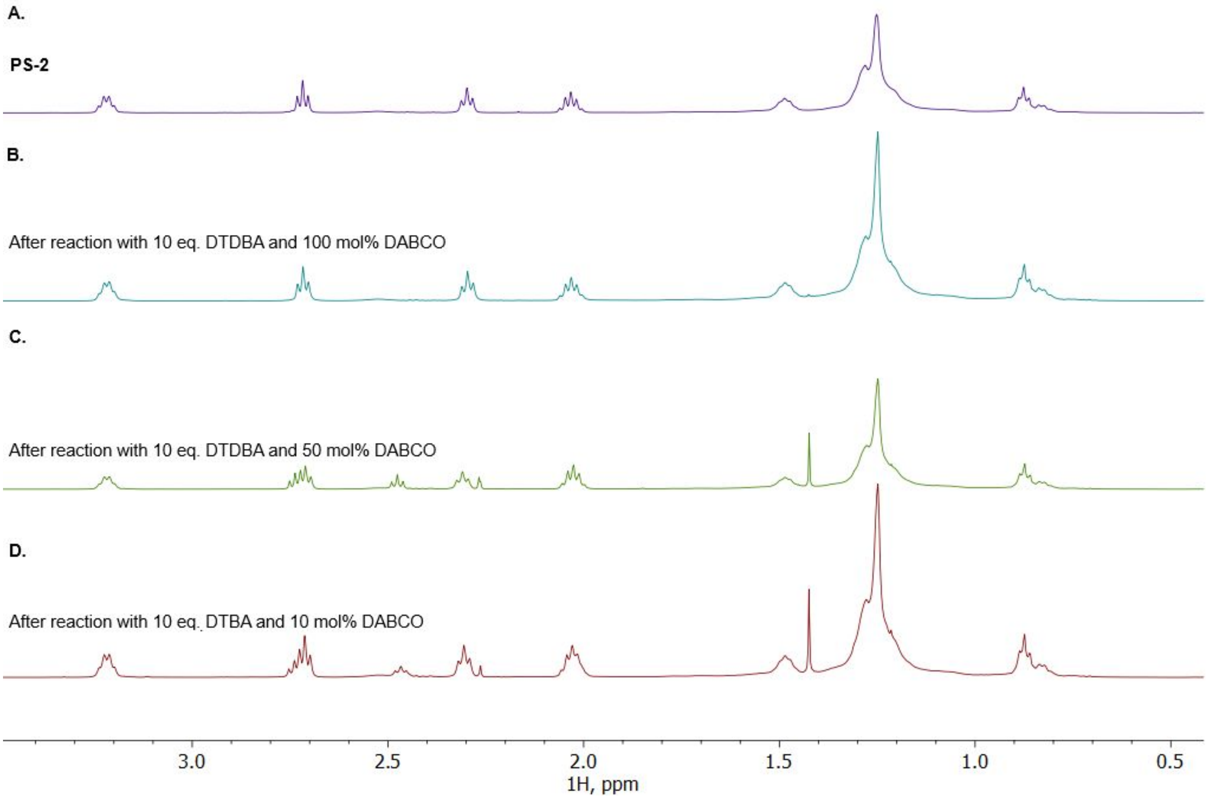

Figure S36. Fragments of ^{1}H (500 MHz, $CDCl_3$, 298 K) NMR spectra of **PS-2** and its reactions with DTDBA catalysed by DABCO. In spectra **C** and **D** signals of DTDBA residue can also be seen.

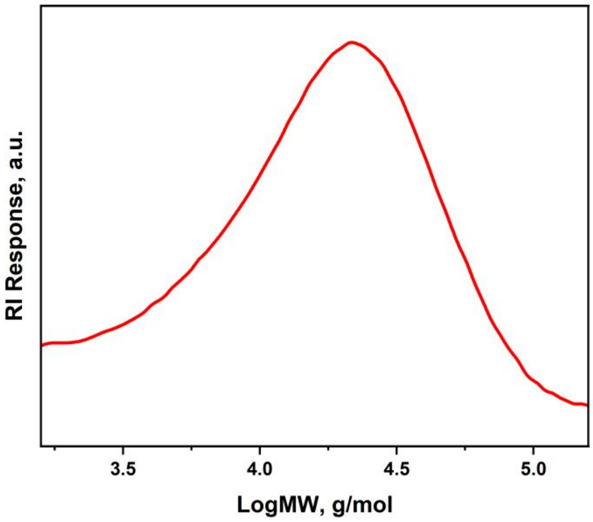

Figure S37. GPC data of the sample from entry 1, Table S1 after reaction of PS-2 with 4,4'-dithiodibutyric acid catalysed by 10 mol% DABCO.

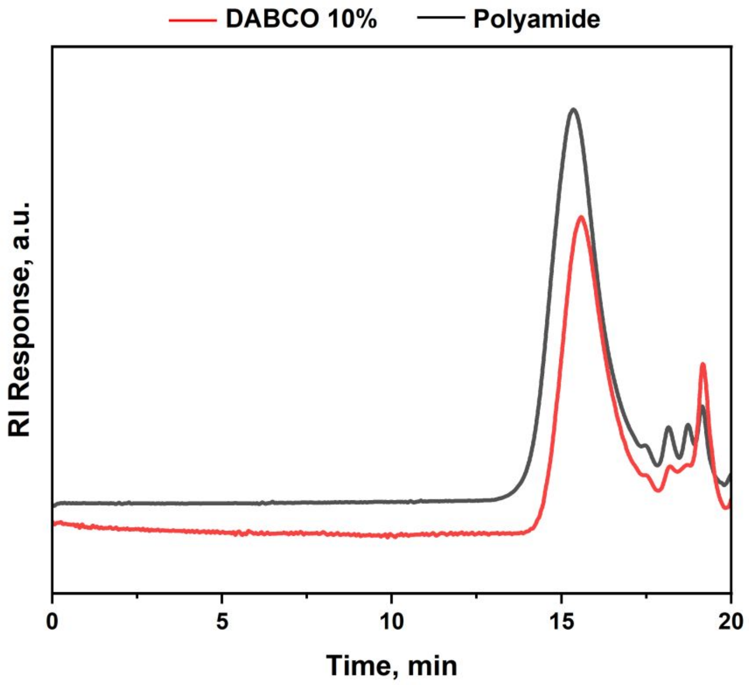

Figure S38. GPC data of the sample from entry 1, Table S1 after reaction of PS-2 with 4,4'-dithiodibutyric acid catalysed by 10 mol% DABCO (red) in comparision with virgin polymer PS-2 (black).

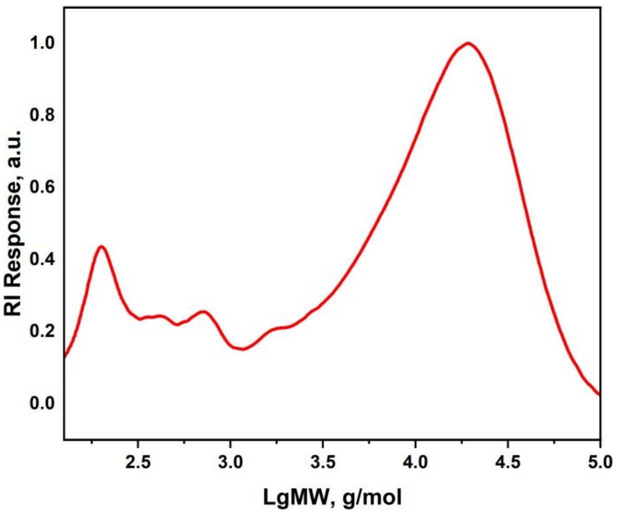

Figure S39. GPC data of the sample from entry 2, Table S1 after reaction of PS-2 with 4,4'-dithiodibutyric acid catalysed by 50 mol% DABCO.

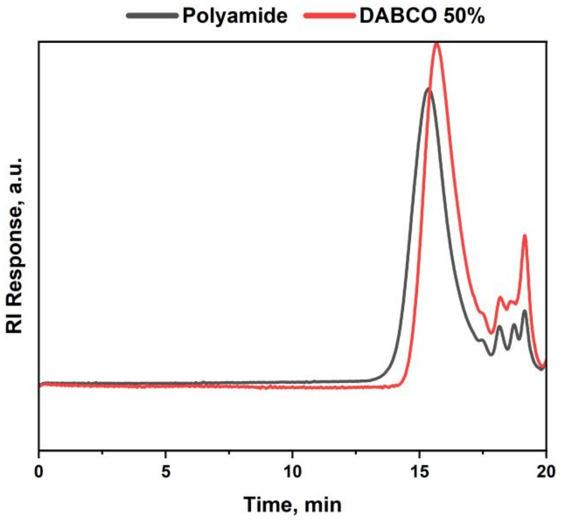

Figure S40. GPC data of the sample from entry 2, Table S1 after reaction of PS-2 with 4,4'-dithiodibutyric acid catalysed by 50 mol% DABCO (red) in comparision to virgin polymer PS-2 (black).

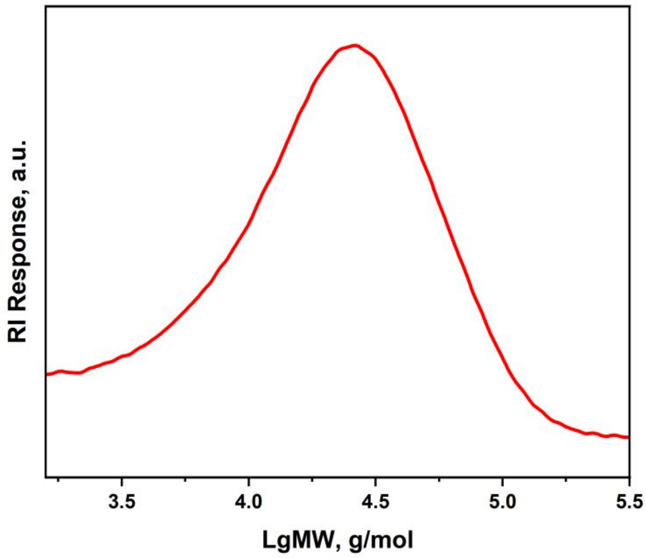

Figure S41. GPC data of the sample from entry 3, Table S1 after reaction of PS-2 with 4,4'-dithiodibutyric acid catalysed by 100 mol% DABCO.

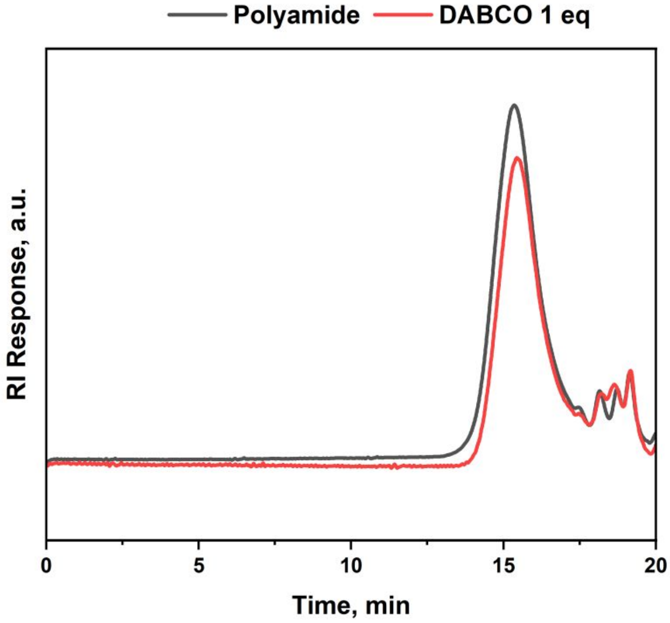

Figure S42. GPC data of the sample from entry 3, Table S1 after reaction of PS-2 with 4,4'-dithiodibutyric acid catalysed by 100 mol% DABCO (red) in comparision with virgin polymer PS-2.

3.2 Tricyclohexylphosphine Cy_3P-catalyzed disulfide exchange reaction.

Polymer PS-2 (100 mg, 0.136 mmol) and 4,4′-Dithiodibutyric acid (162 mg, 1.36 mmol) and Tricyclohexylphosphine (**Entry 1, Table S2:** 3.8 mg, 10% mol, **Entry 2, Table S2:** 19.1 mg, 50% mol, **Entry 3, Table S2:** 38.1 mg, 100% mol) were placed in 8 mL vial, then chloroform (5 mL) was added and the reaction mixture was stirred at 100 °C for 19 h. After the reaction was cooled down to room temperature, a precipitate was observed. The precipitate was filtered off and washed with chloroform. The mother liquor was evaporated to dryness and washed with methanol and diethyl ether and dried in a vacuum, leaving a yellowish residue. The residue was dissolved in dicholromethane (30 mL) and washed with 1 M solution of sodium hydroxide (30 mL). The organic layer was collected and dried over sodium sulfate, and the solvent was evaporated. The residue was dried on vacuum.

Table S4. Degradation of Polyamide PS-2 via Cy_3P-catalyzed sulfur-sulfur bond metathesis reaction.[a]

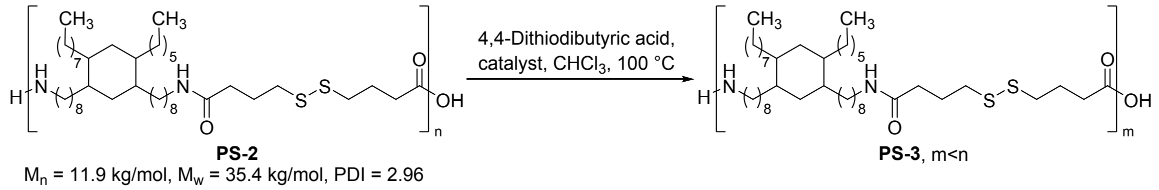

Entry	Catalyst	Mol. %	Isolated material, mg	M_n,[b] kg/mol	M_w,[b] kg/mol	PDI[b]
1		10	89	12.1	25.0	2.1
2	Cy_3P	50	91	7.3	17.3	2.3
3		100	84	8.0	20.1	2.5

[a] The polymer was analysed with GPC in THF at 35 °C relative to polystyrene standards.

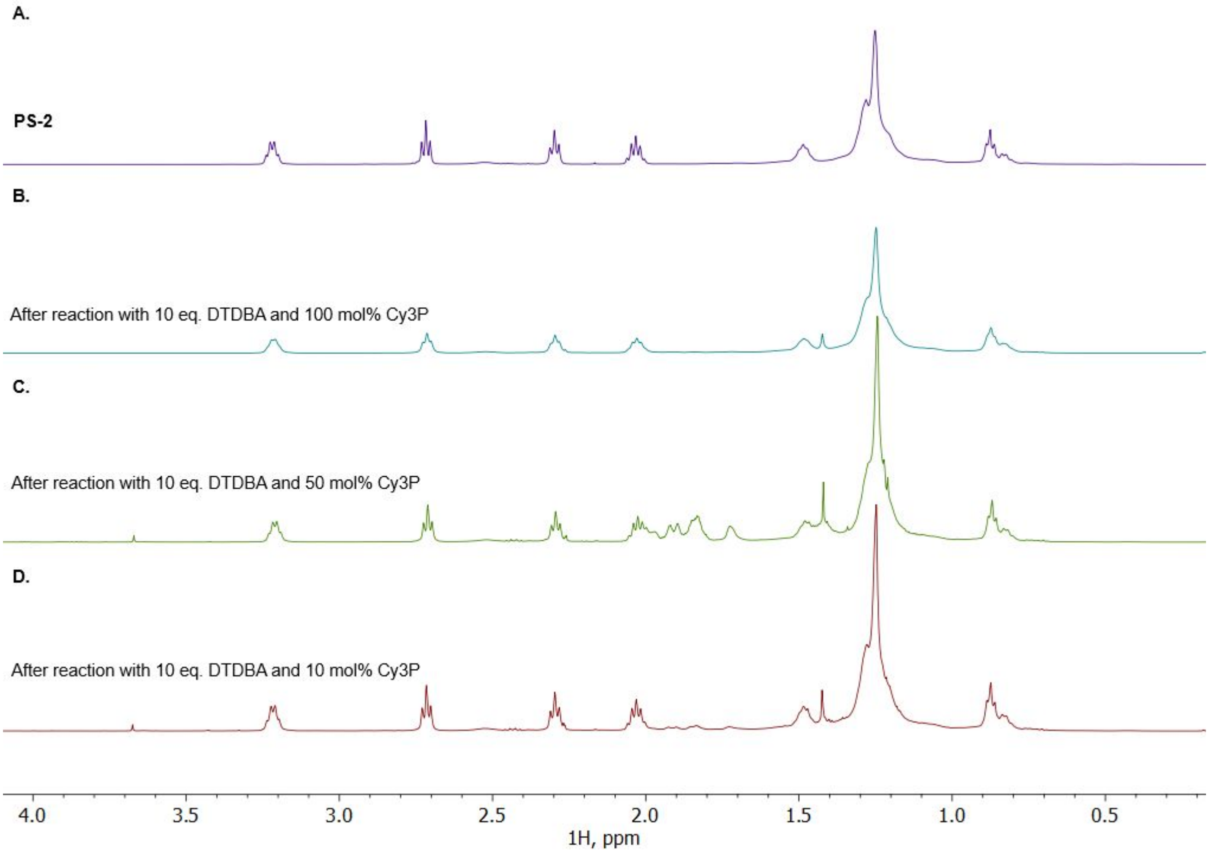

Figure S43. Fragments of ^{1}H (500 MHz, $CDCl_3$, 298 K) NMR spectra of **PS-2** and its reactions with DTDBA catalysed by Cy_3P. In spectrum **C** signals of Cy_3PO residue can also be seen.

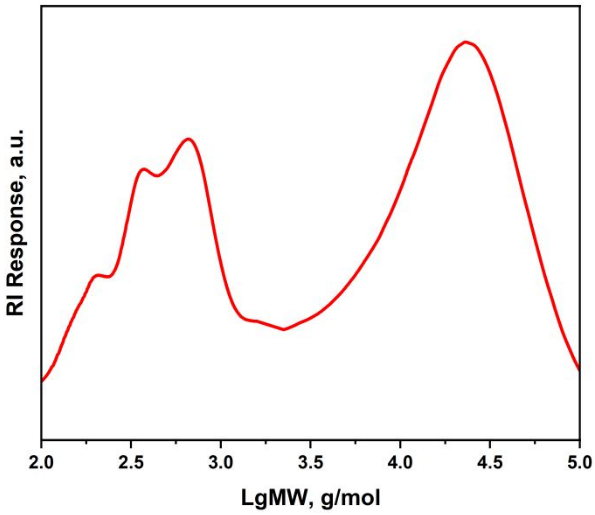

Figure S44. GPC analysis of the sample from entry 1, Table S2 after reaction of PS-2 with 4,4'-dithiodibutyric acid catalysed by 10 mol% Cy_3P.

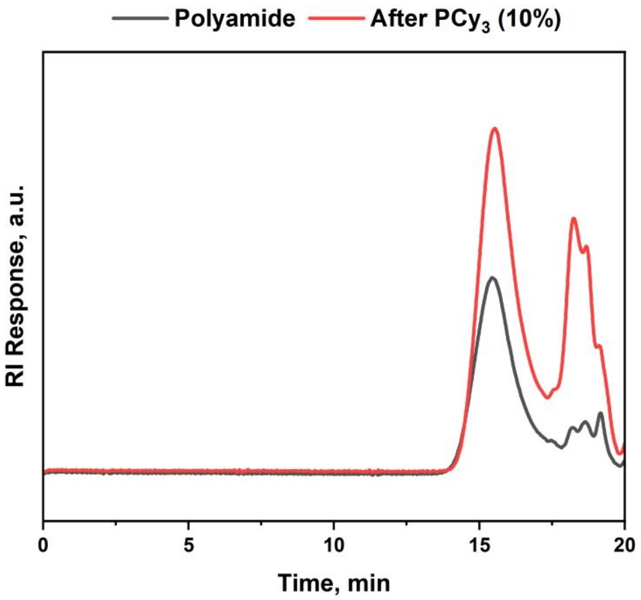

Figure S45. GPC chromatogram of the sample from entry 1, Table S2 after reaction of PS-2 with 4,4'-dithiodibutyric acid catalysed by 10 mol% Cy_3P (red) in comparision with virgin polymer PS-2 (black).

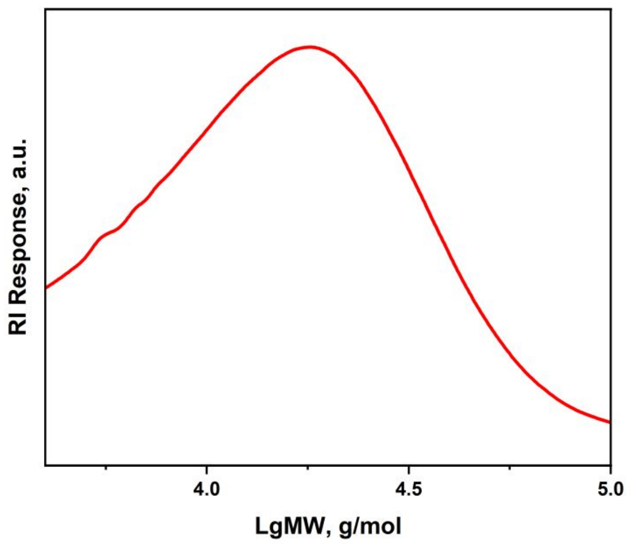

Figure S46. GPC analysis of the sample from entry 2, Table S2 after reaction of PS-2 with 4,4'-dithiodibutyric acid catalysed by 50 mol% Cy_3P.

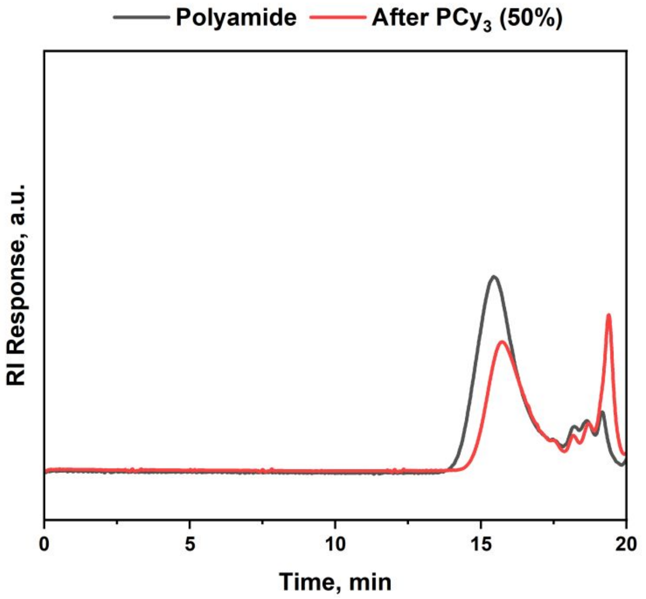

Figure S47. GPC chromatogram of the sample from entry 2, Table S2 after reaction of PS-2 with 4,4'-dithiodibutyric acid catalysed by 50 mol% Cy_3P (red) in comparision with virgin polymer PS-2 (black).

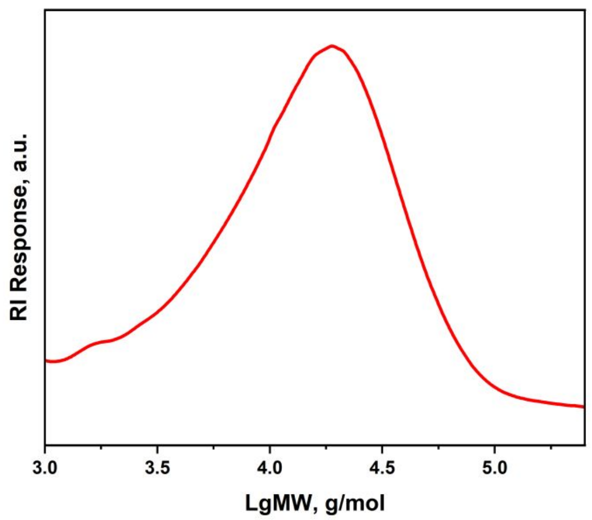

Figure S48. GPC analysis of the sample from entry 3, Table S2 after reaction of PS-2 with 4,4'-dithiodibutyric acid catalysed by 100 mol% Cy_3P.

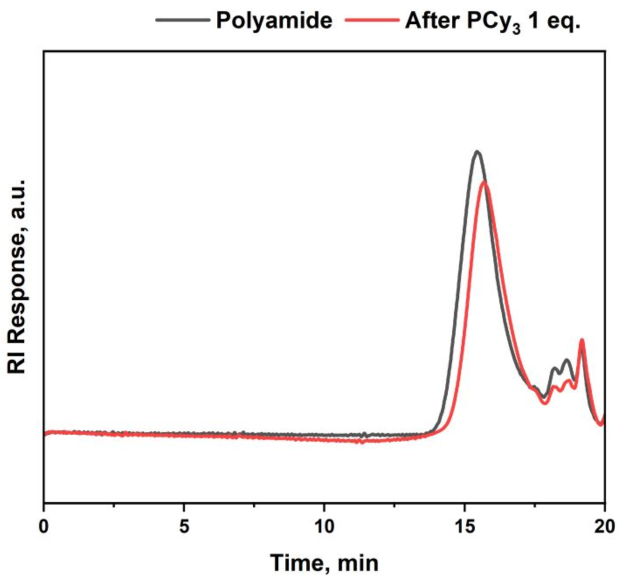

Figure S49. GPC chromatogram of the sample from entry 3, Table S2 after reaction of PS-2 with 4,4'-dithiodibutyric acid catalysed by 100 mol% Cy_3P (red) in comparision with virgin polymer PS-2 (black).

3.3 Triphenylphosphine Ph_3P-catalyzed disulfide exchange reaction.

Polymer PS-2 (100 mg, 0.136 mmol) and 4,4′-Dithiodibutyric acid (162 mg, 1.36 mmol) and Triphenylphosphine (**Entry 1, Table S3:** 3.5 mg, 10% mol, **Entry 2, Table S3:** 17.83 mg, 50% mol, **Entry 3, Table S3:** 35.67 mg, 100% mol) were placed in 8 mL vial, then chloroform (5 mL) was added and the reaction mixture was stirred at 100 °C for 19 h. After the reaction was cooled down to room temperature, a precipitate formed. The precipitate was filtered off and washed with chloroform. The mother liquor was evaporated to dryness and washed with methanol and diethyl ether and dried in vacuum, leaving a yellowish residue. The residue was dissolved in dichloromethane (30 mL) and washed with 1 M solution of sodium hydroxide (30 mL). The organic layer was collected and dried over sodium sulphate and the solvent was evaporated. The residue was dried on vacuum.

Table S5. Degradation of Polyamide PS-2 via Ph_3P-catalyzed sulfur-sulfur bond metathesis reaction.

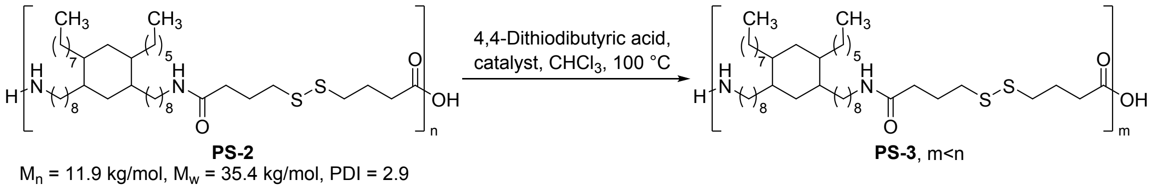

Entry	Catalyst	Mol. %	Isolated material, mg	M_n,[a] kg/mol	M_w,[a] kg/mol	PDI[a]
1		10	85	5.8	14.3	2.5
2	Ph_3P	50	76	3.4	7.3	2.2
3		100	58	2.4	5.9	2.4

[a] The polymer was analysed with GPC in THF at 35 °C relative to polystyrene standards.

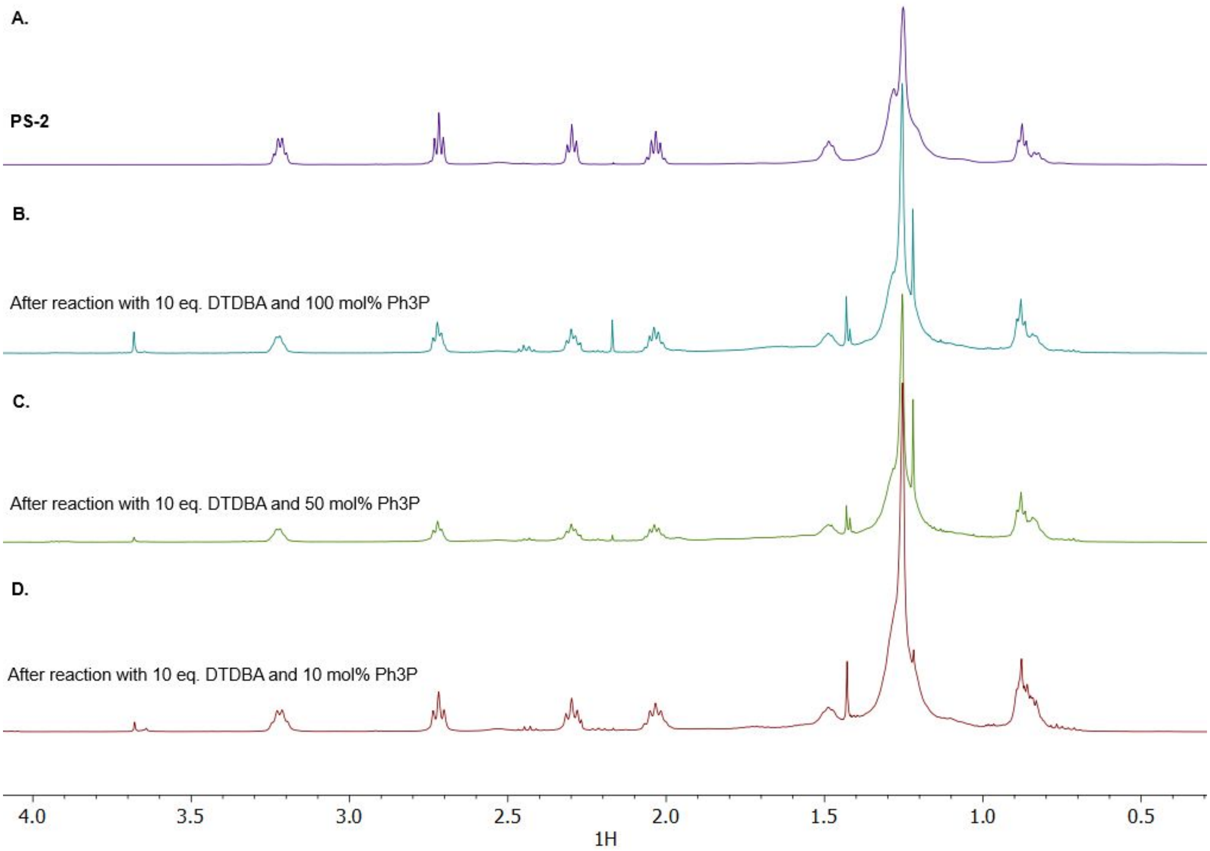

Figure S50. Fragments of ^{1}H (500 MHz, $CDCl_3$, 298 K) NMR spectra of **PS-2** and its reactions with DTDBA catalysed by Ph_3P.

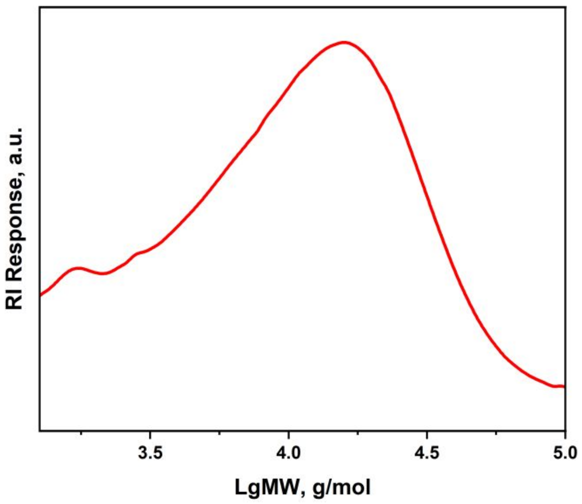

Figure S51. GPC analysis of the sample from entry 1, Table S3 after reaction of **PS-2** with 4,4'-dithiodibutyric acid catalysed by 10 mol% Ph_3P.

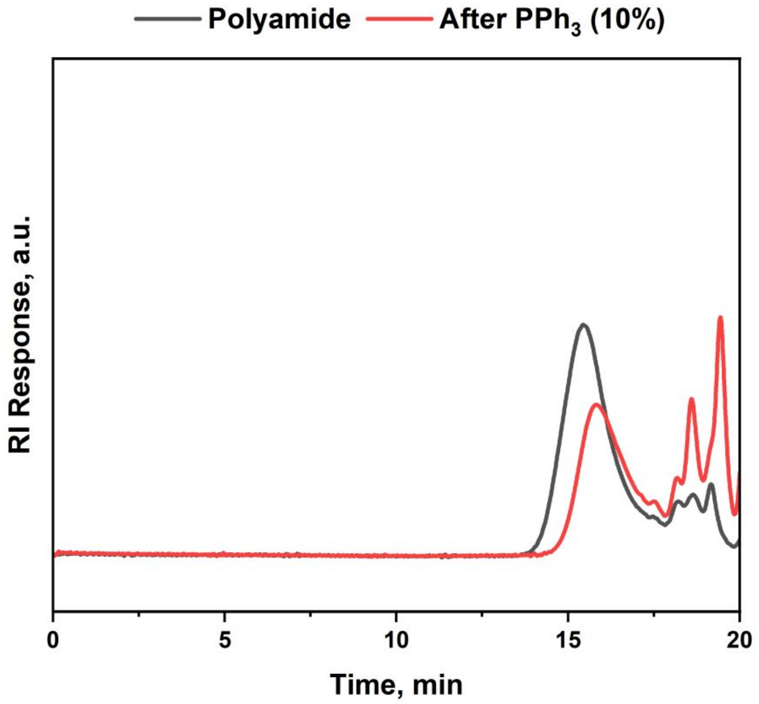

Figure S52. GPC chromatogram of the sample from entry 1, Table S3 after reaction of **PS-2** with 4,4'-dithiodibutyric acid catalysed by 10 mol% Ph_3P.

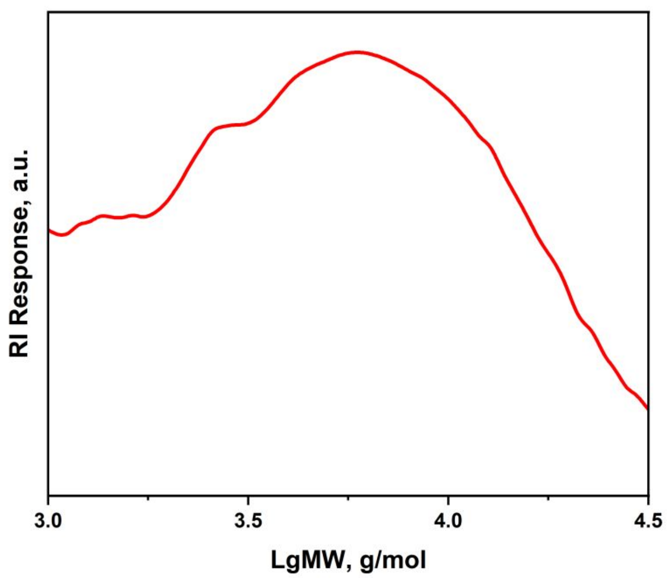

Figure S53. GPC analysis of the sample from entry 2, Table S3 after reaction of **PS-2** with 4,4'-dithiodibutyric acid catalysed by 50 mol% Ph_3P.

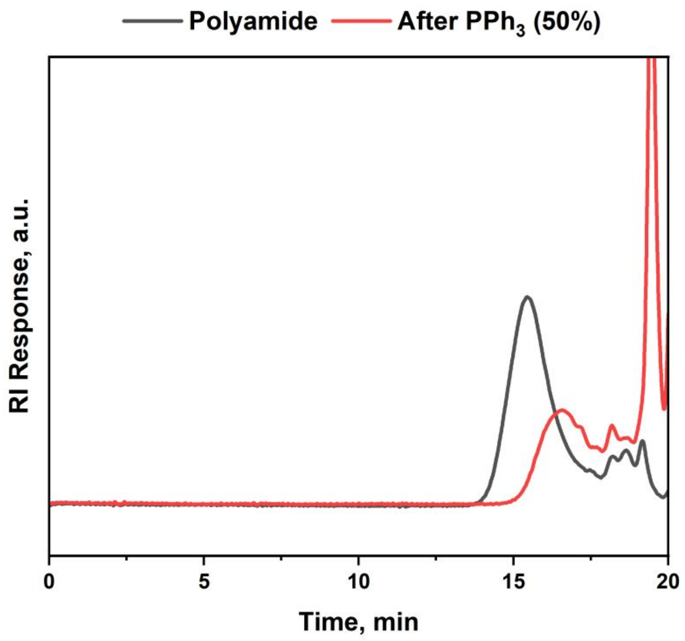

Figure S54. GPC chromatogram of the sample from entry 2, Table S3 after reaction of **PS-2** with 4,4'-dithiodibutyric acid catalysed by 50 mol% Ph_3P.

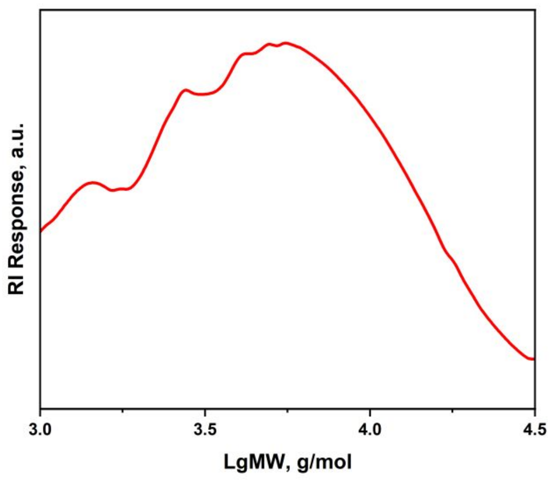

Figure S55. GPC analysis of the sample from entry 3, Table S3 after reaction of **PS-2** with 4,4'-dithiodibutyric acid catalysed by 100 mol% Ph_3P.

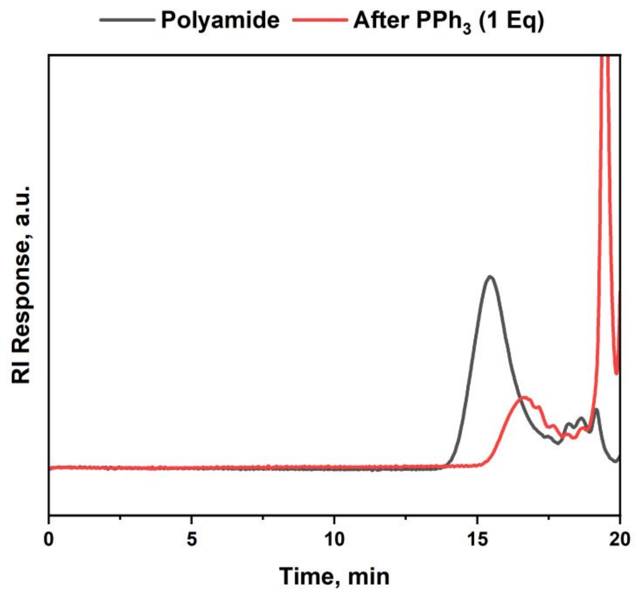

Figure S56. GPC chromatogram of the sample from entry 3, Table S3 after reaction of **PS-2** with 4,4'-dithiodibutyric acid catalysed by 100 mol% Ph_3P.

3.4 Rhodium complex-catalysed disulfide exchange reaction.

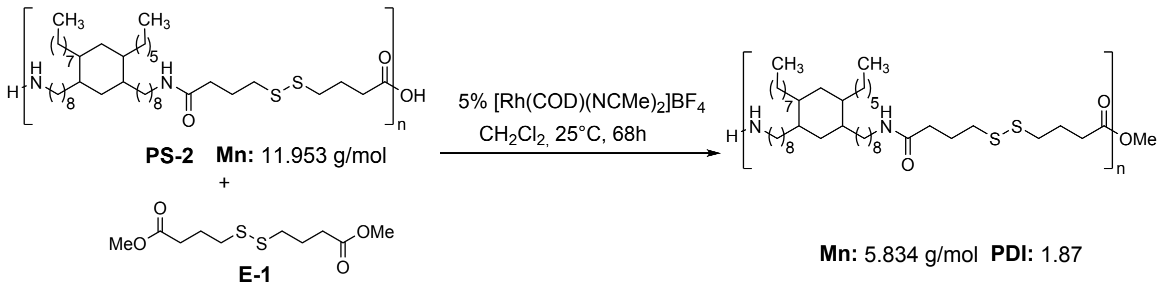

For characterization: Polymer **PS-2** (250 mg, 0.34 mmol) and dimethyl 4,4′-dithiodibutyrate (**E-1**, 900 mg, 3.4 mmol) and $[Rh(COD)(NCMe)_2]BF_4$ (6.45 mg, 5% mol) were sealed placed in 25 mL ampule, then dichloromethane (10 mL) was added and the reaction mixture was stirred at room temperature for 19h under argon. The solvent was evaporated, and the residue was washed with methanol, acetone and then dried on vacuum, giving 214 mg of brown residue (85.6% yield).

^{1}H NMR (400 MHz, $CDCl_3$): δ, 6.05 (br.s, 2H), 3.67 (s, 6H), 3.22 (m, 8H), 2.70 (m, 12H), 2.44 (m, 4H), 2.29 (m, 4H), 2.01 (m, 12H), 1.46 (m, 11H), 1.24 (m, 98H), 0.86 (m, 16H).

$^{13}C\{^{1}H\}$ NMR (100 MHz, $CDCl_3$): δ, 173.3, 172.1, 51.5, 39.5, 38.0, 37.6, 34.6, 32.2, 31.8, 29.5, 26.9, 24.8, 24.0, 22.6, 14.0.

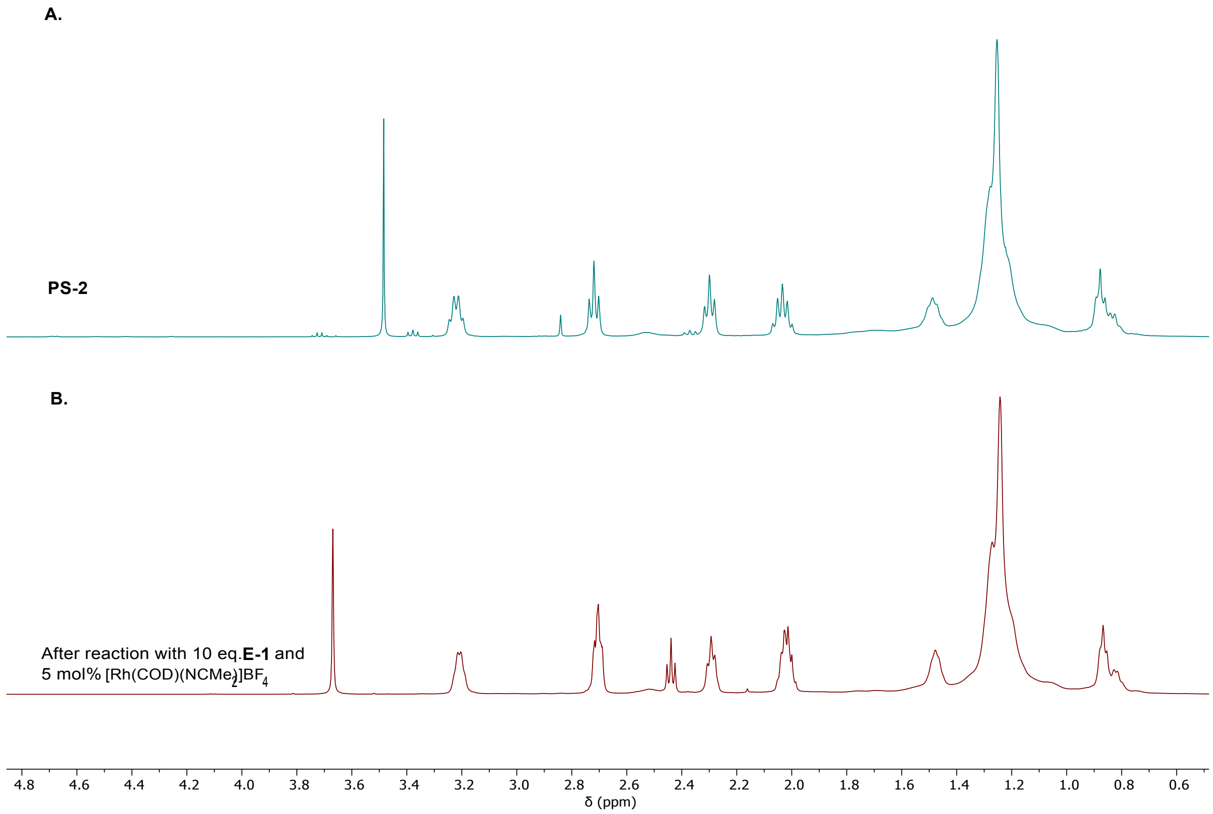

Figure S57. Fragments of ^{1}H (500 MHz, $CDCl_3$, 298 K) NMR spectra of **PS-2** and its reactions with **E-1** catalysed by $[Rh(COD)(NCMe)_2]BF_4$.

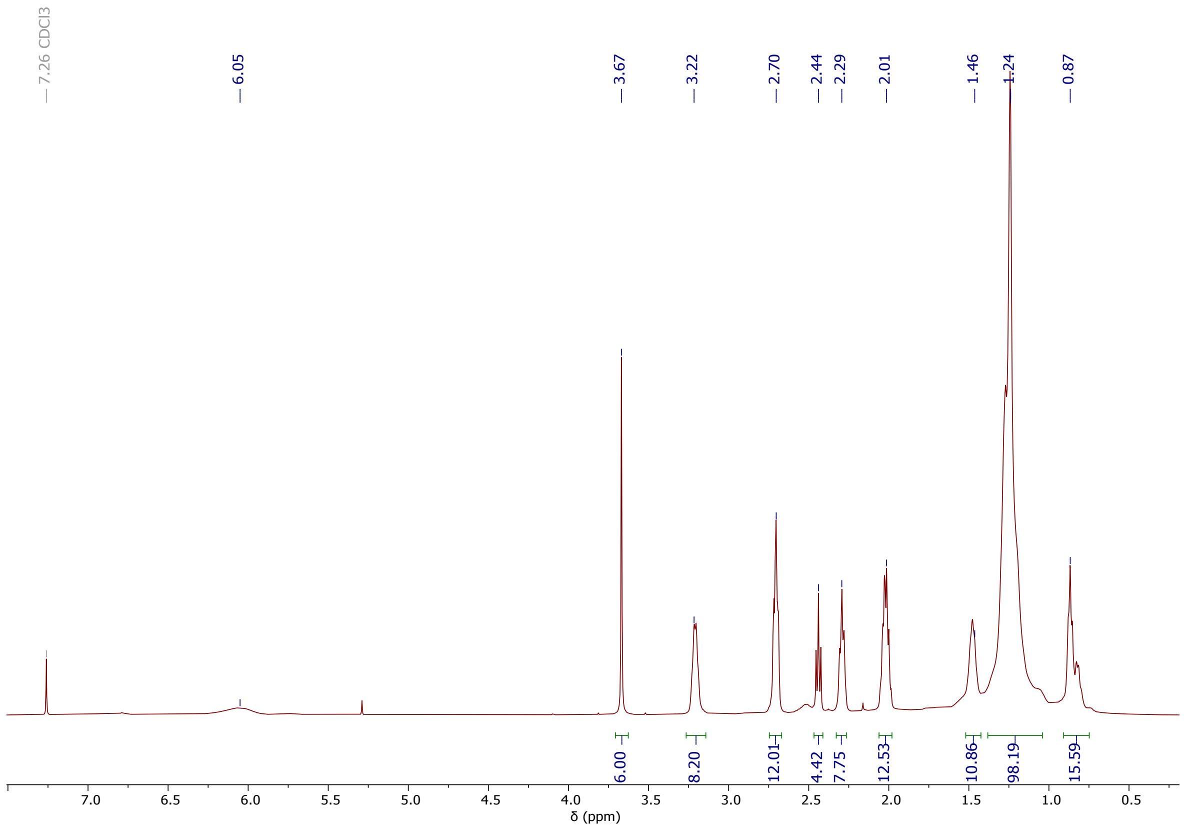

Figure S58. ^{1}H NMR (500 MHz, $CDCl_3$, 298 K) spectrum of product of reaction of **PS-1** with **E-1** catalysed by $[Rh(COD)(NCMe)_2]BF_4$.

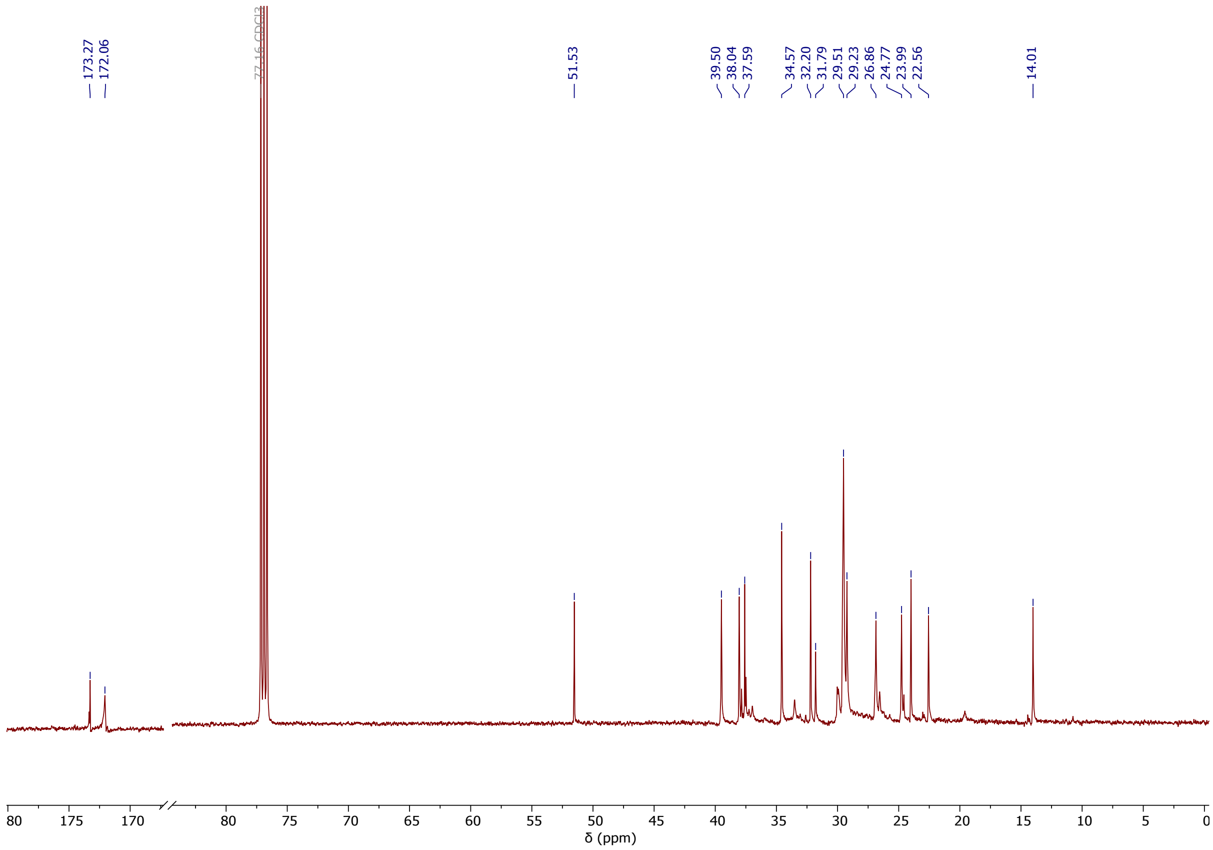

Figure S59. $^{13}C\{^{1}H\}$ NMR (125 MHz, $CDCl_3$, 298 K) spectrum of product of reaction of **PS-1** with **E-1** catalysed by $[Rh(COD)(NCMe)_2]BF_4$.

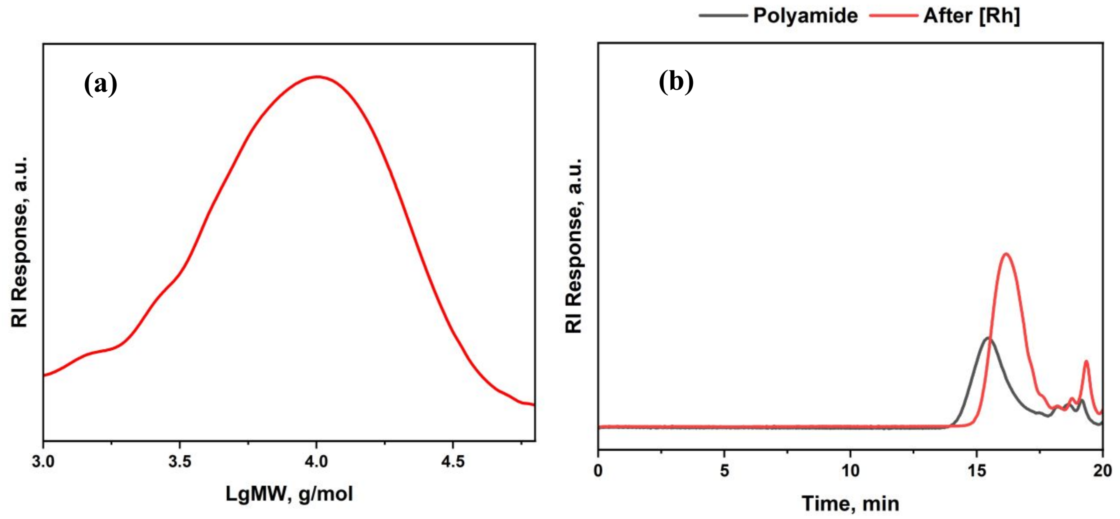

Figure S60. (a) GPC analysis of the sample after Rh-catalysed reaction of **PS-2** with dimethyl 4,4′-dithiodibutyrate. Mn: 5.8 kg/mol PDI: 1.87. **(b)** GPC chromatogram of the sample after Rh-catalysed reaction of **PS-2** with dimethyl 4,4′-dithiodibutyrate (red) in comparison with virgin polymer PS-2 (black).

3.5 Disulfide metathesis under UV-irradiation

For entry 1, Polymer **PS-2** (50 mg, 0.068 mmol) and dimethyl 4,4′-dithiodibutyrate (**E-1**, 181 mg, 0.68 mmol), in 5 ml of THF were stirred at room temperature for 19 or 66 h, in a sealed vial under argon atmosphere and UV Light irradiation (Hg lamp, emission wavelength range 250 – 400 nm, 100W) with a fan cooling. THF was evaporated and the sample was dried under vacuum. The products were analysed by NMR spectroscopy to estimate polymer conversion and GPC to estimate molecular mass of the products.

For entry 2, Polymer **PS-2** (1 g, 1.36 mmol) and dimethyl 4,4′-dithiodibutyrate (**E-1**, 3.6 g, 13.6 mmol), in 20 ml of THF were stirred at room temperature for 68 h, in a sealed ampule under argon atmosphere and UV Light irradiation (Hg lamp, emission wavelength range 250 – 400 nm, 100W) with a fan cooling. THF was evaporated and the sample was dried under vacuum. The product was isolated column chromatography with dichloromethane/ethyl acetate as eluent to obtain 0.855 g (85% yield) of a viscous yellow oil.

^{1}H NMR (400 MHz, $CDCl_3$): δ, 5.93 (br.s, 2H), 3.65 (s, 6H), 3.21 (q, J = 6.7 Hz, 4H), 2.70 (m, 8H), 2.43 (t, J = 7.2 Hz, 4H), 2.28 (t, J = 7.3 Hz, 3H), 2.02 (m, 9H), 1.45 (m, 4H), 1.23 (m, 43H), 0.86 (m, 7H).

^{13}C{^{1}H} NMR (100 MHz, $CDCl_3$): δ, 173.7, 172.2, 51.8, 39.8, 38.3, 37.8, 34.8, 32.5, 32.0, 29.8, 27.1, 24.8, 24.3, 22.8, 14.2.

HRMS-EI (m/z) calculated for $C_{54}H_{102}N_2NaO_6S_4^+$ $[M+Na]^+$, 1025.6513; found, 1025.6589

Table S6. Degradation of polyamide PS-2 via UV-initiated sulfur-sulfur bond metathesis reactions.

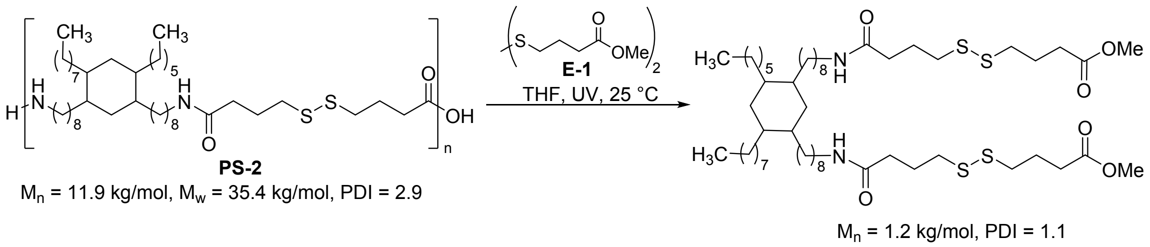

Entry	Catalyst	Reaction time, h	Conversion %	M_n,[a] kg/mol	M_w,[a] kg/mol	PDI[a]
1	100 W Hg-lamp	19	81[b]	1.2	1.8	1.4
2	100 W Hg-lamp	68	85[c]	1.3	1.4	1.1

[a] The polymer was analysed with GPC in THF at 35 °C using polystyrene standards. **[b]** Estimated with ^{1}H NMR using dichloromethane as internal standard. **[c]** Estimated by weight of purified product after column chromatography.

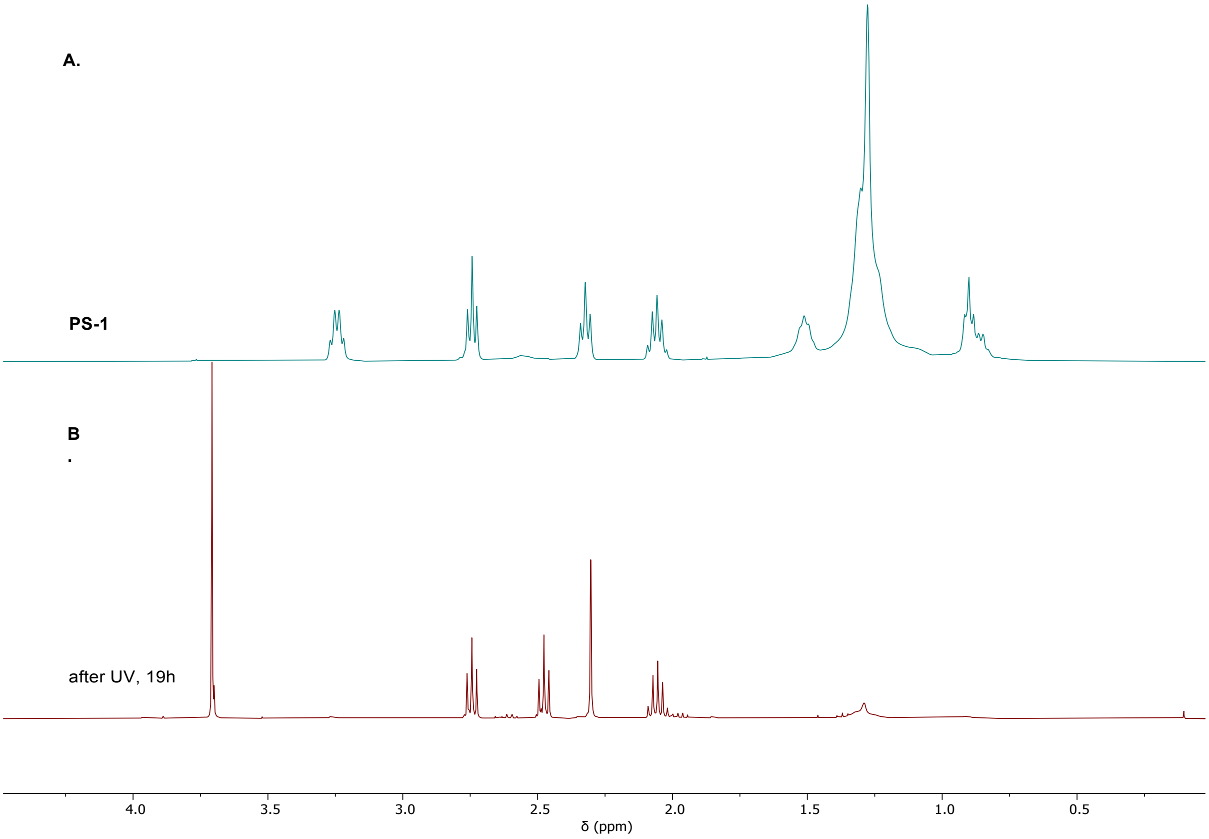

Figure S61. Fragments of ^{1}H NMR (500 MHz, $CDCl_3$, 298 K) NMR spectra of **PS-2** and its reactions with **E-1** under UV-irradiation.

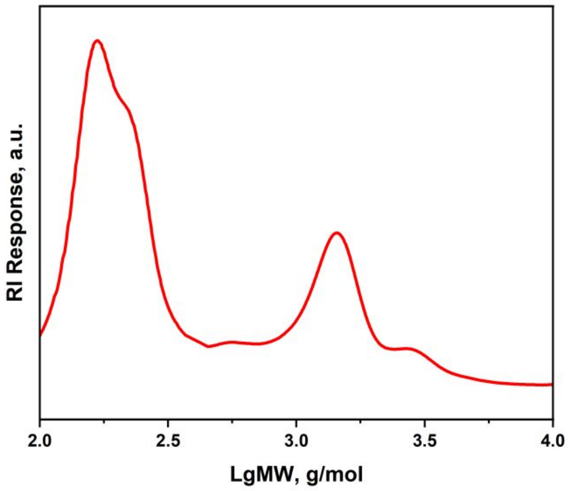

Figure S62. GPC analysis of the sample from Entry 1, Table S4 after reaction of **PS-2** with dimethyl 4,4′-dithiodibutyrate under UV-irradiation for 19 h.

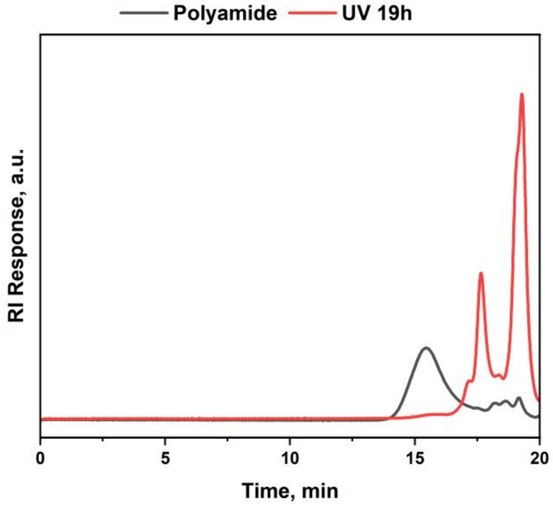

Figure S63. GPC chromatogram of the sample from Entry 1, Table S4 after reaction of **PS-2** with dimethyl 4,4′-dithiodibutyrate under UV-irradiation for 19 h (red) in comparision with virgin polymer **PS-2** (black).

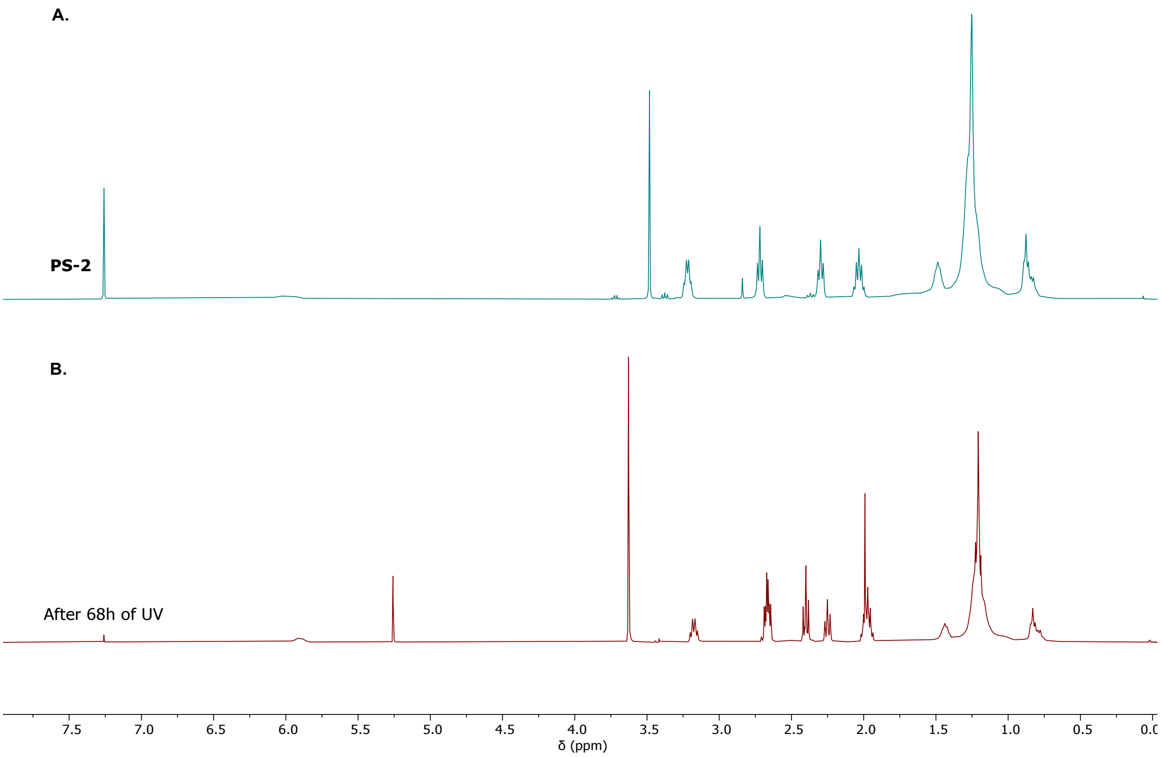

Figure S64. Fragments of ^{1}H NMR (500 MHz, $CDCl_3$, 298 K) NMR spectra of **PS-2** and its reaction with **E-1** under UV-irradiation for 68h.

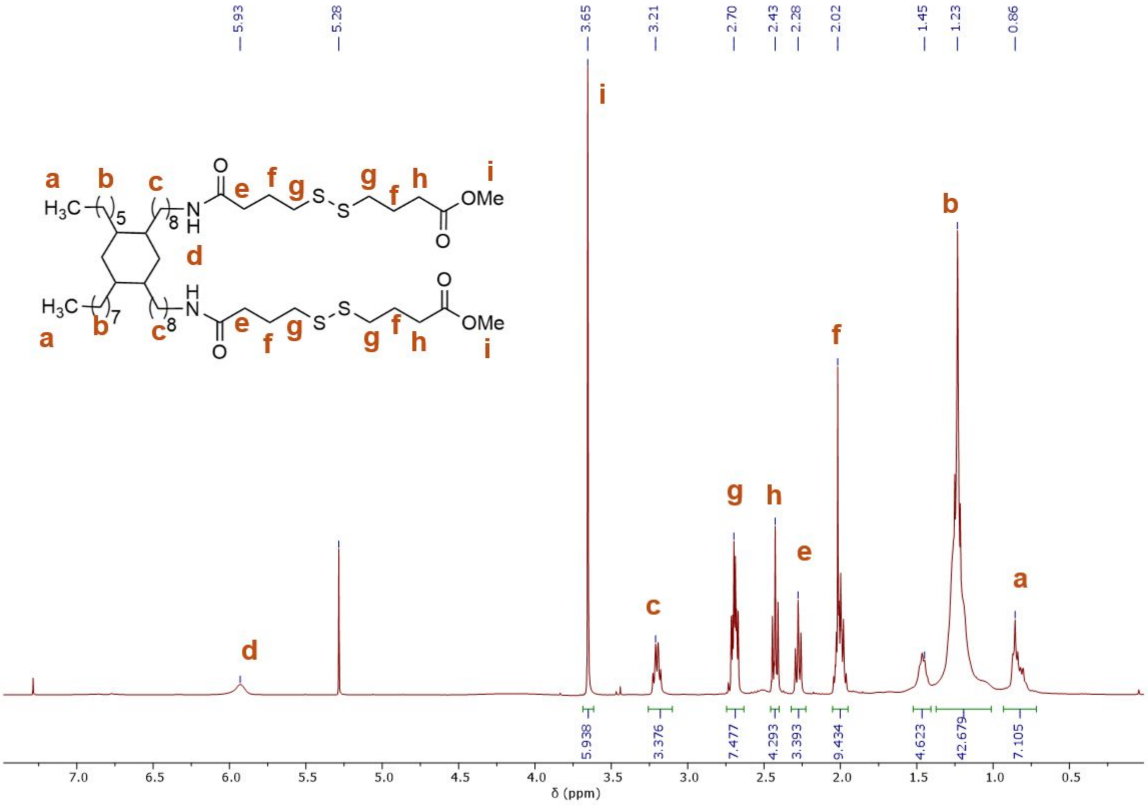

Figure S65. ^{1}H NMR (500 MHz, $CDCl_3$, 298 K) spectrum of product of reaction of **PS-2** with **E-1** catalysed by UV-Radiation for 68 h.

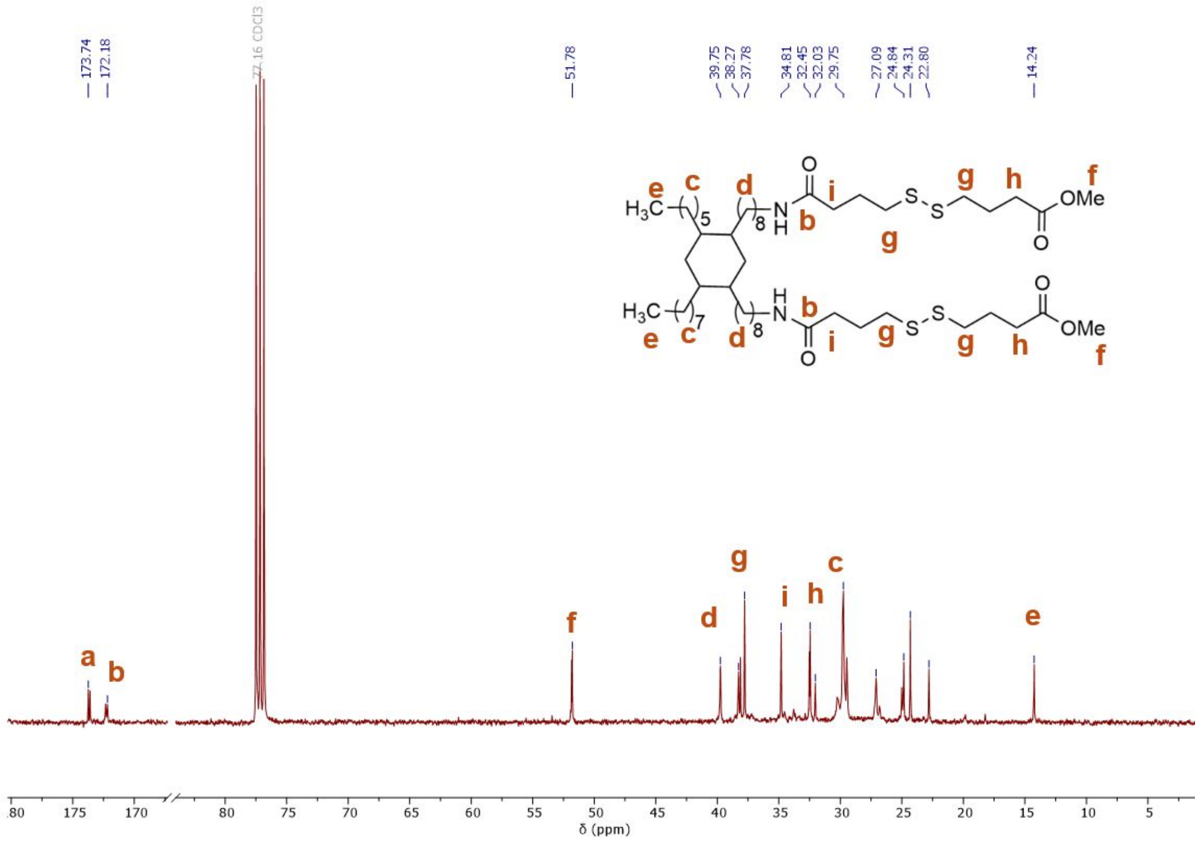

Figure S66.^{13}C NMR (125 MHz, $CDCl_3$, 298 K) spectrum of product of reaction of **PS-2** with **E-1** catalysed by UV-radiation for 68 h.

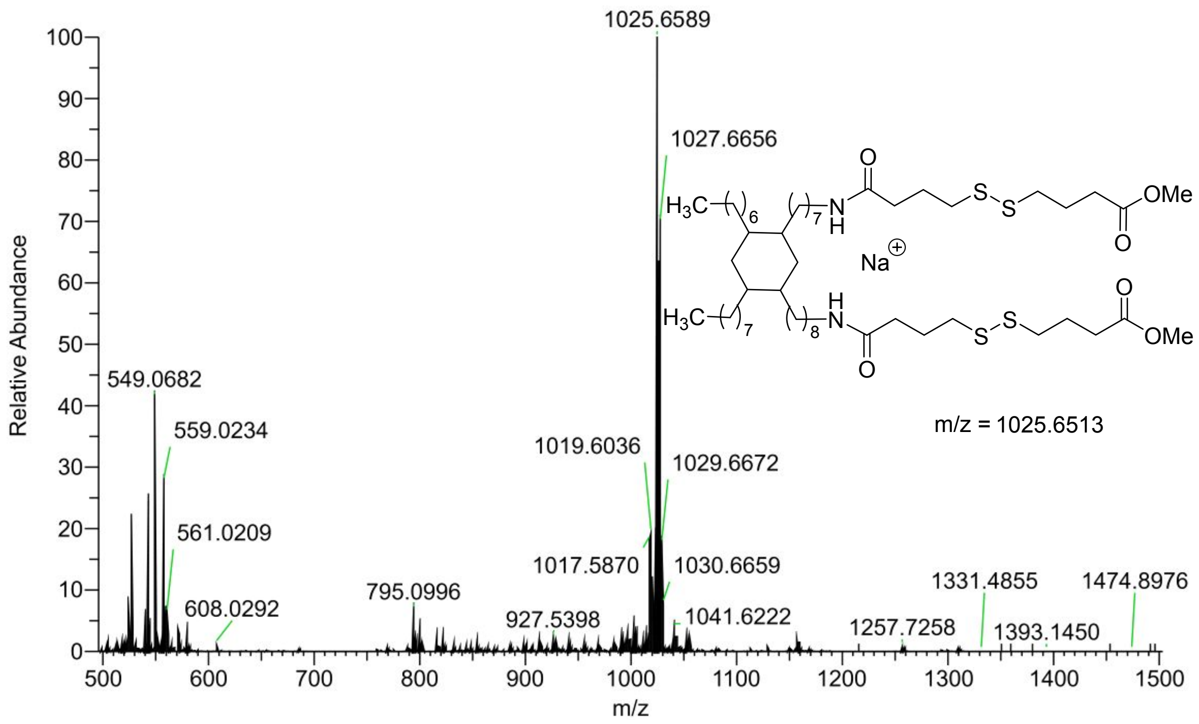

Figure S67. HRMS spectrum of reaction mixture of **PS-2** and with **E-1** under UV-irradiation for 68h.

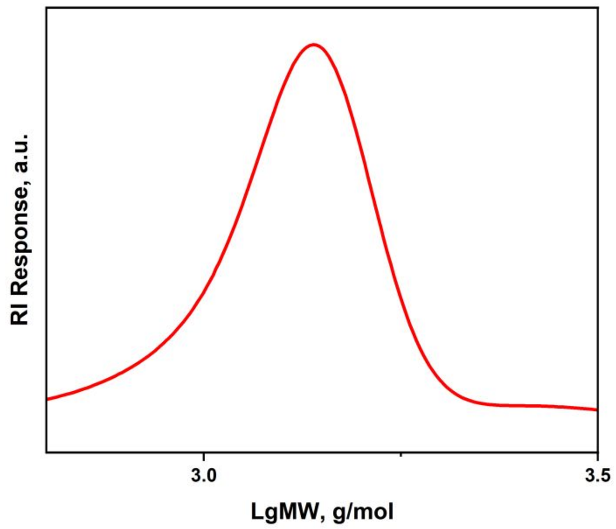

Figure S68. GPC analysis of the sample after UV-catalysed reaction for 68h of **PS-2** with dimethyl 4,4′-dithiodibutyrate. M_n: 1.2 kg/mol PDI: 1.1.

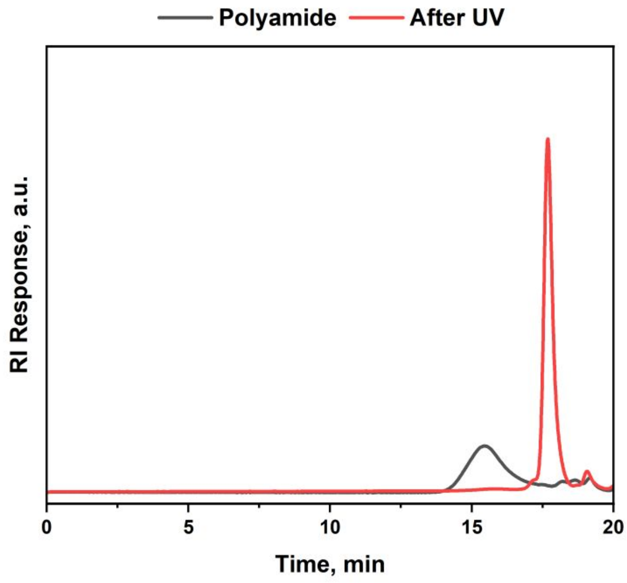

Figure S69. GPC chromatogram of the sample after UV-catalysed reaction of **PS-2** with dimethyl 4,4′-dithiodibutyrate (red) in comparison with virgin polymer PS-2 (black).

3.6 Disulfide metathesis under photoirradiation

For entry 1, polymer **PS-2** (250 mg, 0.34 mmol), dimethyl 4,4′-dithiodibutyrate (900 mg, 3.4 mmol) and $[Ir(dF(CF_3)ppy)_2(dtbpy)]PF_6$ (19 mg, 5% mol) were placed in an ampule under argon, then chloroform (10 mL) was added and the reaction mixture was stirred at room temperature for 19 h under blue LEDs (λ_{exc}=440 nm) irradiation. After the reaction was finished, chloroform was vacuumed off and the sample was purified via column chromatography (DCM:Ethyl Acetate) to obtain 243 mg (97% yield) of a yellow viscous oil. The solvent was evaporated, and the residue was analysed with GPC, HRMS-EI and NMR.

For Entry 2, polymer **PS-2** (50 mg, 0.068 mmol), octene-1 (76.2 mg, 0.68 mmol) and $[Ir(dF(CF_3)ppy)_2(dtbpy)]PF_6$ (3.8 mg, 5% mol) were placed in 8 mL vial under argon, then chloroform (5 mL) was added and the reaction mixture was stirred at room temperature for 19 h under blue LEDs

(λ_{exc}=440 nm) irradiation. The solvent was evaporated, and the residue was analysed with GPC and NMR.

Similarly, for entry 3, polymer **PS-2** (250 mg, 0.34 mmol), octene-1 (762 mg, 3.4 mmol) and [Ir(dF(CF$_3$)ppy)$_2$(dtbpy)]PF$_6$ (19 mg, 5% mol) were placed in an ampule under argon, then chloroform (10 mL) was added and the reaction mixture was stirred at room temperature for 68 h under blue LEDs (λ_{exc}=440 nm) irradiation. After the reaction was finished, chloroform was vacuumed off and the sample was purified via column chromatography (DCM:Ethyl Acetate) to obtain 169 mg (67% yield) of an orange viscous oil. The solvent was evaporated, and the residue was analysed with GPC and NMR.

Table S7. Degradation of polyamide PS-2 via photocatalytic sulfur-sulfur bond metathesis reactions.

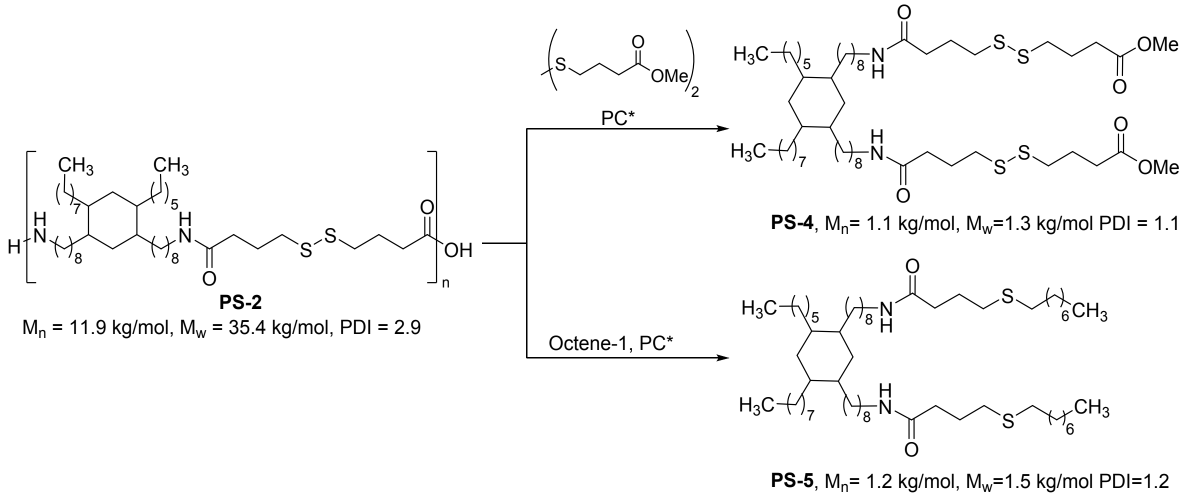

Entry	Catalyst	Conditions	yield[a] %	M_n,[b] kg/mol	M_w,[b] kg/mol	PDI[b]
1	5% [Ir(dF(CF$_3$)ppy)$_2$(dtbpy)]PF$_6$	10 eq. Ester, blue LEDs (λ_{exc}=440 nm), CHCl$_3$, RT, 19 h	97	1.1	1.3	1.1
2	5% [Ir(dF(CF$_3$)ppy)$_2$(dtbpy)]PF$_6$	10 eq. Octene-1, blue LEDs (λ_{exc}=440 nm), CHCl$_3$, RT, 68 h	67	1.4	3.4	2.5

[a] Estimated with mass of isolated material after column chromatography **[b]** The polymer was analyzed with GPC in THF at 35 °C relative to polystyrene standards. **[c]** Estimated with ^{1}H NMR using mesitylene as internal standard

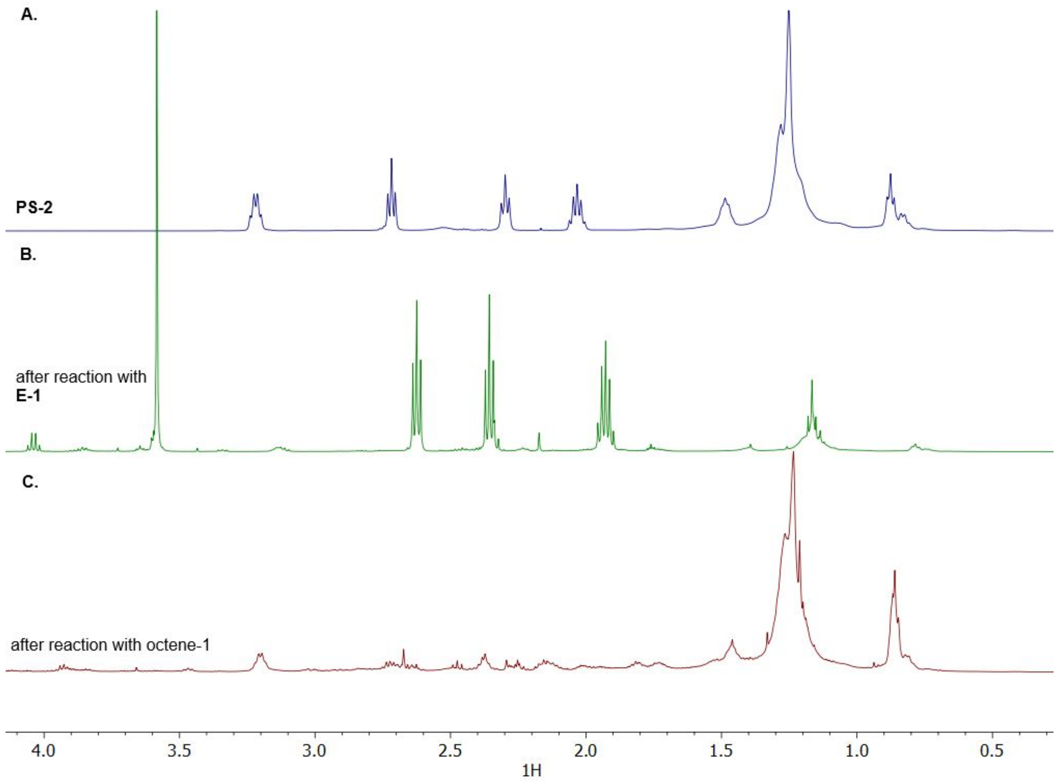

Figure S70. Fragments of ^{1}H NMR (500 MHz, $CDCl_3$, 298 K) NMR spectra of **PS-2** and its reactions with **E-1** and octene-1 under Blue LEDs (λ_{exc}=440 nm) irradiation.

3.6.1. Analysis of material isolated after reaction corresponding to Table S5, Entry 1.

^{1}H NMR (400 MHz, $CDCl_3$): δ, 5.61 (br.s, 2H), 3.67 (s, 6H), 3.21 (q, J = 6.8 Hz, 4H), 2.72 (q, J = 6.9 Hz, 8H), 2.44 (t, J = 7.2 Hz, 4H), 2.28 (t, J = 7.3 Hz, 3H), 2.02 (m, J = 7.0 Hz, 9H), 1.48 (m, 4H), 1.25 (m, 43H), 0.87 (m, 7H).

$^{13}C\{^{1}H\}$ NMR (100 MHz, $CDCl_3$): δ, 173.6, 172.05, 51.8, 39.7, 38.1, 37.8, 34.8, 32.5, 32.1, 29.8, 27.1, 24.8, 24.3, 22.8, 14.3.

HRMS-EI (m/z) calcd for $C_{54}H_{102}N_2NaO_6S_4^{+}$ $[M+Na]^{+}$, 1025.6513; found, 1025.6524

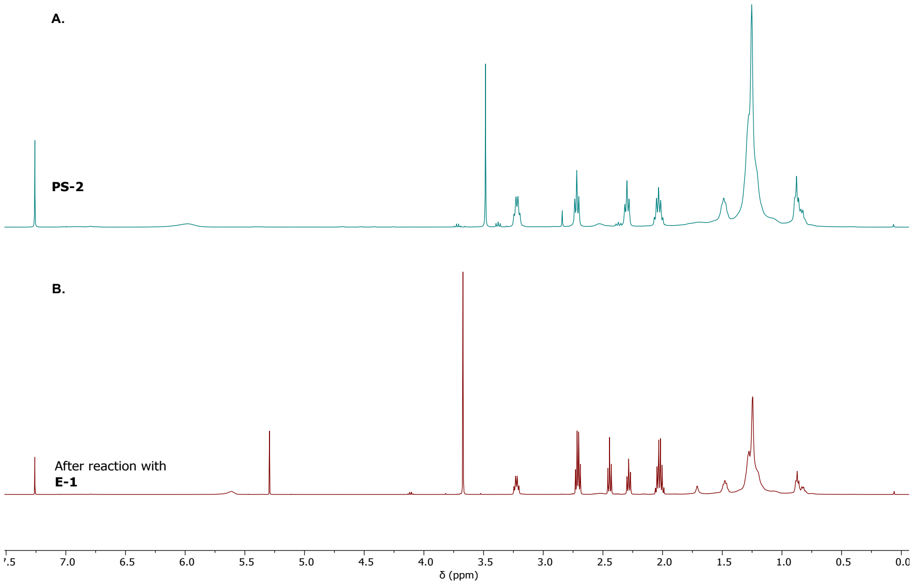

Figure S71. ^{1}H NMR (500 MHz, $CDCl_3$, 298 K) NMR spectra of **PS-2** and its reactions with **E-1** under Blue LEDs (λ_{exc}=440 nm) irradiation.

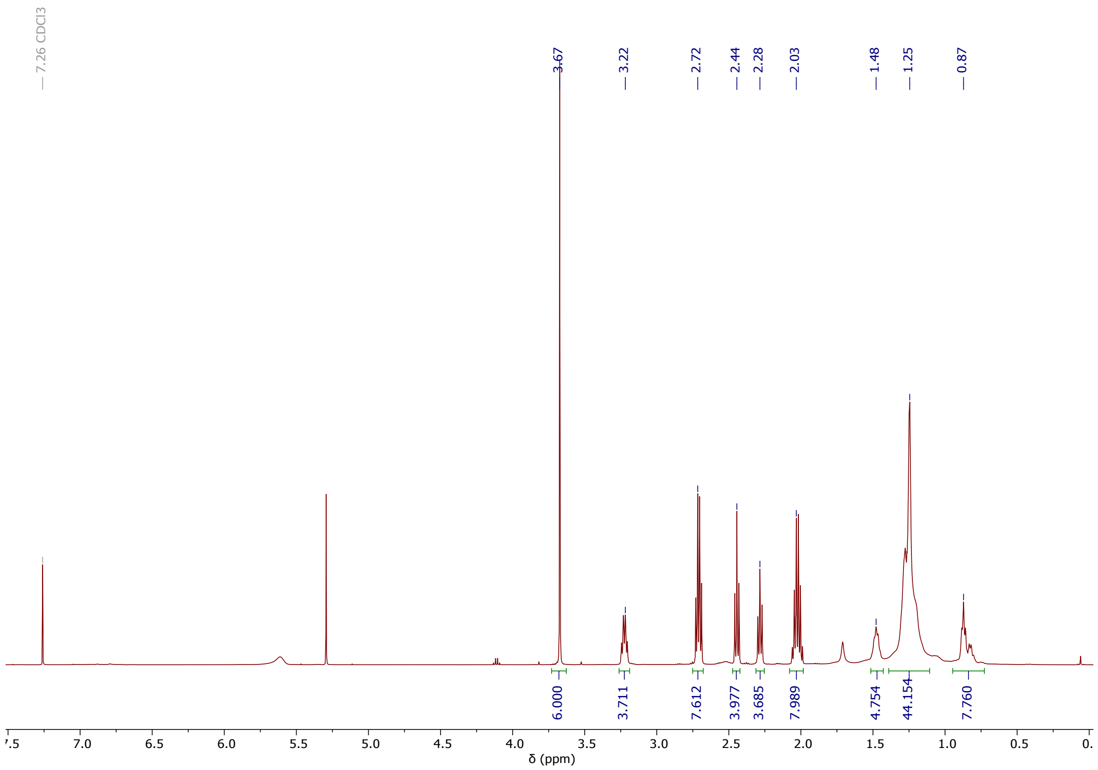

Figure S72. ^{1}H NMR (500 MHz, $CDCl_3$, 298 K) spectrum of product of reaction of **PS-2** with **E-1** catalysed by Blue LEDs (λ_{exc}=440 nm) irradiation.

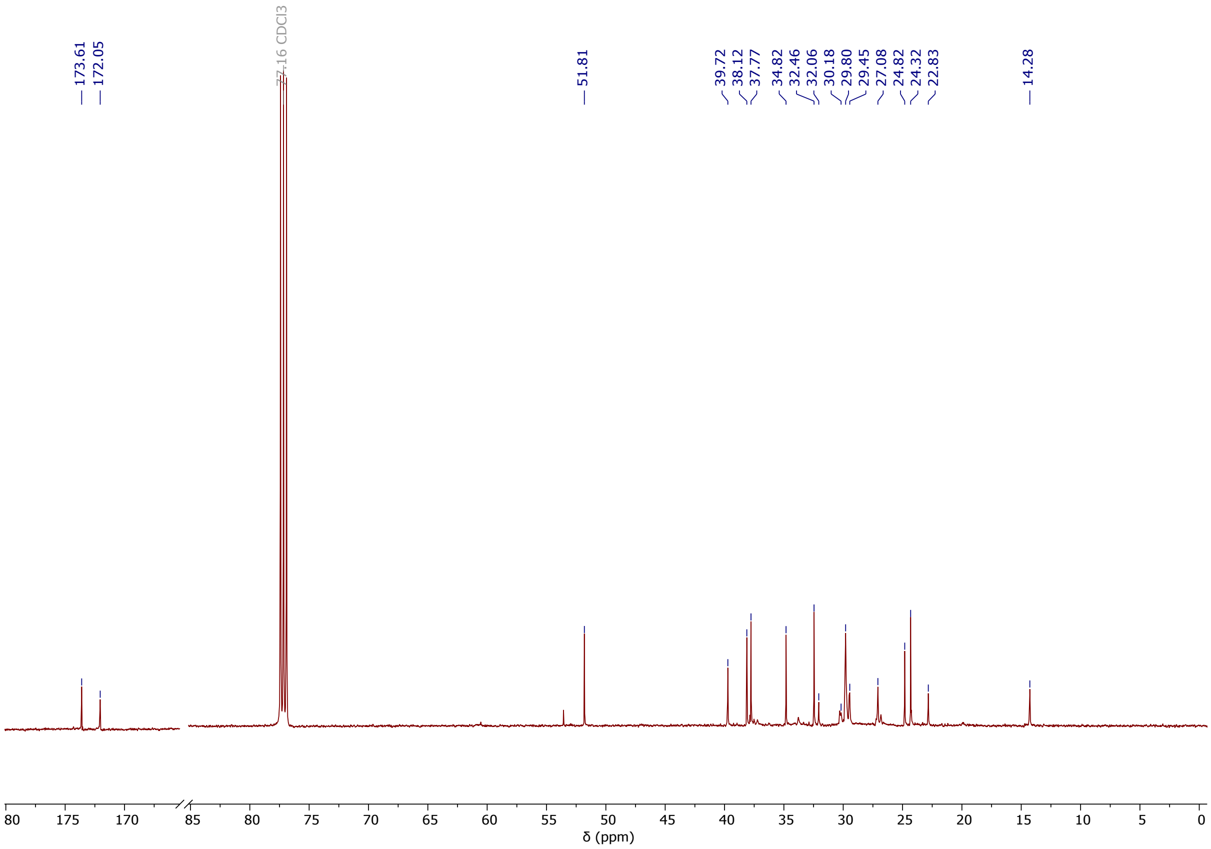

Figure S73.^{13}C NMR (125 MHz, $CDCl_3$, 298 K) spectrum of product of reaction of **PS-2** with **E-1** catalysed by Blue LEDs (λ_{exc}=440 nm) irradiation.

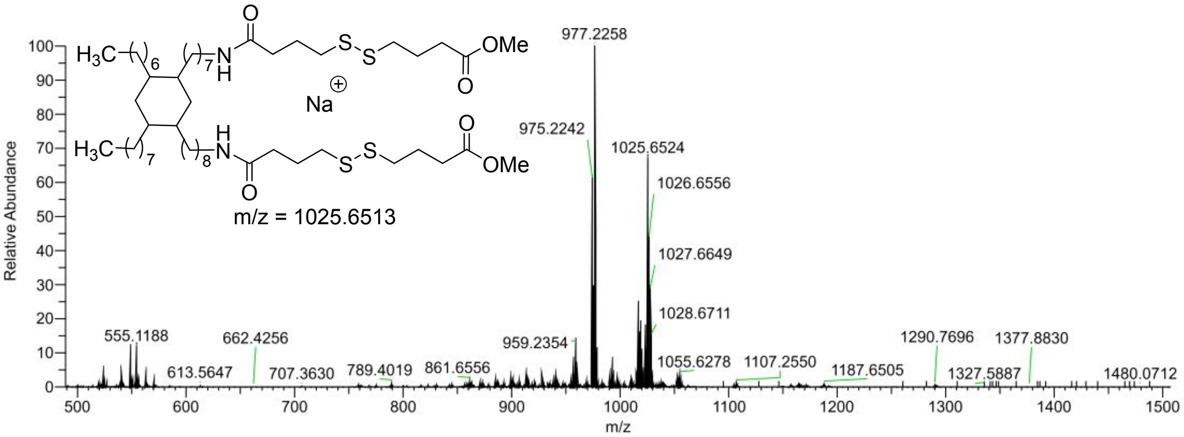

Figure S74. HRMS spectrum of reaction mixture of **PS-2** and with **E-1** under Blue LEDs (λ_{exc}=440 nm) irradiation.

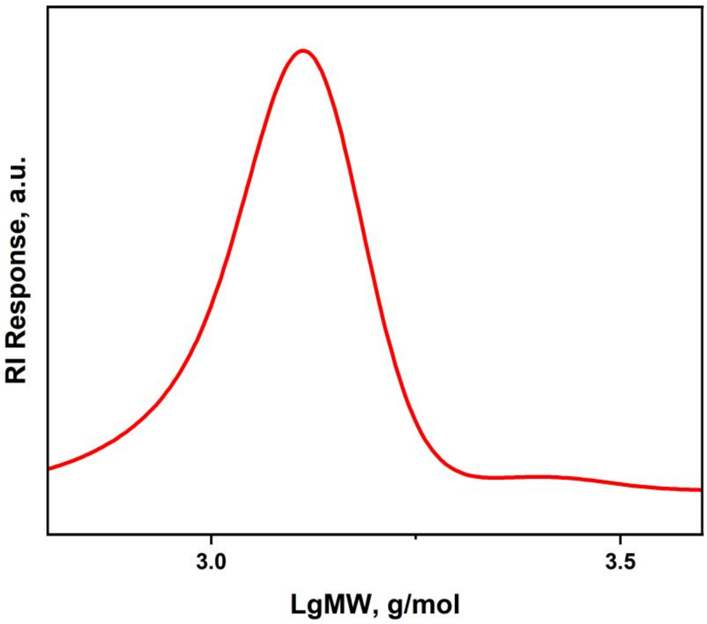

Figure S75. (a) GPC analysis of the sample after blue light catalyzed reaction of **PS-2** with dimethyl 4,4′-dithiodibutyrate. M_n: 1.1 kg/mol PDI: 1.1.

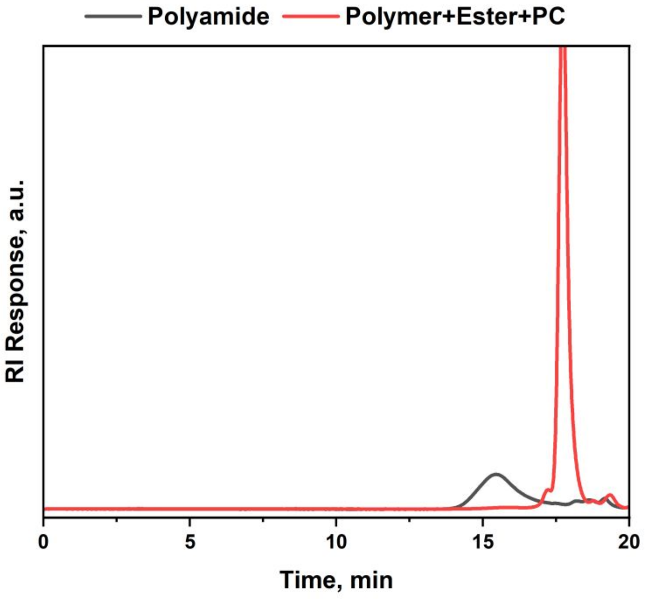

Figure S76. GPC chromatogram of the sample after blue light-catalysed reaction of **PS-2** with dimethyl 4,4′-dithiodibutyrate (red) in comparison with virgin polymer **PS-2** (black).

3.6.2. Analysis of material isolated after reaction corresponding to Table S5, Entry 2.

^{1}H NMR (400 MHz, $CDCl_3$): δ, 5.96 (br.s, 2H), 3.22 (m, 4H), 2.71 (m, 3H), 2.39 (m, 1H), 2.29 (t, J = 7.3 Hz, 3H), 2.02 (m, 6H), 1.47 (m, 8H), 1.25 (m, 56H), 0.87 (m, 10H).

$^{13}C\{^{1}H\}$ NMR (100 MHz, $CDCl_3$): δ, 172.4, 171.65, 39.7, 38.3, 34.8, 34.8, 32.1, 29.8, 27.1, 25.0, 24.3, 22.8, 14.3.

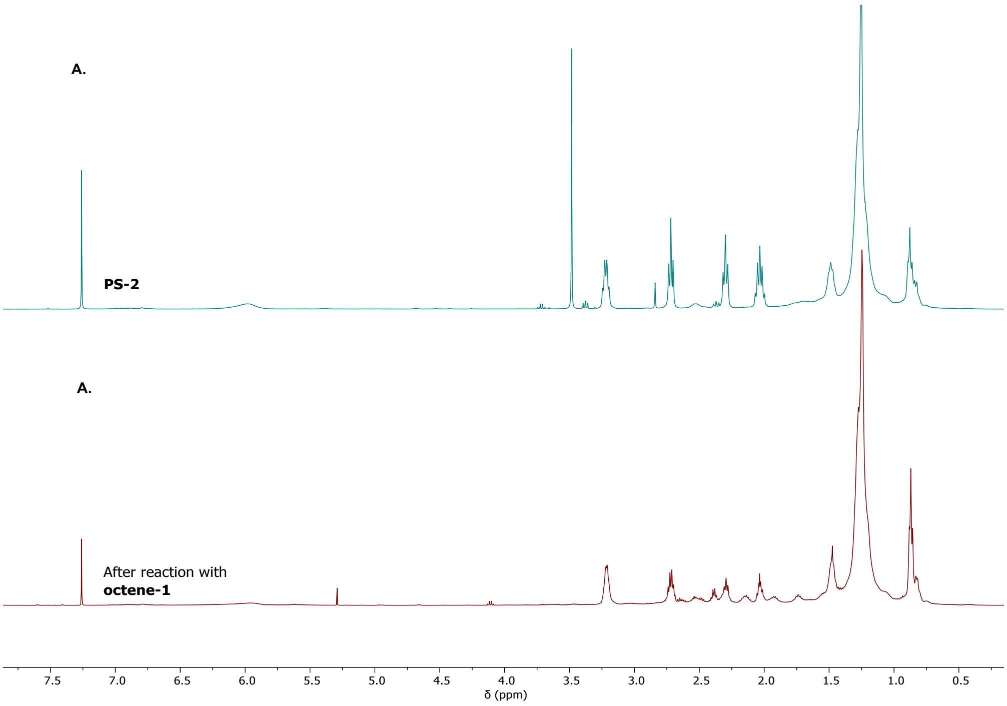

Figure S77.^{1}H NMR (500 MHz, $CDCl_3$, 298 K) NMR spectra of **PS-2** and its reactions with **octene-1** under Blue LEDs (λ_{exc}=440 nm) irradiation.

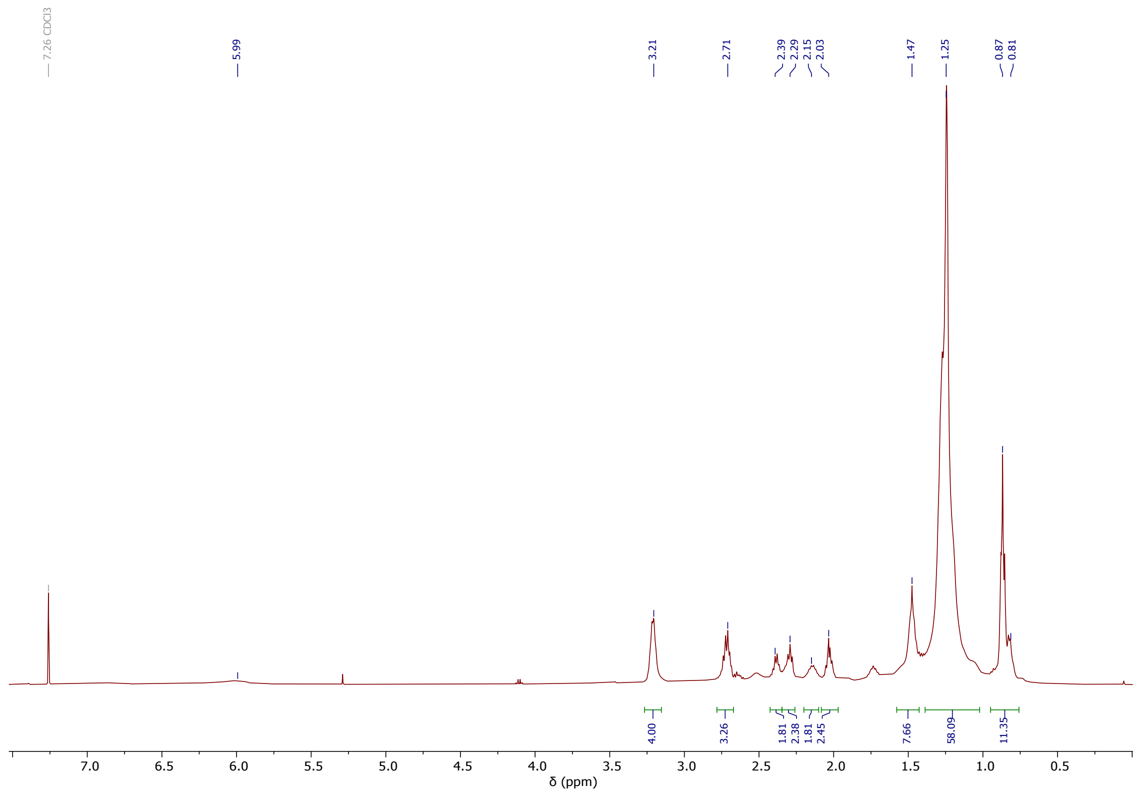

Figure S78.^{1}H NMR (500 MHz, $CDCl_3$, 298 K) spectrum of product of reaction of **PS-2** with **octene-1** catalysed by Blue LEDs (λ_{exc}=440 nm) irradiation.

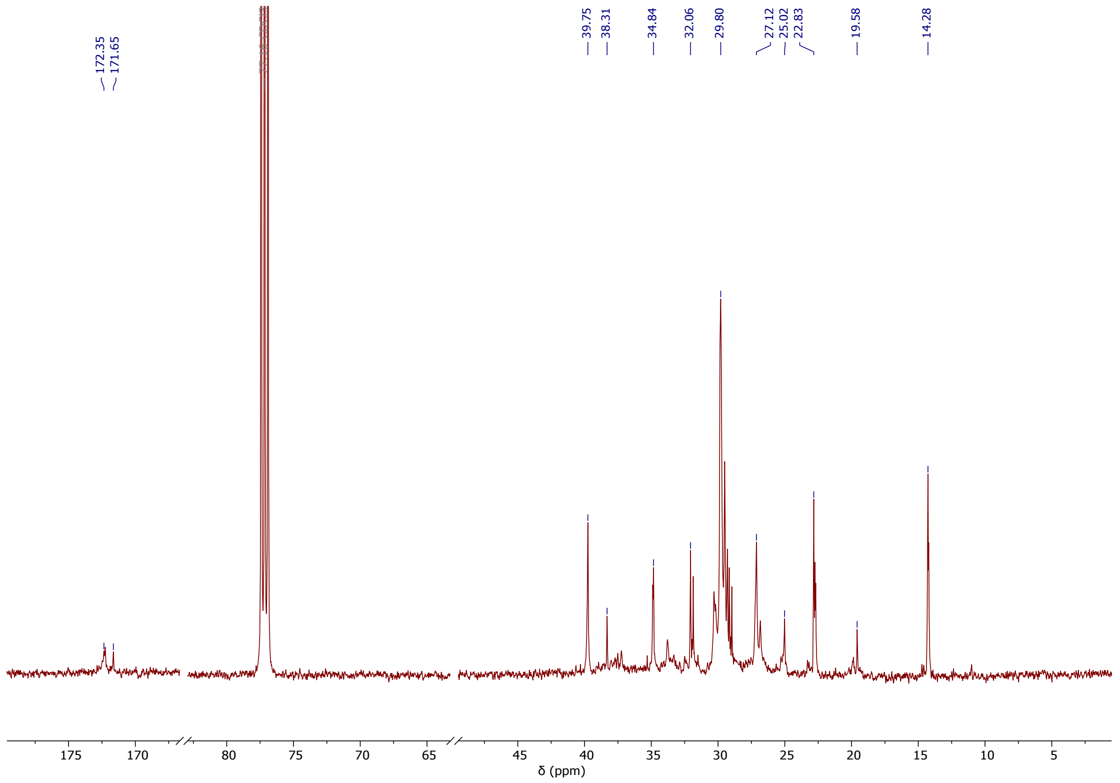

Figure S79. $^{13}C\{^{1}H\}$ NMR (125 MHz, $CDCl_3$, 298 K) spectrum of product of reaction of **PS-2** with **octene-1** catalysed by Blue LEDs (λ_{exc}=440 nm) irradiation.

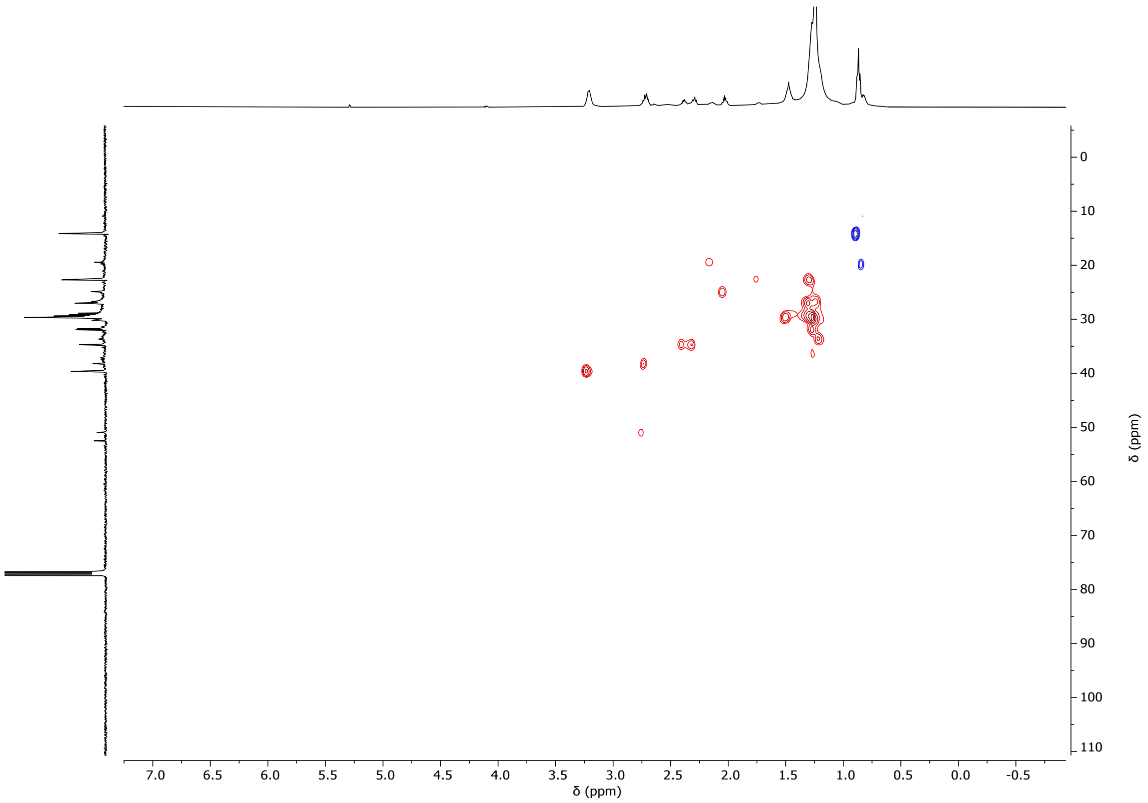

Figure S80. ^{1}H, ^{13}C- HSQC NMR ($CDCl_3$, 298 K) spectrum of product of reaction of **PS-2** with octene-1 catalysed by Blue LEDs (λ_{exc}=440 nm) irradiation.

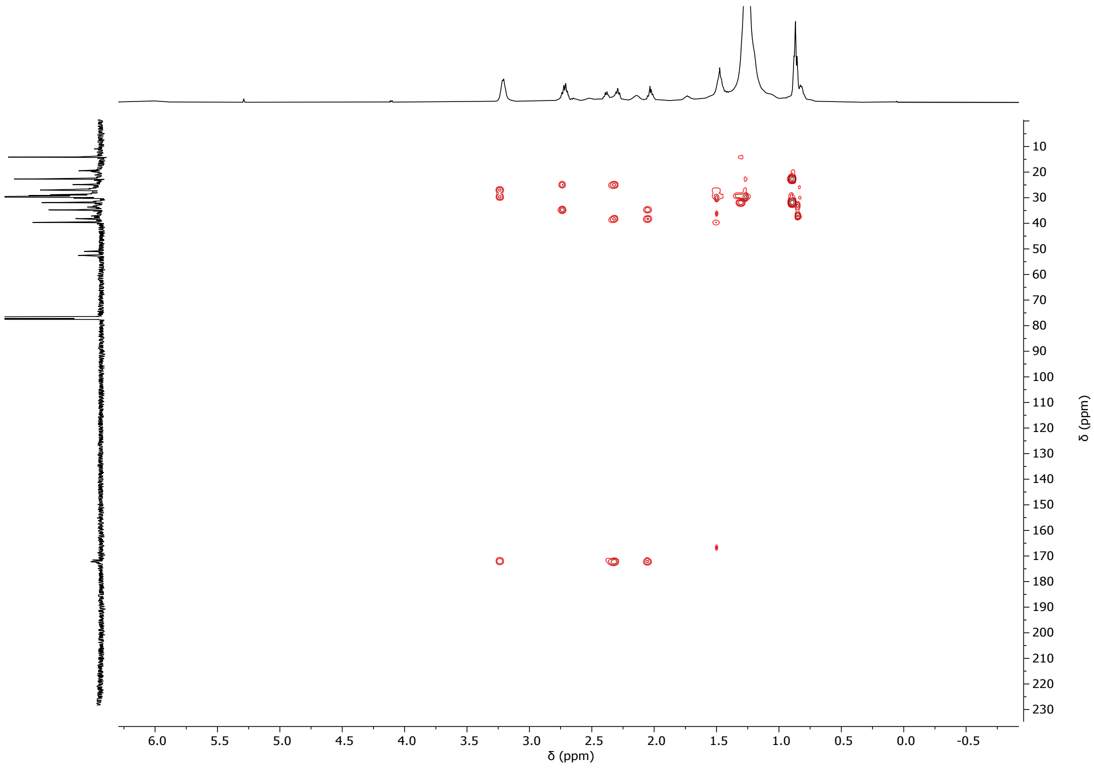

Figure S81. ^{1}H, ^{13}C- HMBC NMR ($CDCl_3$, 298 K) spectrum of product of reaction of **PS-2** with octene-1 catalysed by Blue LEDs (λ_{exc}=440 nm) irradiation.

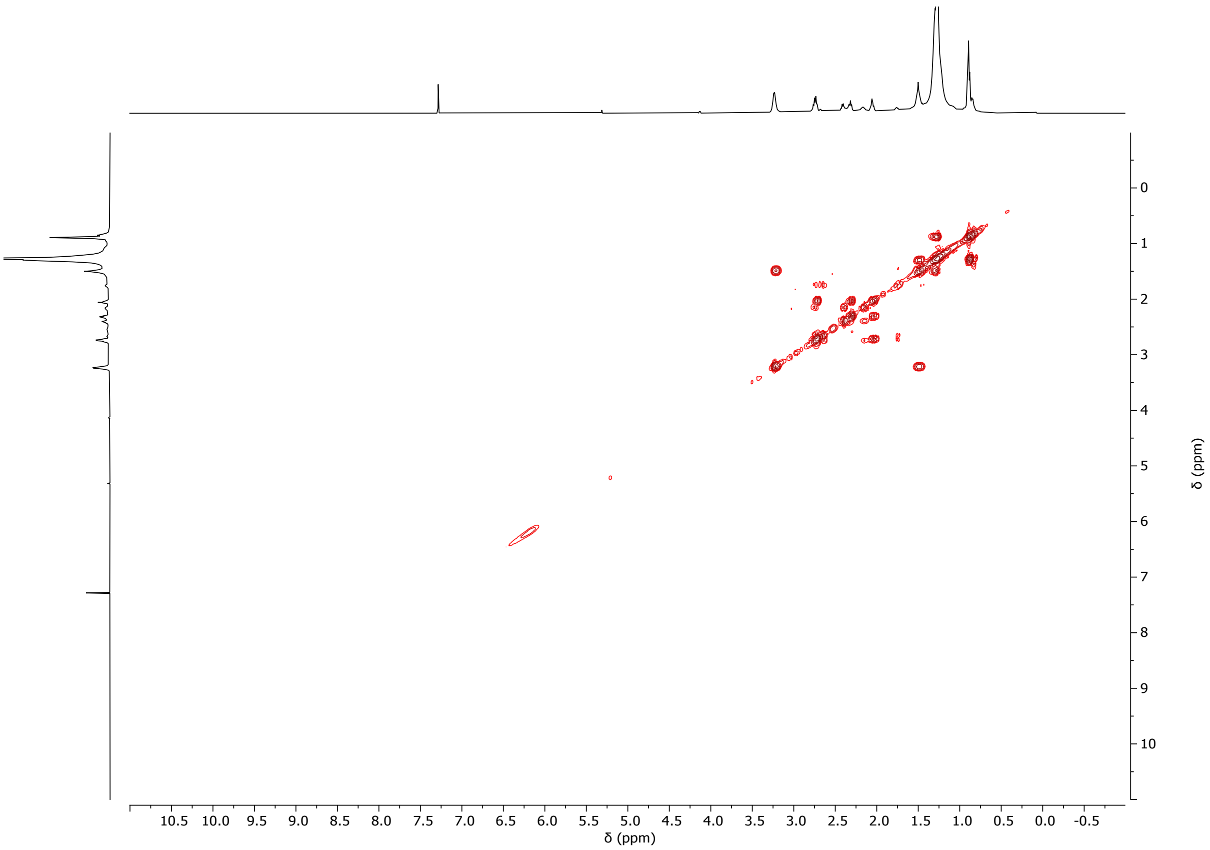

Figure S82. ^{1}H, [13]H- COSY NMR ($CDCl_3$, 298 K) spectrum of product of reaction of **PS-2** with octene-1 catalysed by Blue LEDs (λ_{exc}=440 nm) irradiation.

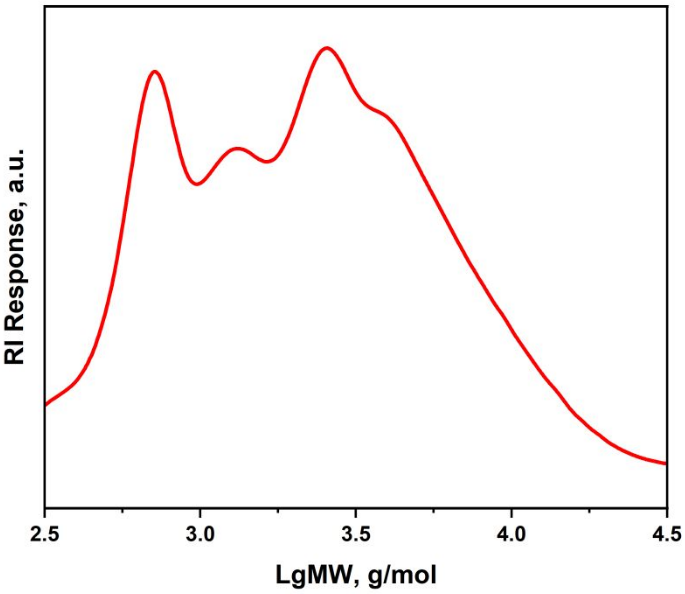

Figure S83. (a) GPC analysis of the sample after blue light catalyzed reaction of **PS-2** with octene-1. M_n: 1.4 kg/mol PDI: 2.5.

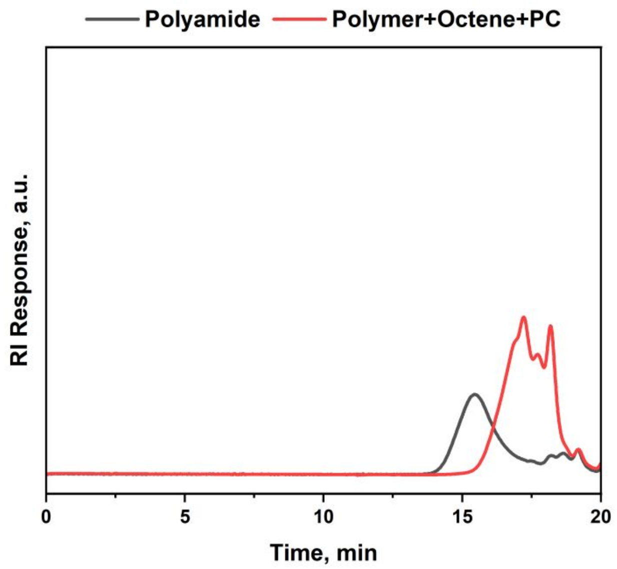

Figure S84. GPC chromatogram of the sample after blue light-catalysed reaction of **PS-2** with octene-1 (red) in comparison with virgin polymer PS-2 (black).

4. Self-healing studies by tensile testing.

4.1 Preparation of dogbone specimen.

The 10% w/v polymeric solution was prepared in NMP (N-Methyl-2-pyrrolidone) by dissolving **PS-2** under continuous stirring at 80 °C until complete dissolution was achieved. The resulting polymeric solution was poured into the preheated mould (60 °C) for casting. After pouring, the mould was placed under vacuum for an hour at 50 °C to remove bubbles and solvent. Subsequently, the oven was turned off, and the mould was left inside the oven for 24 hours, enabling gradual cooling and solidification. After the cooling period, the dogbone shaped sample was carefully removed from the mould (Figure S69).

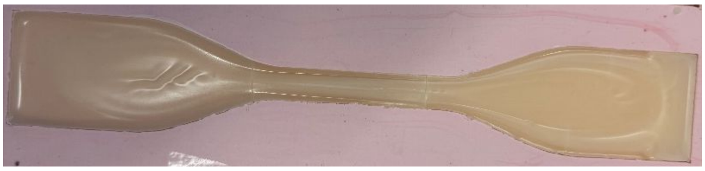

Figure S85. ASTM D638 Type IV standard-compliant dog-bone shape prepared from **PS-2** in silicon mould.

4.2 Self-healing study.

The self-healing of the sample was carried out by bringing the two edges of the cut sample together by applying gentle pressure and heated at 80 °C or 120 °C for 30 minutes (Figure S86). The sample was cooled down by keeping it in the oven at room temperature before the tensile test.

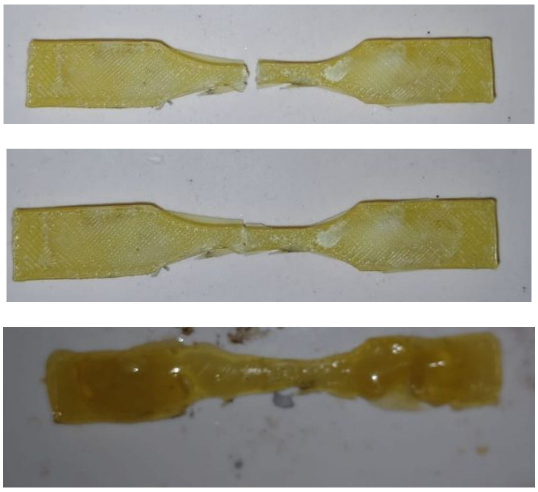

Figure S86. Sample cut in two parts (top), samples brought together at room temperature (middle), image taken just after heating the middle sample at 120 °C (bottom).

4.3 Tensile testing.

Tensile tests were performed at room temperature using an Instron 1195 tensile testing machine equipped with a 50 kN load cell. Before conducting tensile tests, the thickness and width of the gauge sections of the dogbone-shaped sample (ASTM D638, Type IV) [2] were precisely measured using a digital calliper for accurate calculation of stresses and strain. Additionally, the gauge region of each specimen was clearly marked with a black Sharpie to ensure proper alignment during testing and to measure true strain. The strain rate was set at a rate of 20 mm/min. The same conditions were maintained to conduct the tests for pristine and self-healed samples.
The above-mentioned study was conducted three times. The average results have been summarised in Table S6.

After self-healing for 30 minutes at 80°C, the polymer exhibited a recovery of 65% in elongation and 51% in maximum tensile strength. When the self-healing temperature was increased to 120 °C, the

recovery improved significantly to 78% in elongation and 82% in maximum tensile strength (Figure S87)

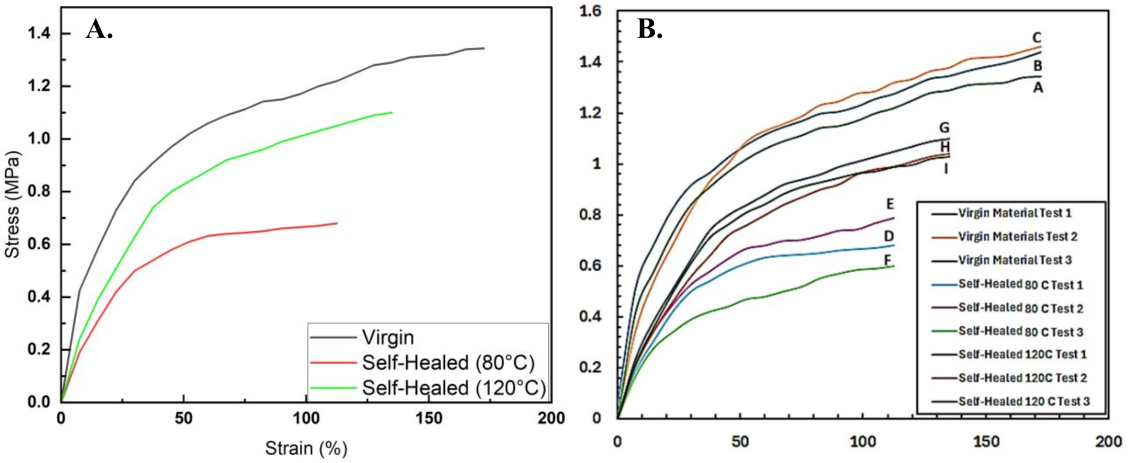

Figure S87. A. Stress-strain curve for virgin and self-healed polymer. B. Stress-strain curve for virgin and self-healed polymer with experiments for reproducibility experiments.

Table S8. Polymer mechanical properties.

Virgin Polymer			Self-healed Polymer (80 °C)			Self-healed Polymer (120 °C)		
Elastic Modulus (MPa)	Elongation (%)	Maximum Stress (MPa)	Elastic Modulus (MPa)	Elongation (%)	Maximum Stress (MPa)	Elastic Modulus (MPa)	Elongation (%)	Maximum Stress (MPa)
2.12	172.5	1.34	1.6	112.5	0.68	1.9	135	1.1

4.4 Self-healing studies by scratch test (qualitative test)

150 mg polymer **PS-2** in 4 mL THF were stirred to get a homogenous solution, which was doctor bladed to prepare a film on the ITO-PET surface. The average thickness of film was found to be ~51.33 µm.

To verify and monitor the self-healing rate of the polymer, it was subjected to scratch test on selected area using a surgical blade and its healing was monitored at different time intervals with constant heating at 80 °C in the oven. Healing was monitored by probing films under an optical microscope (OLYMPUS SZX10) with magnification of 0.63x and 6.3x on selected areas (Figure S72). Effective healing was observed within first 40 minutes, whereas healing rate slows down beyond 40 minutes.

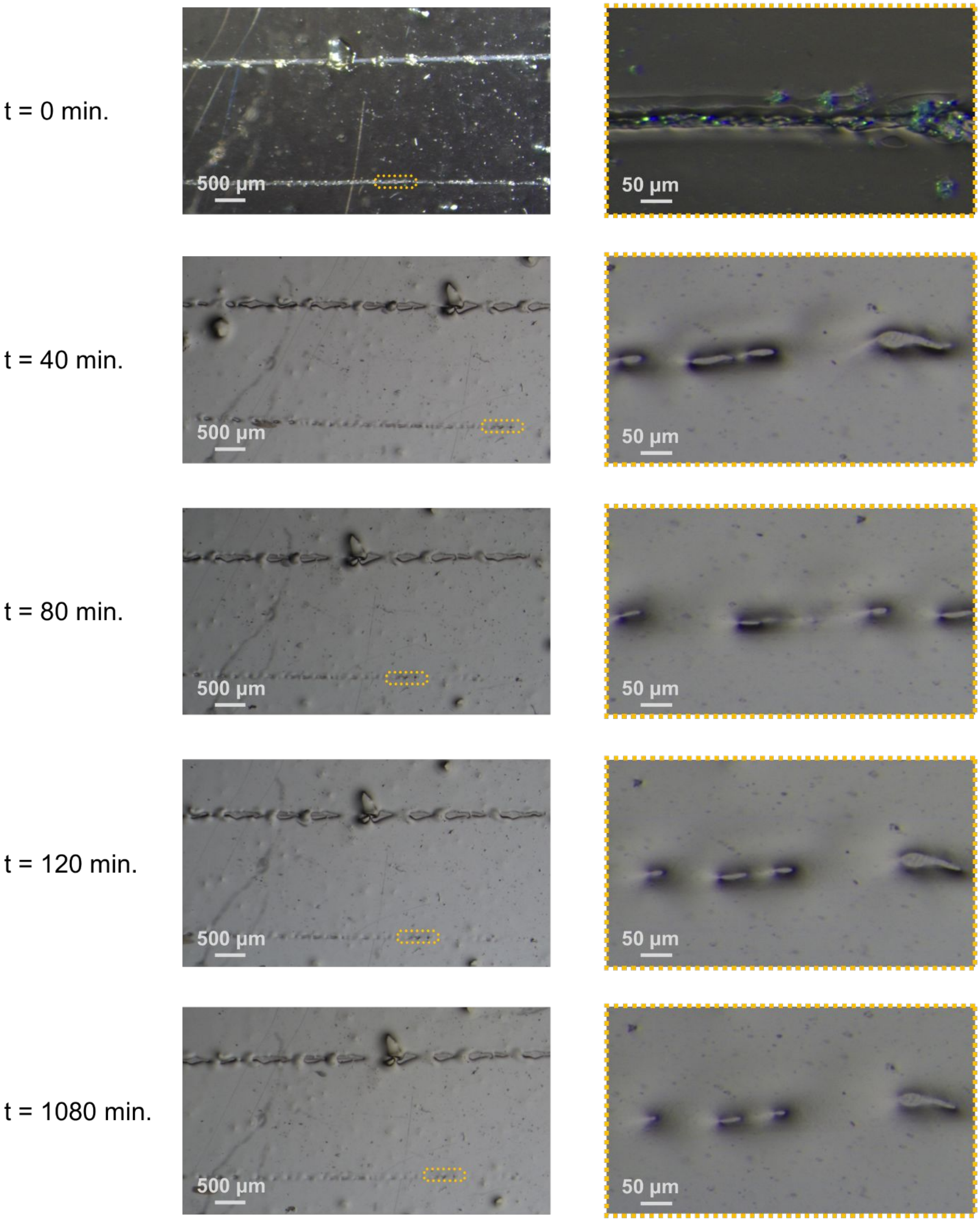

Figure S88. Photographs of qualitative scratch tests and time dependant self-healing of polymer **PS-2** film coated on PET substrate (inset: selected spots under scrutiny).

4.5 Scratch test (in combination with nanoindentation)

Experiment 1. An array of 5 x 1 scratches by ramp load method, to a max load of 500 mN over total scratch length of 300 μm were made. Heating the post scratch sample in convective oven (ensure T uniformity) at 80 °C and 90 °C (for few hours) healed some of the surface damage as depicted below.

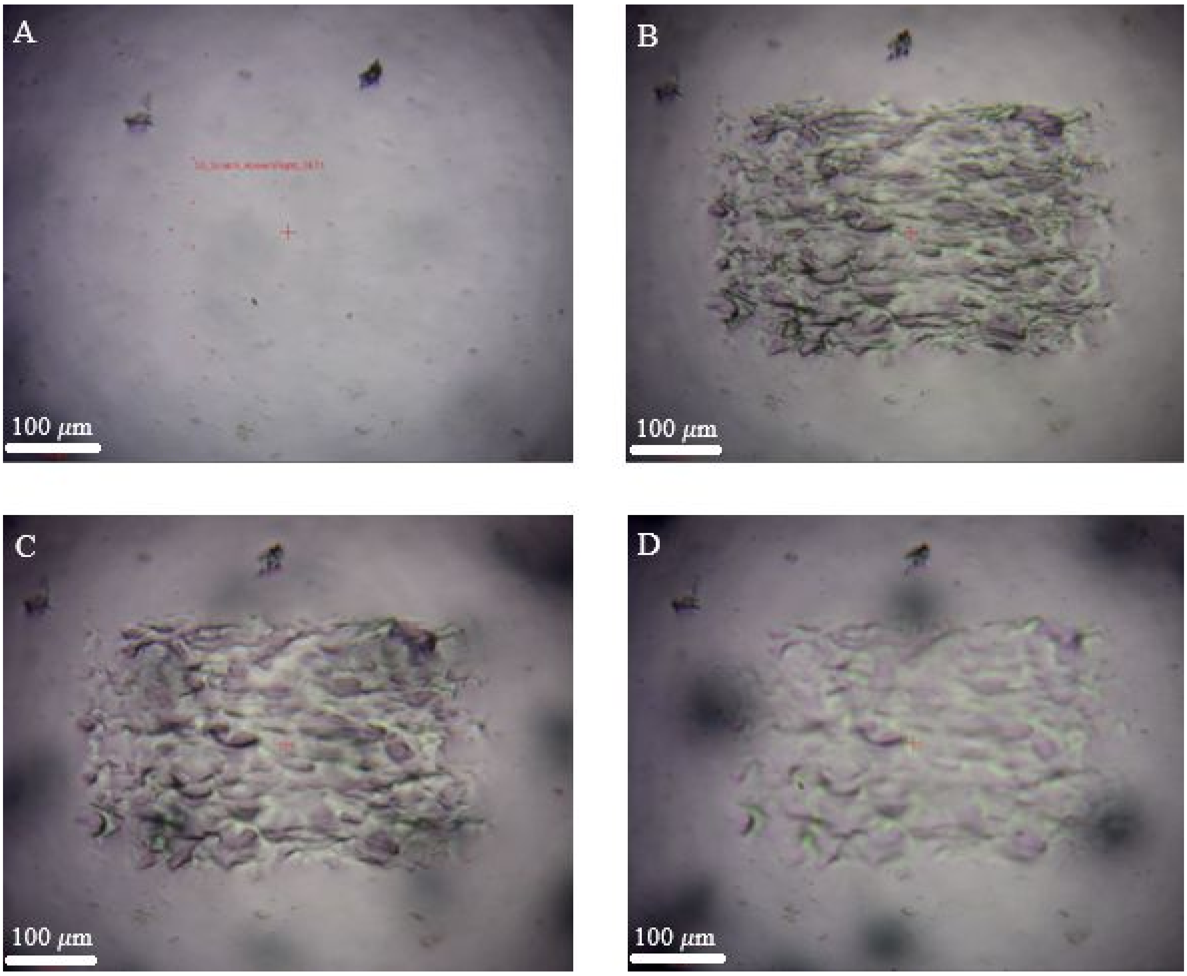

Figure S89. Photographs of scratch tests of **PS-2** by ramp load method. **A.** Before the scratch. **B.** After scratch with load 500 mN over 300 µm. **C.** The scratch after the sample was heated at 80 ° for 2 h. **D.** The scratch after the sample was heated at 80 ° for 2 h, then – at 90 °C for 1 h, then – at 100 °C for 3 h.

Experiments 2. 2 x 1 Scratches using a reduced constant load of 5 mN, over a shorter scratch length of 200 μm were made. The scratches were spaced further apart to avoid overlapping. Followed by "healing" in oven at 80 °C and higher temperatures (90–100 °C) to yield more tangible recovery.

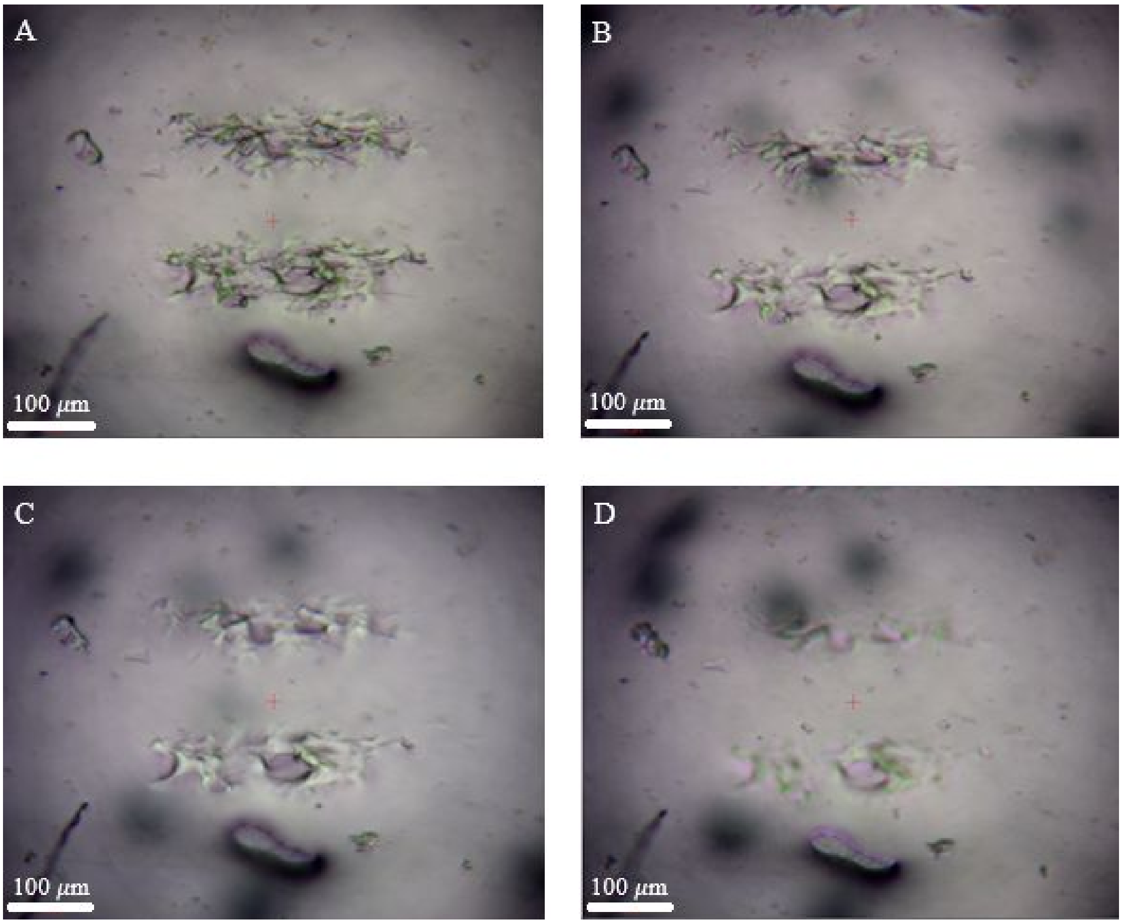

Figure S90. Photographs of scratch tests of **PS-2** by ramp load method. **A.** The sample after a scratch with load 5 mN over 200 μm. **B.** The scratch after the sample was heated at 80 °C for 2 h. **C.** The scratch after the sample was heated at 80 °C for 2 h, and then – at 90 °C for 1 h. **D.** The scratch after the sample was heated at 80 °C for 2 h, then -at 90 °C, and then – at 100 °C for 3 h.

Experiment 3. A single scratch test, at constant load of 5 mN over distance of 300 μm was made. See below for nanoscratch load-displacement curve and the surface recovery after heating at 100 °C for 3 hours.

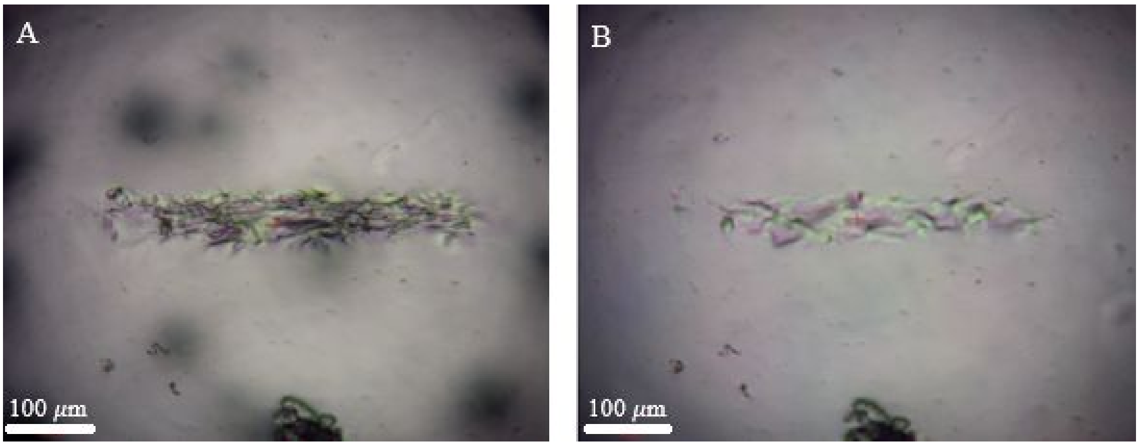

Figure S91. Photographs of scratch tests of **PS-2** by ramp load method. **A.** The sample after a scratch with load 5 mN over 300 μm. **B.** The scratch after the sample was heated at 100 °C for 3 h.

4.6. NMR analysis of PS-2 before and after self-healing studies.

A polymer slice size of 1 cm^2 was cut in half and left in the oven at 80 °C so that two pieces of the material were in a physical contact. After 1.5 h the two pieces completely merged with each other and was taken out of oven. The resulting material was analysed with ^1H and ^{13}C NMR.

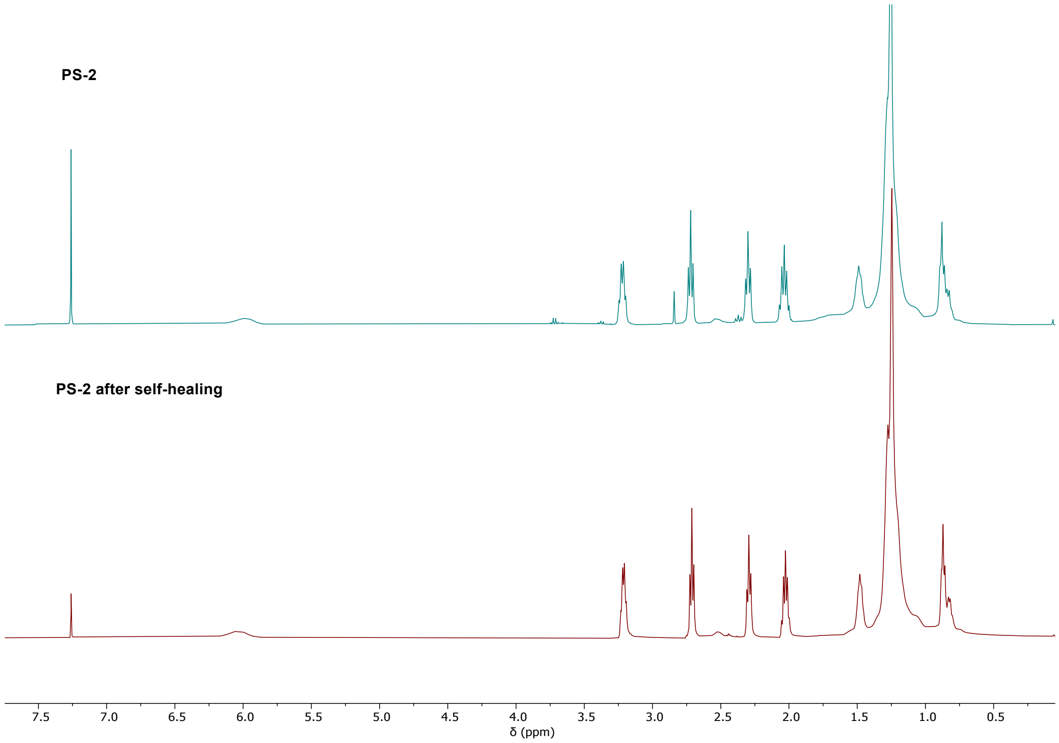

Figure S92. ^1H NMR (500 MHz, $CDCl_3$, 298 K) NMR spectra of **PS-2** before (above) and after (below) self-healing.

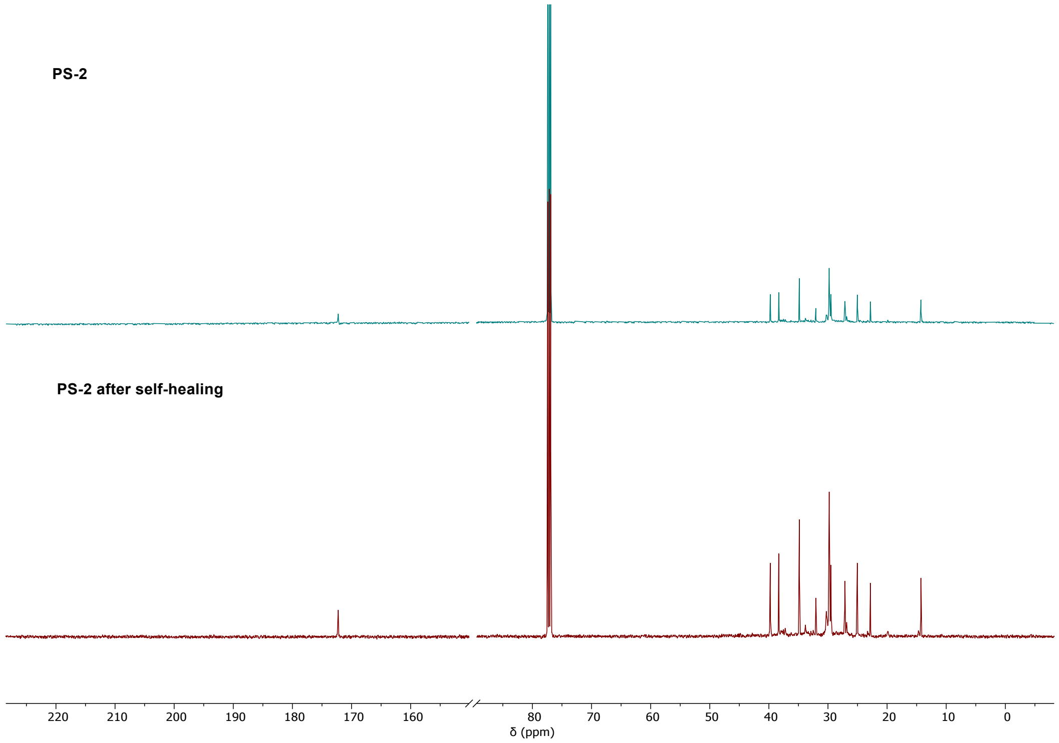

Figure S93. $^{13}C\{^1H\}$ NMR (125 MHz, $CDCl_3$, 298 K) NMR spectra of **PS-2** before (above) and after (below) self-healing

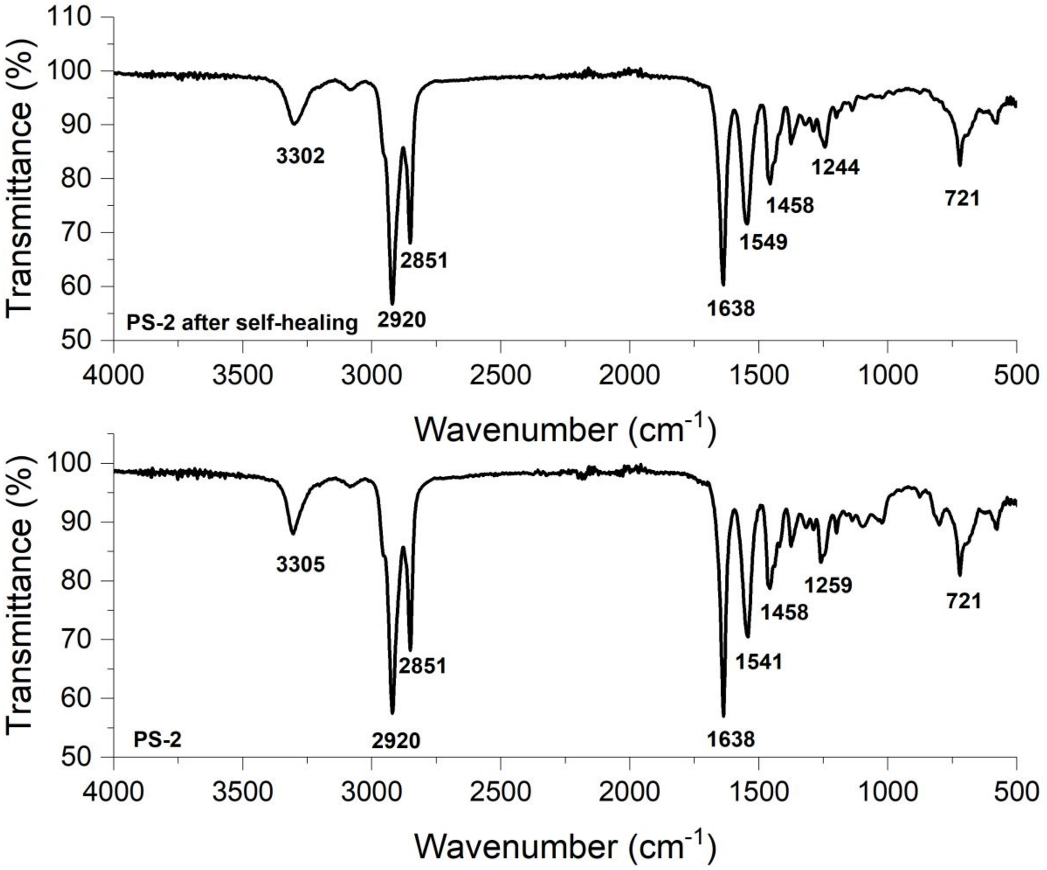

Figure S94. FT-IR (ATR) spectra of **PS-2** before (below) and after (above) self-healing

5. Triboelectric Nanogenerator (TENG) Performance of PS-2 Sample.

5.1 Fabrication of PS-2/Kapton TENG

150 mg of **PS-2** was added to 4 mL of THF at room temperature and stirred to form homogeneous solution. The solution was then casted onto the plasma activated Indium tin oxide (ITO)-coated polyethylene terephthalate (PET) surface using a doctor blade at a fixed height to obtain a thin film with unform thickness. Fast evaporation of the low-boiling THF solvent leaves the dried film with uneven texture, as seen in Figure S95. The obtained thin film has a thickness of ~50 µm. On the counter electrode, Kapton tape was directly pasted onto the ITO-coated PET substrate. Copper wires of identical length and make were attached to both electrodes *via* copper tapes. To allow full contact, soft rubber sheets were placed underneath the PET substrates as buffer layers. The electrode surface rendered nominal contact area of the devices is 3 x 3 cm^2. The image of the TENG devices is shown in Figure S96.

5.2 TENG Measurements

All TENG measurements were conducted on a customised contact-separation test rig (lateral movement type), with the cyclic loading controlled by a power supply, a function generator, and a magnetic shaker (Figure S97). In a standard test, the peak-to-peak amplitude of displacement is kept constant at 1.5 mm, controlled *via* a linear stage. The tapping frequency (surface-to-surface contact frequency) is at 2 Hz and the tapping force is maintained at roughly 80 N determined by a load cell. The output voltage was recorded on an oscilloscope (Picoscope 5442D) with a 100 MΩ high voltage probe (Rigol RP1300H). The output current and charge transfer were both measured by an electrometer (Keithley 6514).

Note S1: The Working Mechanism of TENG. TENG converts mechanical oscillations into electricity through contact electrification and electrostatic induction. In a typical contact separation mode TENG, when two materials with different electron affinities collide, charge with opposite polarities form on both surfaces. Once released, the induced potential difference across the gap drives the electrons to flow from the negative electrode to the positive one. Thus, the flow of electrons occurs due to the electric field generated by the separation of charges, and at full separation, both sides reach charge equilibrium, and no charge flow occurs. When both the surfaces brought to mechanical compression (or surface re-contact) again, thus bringing the surfaces closer together reduces the potential difference, reversing the flow of electrons. This process generates alternating current (AC) from the cyclic mechanical motion, which can be further used or stored in the capacitors/ batteries for intended applications.

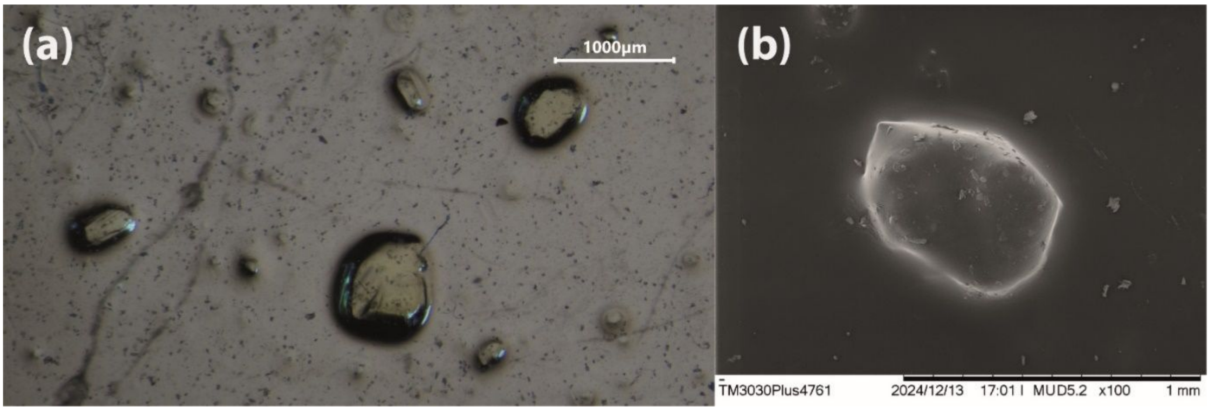

Figure S95. (a) Image of the blade-casted **PS-2** membrane under the stereo microscope. (b) SEM micrograph of a lump on the **PS-2** membrane surface.

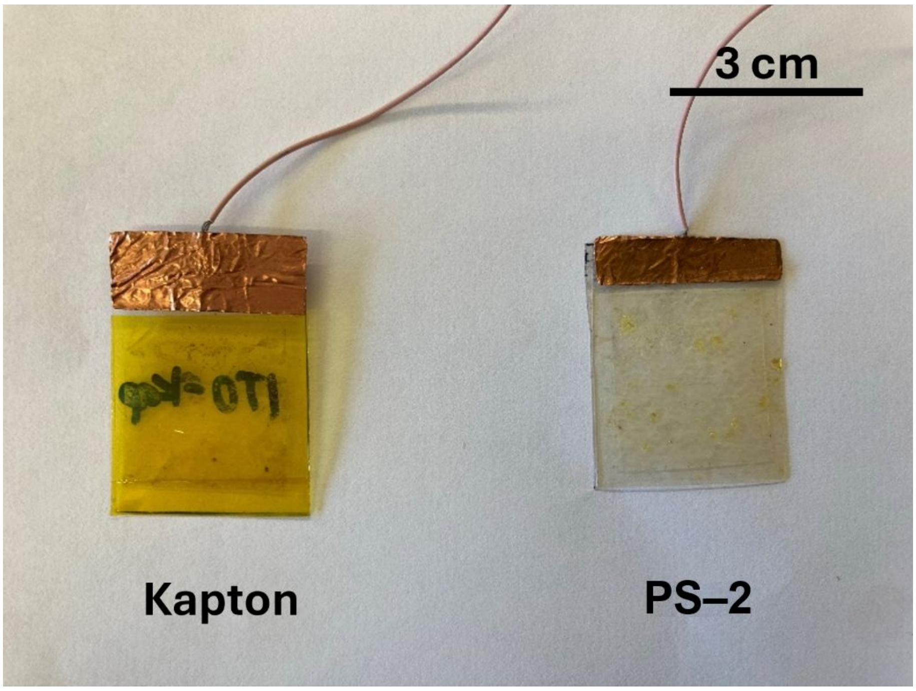

Figure S96. Image of the **PS-2** TENG devices prepared for nanogenerator output measurements.

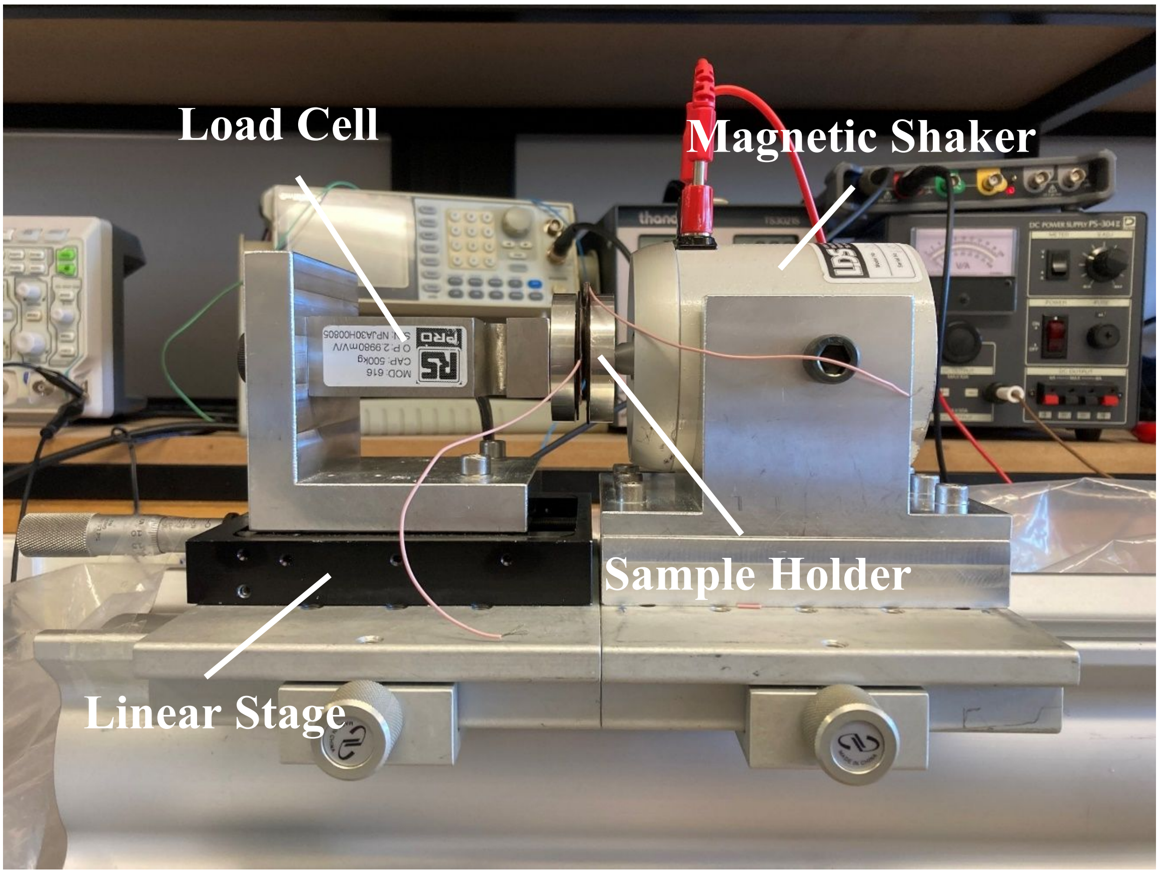

Figure S97. Photo of the contact-separation mode (lateral-type) TENG setup.

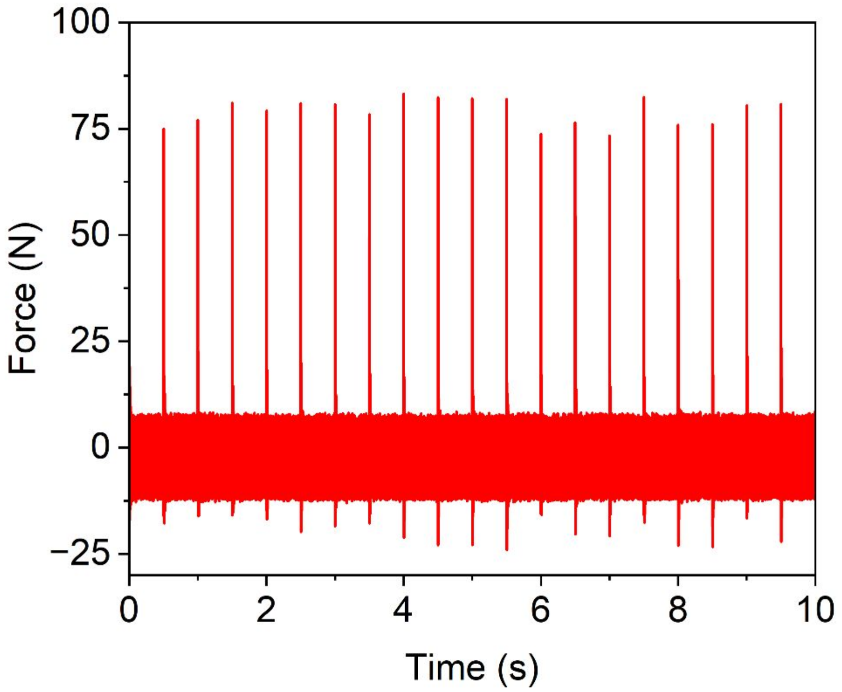

Figure S98. Impact force measured by the load cell in TENG measurements.

Table S9. Output performance of self-healing tribo-positive materials used in TENG devices.

Positive Layer (self-healing)	Negative Layer	V_{oc} (V)	I_{sc} (μA)	Peak Power Density (mW/m^2)	Reference
PCL/Ag NW	PVDF	800V	30	2778	[3]
PU	PVDF	517.5	31.9	2375	[4]
Hydrogel	FEP	33	3	153.2	[5]
ENR/CHI/CNC biocomposite	Teflon	107.7	10.6	156	[6]
PMBEug-OH	PDMS	75	6.2	148.5	[7]
PLMBE	PDMS	40	0.2	2.4	[8]
PI/GP vitrimer composite	PTFE	1320	17.1	2571	[9]
Bio-based PU	PMMA	161	0.15	23.7	[10]
Graphene-based vitrimeric ink	Teflon	452	39	2240	[11]
PS-2	Kapton	80.7	6.4	239	**This work**

References

1. Li, J., Yang, X.-L., Liu, Y.-H., Wu, W.-X., Liu, B.-Y., Wang, N., Yu, X.-Q. J. Mater. Chem. B 2018, 6 (43), 6993-7003.
2. Miller, A., Brown, C., & Warner, G. Guidance on the use of existing ASTM polymer testing standards for ABS parts fabricated using FFF. *Smart and Sustainable Manufacturing Systems*, 2019, *3*(1).
3. N. Luo, Y. Feng, D. Wang, Y. Zheng, Q. Ye, F. Zhou, and W. Liu, *ACS Appl. Mater. Interfaces* **2020**, *12*, 30390.
4. W. Sun, N. Luo, Y. Liu, H. Li, and D. Wang, *ACS Appl. Mater. Interfaces* **2022**, *14*, 10498.
5. K. Zhao, H. Lv, J. Meng, Z. Song, C. Meng, M. Liu, and D. Zhang, *ACS Omega* **2022**, *7*, 18816.
6. O. Somseemee, P. Sae-Oui, and C. Siriwong, *Cellulose* **2022**, *29*, 8675.
7. K.-X. Hou, X. Dai, S.-P. Zhao, L.-B. Huang, and C.-H. Li, *Nano Energy* **2023**, *116*, 108739.
8. H. Wang, Y. Yin, Z. Su, C. Chen, L. Zhang, C. Wang, W. Yang, Y. Huang, P. Xu, P. Ma, T. Liu, and P. Ma, *Adv. Funct. Mater.* **2023**, *34*, 2311649.
9. A. Rajabi-Abhari, P. Li, M.H. Bagheri, A.A. Khan, C. Hao, N.R. Tanguy, D. Ban, L. Yu, and N. Yan, *Nano Energy* **2024**, *131*, 110306.
10. X. Zhang, Z. Jiang, X. Zhang, Y. Xie, and Z.X. Zhang, *Chem. Eng. J.* **2025**, *514*, 163012.
11. S. Sharma and T. Mondal, *Small* **2025**, *21*, 2500481.
